# Prenatal exposure to particulate matter and term low birth weight: systematic review and meta-analysis

**DOI:** 10.1007/s11356-023-26831-7

**Published:** 2023-04-14

**Authors:** Jing Liu, Yuanmei Chen, Die Liu, Fang Ye, Qi Sun, Qiang Huang, Jing Dong, Tao Pei, Yuan He, Qi Zhang

**Affiliations:** 1 Department of Pediatrics, China-Japan Friendship Hospital, Beijing, China; 2 Graduate School of Peking Union Medical College, Chinese Academy of Medical Sciences, Beijing, China; 3 State Key Laboratory of Resources and Environmental Information Systems, Institute of Geographical Sciences and Natural Resources Research, Chinese Academy of Sciences, Beijing, China; 4 University of Chinese Academy of Sciences, Beijing, China; 5 National Research Institute for Family Planning, Beijing, China; 6 National Human Genetic Resources Center, Beijing, China

**Keywords:** Term low birth weight, TLBW, PM_2.5_, PM_10_, Systematic review, Meta-analysis

## Abstract

To evaluate the relationships between maternal particulate matter exposure and offspring birth weight. Studies were categorized into three subgroups: term low birth weight (TLBW) among full-term births and all births (regardless of gestational age) and low birth weight (LBW) among all births, based on the search results of MEDLINE and the Web of Science from the inception of the database to April 2022. Subgroup analyses were conducted based on the economic status, region, exposure assessment, risk of bias, and adjustment. Sixty-one studies involving 34,506,975 singleton live births in 15 countries were analyzed. Overall, the risk of bias for most studies (75%) was low. In 39 of 47 term birth studies, the pooled odds ratio of TLBW among term births for per interquartile range (IQR) increases throughout the entire pregnancy was 1.02 (1.01 to 1.03) for PM_2.5_ and 1.03 (1.01 to 1.05) for PM_10_ after adjustment for covariates. No significant relevance was detected across each trimester period for PM_2.5_. A stronger effect was observed during the second trimester (1.03, 1.01 to 1.06) for PM_10_. There was no increased risk of TLBW in all births associated with IQR increases in PM_2.5_ and PM_10_. LBW was associated with PM_2.5_ exposure in 4 of 7 studies, but statistical heterogeneity was considerable. In the TLBW subgroup analysis, the effects of PM_2.5_ and PM_10_ were both greater in studies conducted in advanced countries, studies with low bias, and studies that adjusted for maternal age, infant sex, and parity. Stronger effects were present for PM_2.5_ exposure collected from monitoring stations and PM_10_ exposure interpolated from the inverse distance weighting model. TLBW may be associated with prenatal exposure to particulate matter, but no critical windows were identified. Stronger associations were observed in advanced countries. Future original study designs need to consider the impact of different exposure assessment modalities and all possible confounding factors.

**Supplementary Information:**

The online version contains supplementary material available at 10.1007/s11356-023-26831-7.

## Introduction

The global illness burden attributable to particulate matter (PM) exposure has been exacerbated (Lim et al. 2012; GBD 2019 Risk Factors Collaborators 2020). PM is a component of the atmosphere consisting of both solid particles and liquid droplets, mainly produced by human activities such as manufacturing, transportation, cooking, fuel combustion, and biomass burning (Daellenbach et al. 2020; U.S. Environmental Protection Agency 2019). Increasing epidemiologic evidence is indicating that ambient PM pollution is linked to unfavorable health impacts including total (nonaccidental) mortality (Franklin et al. 2007; Thurston et al. 2016), increased hospital admissions for major cardiovascular disease (Tian et al. 2019), a contributor to deaths from chronic obstructive pulmonary disease (Li et al. 2020a, b), and adverse birth outcomes (Yuan et al. 2019).

Birth weight is a significant indicator of the health and nutrition of both mother and infant. In 2015, low birth weight (LBW) affected about 15% of all newborns around the world (Blencowe et al. 2019), jeopardizing their survival, health, and development. Triggers of fetal growth restriction, particularly in vulnerable populations, are associated with progress toward the global nutrition goal of reducing the prevalence of LBW by 30 percent between 2012 and 2025 (WHO 2014). In addition to several determinants including hereditary, socioeconomic, dietary, and maternal complications during pregnancy (Kramer 1987), several studies have also correlated birth weight with prenatal exposure to particulate matter (Hung et al. 2023; Rosa et al. 2017a).

Several possible mechanisms could explain the impact of prenatal PM exposure on maternal and newborn health. One likely pathway manifested through inflammatory stress and endothelial function is the cardiovascular mechanisms (Brunst et al. 2018; Grevendonk et al. 2016; Rosa et al. 2017b; Kannan et al. 2006). More recent studies indicate that air pollutants (carbon nanoparticles) inhaled by pregnant women can cross the placenta and enter multiple organs such as the lungs, liver, and brain of the fetus (Bongaerts et al. 2022). Epigenetic marks of gestational PM exposure discovered in the placenta and cord blood, such as DNA methylation, histone H3 modifications, and telomere length, may also play an important role (Aguilera et al. 2022; Zhao et al. 2021; Zheng et al. 2017; Martens et al. 2017).

Despite the establishment of pathways and biological processes, causality determinations for PM exposure and birth outcomes were classified as “suggestive, but insufficient or inadequate to infer” by the United States Environmental Protection Agency (EPA) based on current research (U.S. Environmental Protection Agency 2019). Large birth cohort studies seem to document a consistent positive connection between PM exposure and term-LBW(TLBW). However, some analyses were limited to pregnant women living near air monitoring stations (Liang et al. 2019; Lavigne et al. 2018; Araban et al. 2012; Wilhelm et al. 2012), which may limit the applicability of the study findings to broader populations. It is not determined whether the inclusion of macrosomia (defined as birth weight > 4000 g) and post-term birth (≥ 42 weeks gestation) attenuate the main association. In addition, the lack of standardized assessment methods may increase maternal exposure assessment errors and differences in health effect estimates. Traditional approaches to exposure measurement, such as fixed-site monitoring, the land use regression (LUR) model, the inverse distance weighting (IDW) spatial interpolation algorithm, the dispersion model, and the Bayesian model, fail to account for spatial heterogeneity and the individual difference of time-activity patterns, which may be a source of between-study heterogeneity. Furthermore, current systematic reviews and meta-analyses that have not extensively controlled for potential confounders, including maternal age, gestational age, infant sex, passive smoking, diabetes, and hypertension during pregnancy, did not provide us with risk estimates for the impact of PM exposure on TLBW.

Given these differences, determining the exact relationship between PM exposure and TLBW is urgently needed based on available epidemiological evidence. Previous systematic reviews have summed up the evidence for associations between PM exposure and childbirth outcomes (Li et al. 2017, 2020a, b). We update those reviews, with a focus on the connection between two sizes (PM_2.5_ and PM_10_) of particle exposure and TLBW. We specifically examined how different PM exposures (including interquartile range and 10 μg/m^3^ increments) affect the results. Additionally, we explored the influence of country status, continent, exposure assessment, risk of bias, and adjustment for maternal age, infant sex, and parity on our results.

## Methods

This study was conducted in accordance with the preferred reporting items for systematic reviews and meta-analyses (PRISMA) and the Cochrane Handbook. The protocol was registered in INPLASY (number: 10.37766/inplasy2022.8.0064).

### Data sources and search strategy

A comprehensive search of PubMed and Web of Science was conducted from the database inception until April 7, 2022. Both subject headings and free text terms were searched for two themes of “air pollution” and “LBW” separately (Supplementary Table S1) to increase sensitivity to potentially appropriate studies. Synonymous terms were first combined with the Boolean operator “OR.” These 2 themes were then combined with the Boolean operator “AND.” No restrictions were applied to PubMed. Filters were applied to exclude review articles, meetings, and non-English publications from the Web of Science. All cited references were also checked for potential sources.

### Inclusion and exclusion criteria

The following criteria were used to determine inclusion: (a) study design: birth cohort studies; observational studies; (b) study population (pregnant women and their singleton live births); (c) prenatal PM exposure was assessed using ground-level monitoring stations or validated exposure models (μg/m^3^); (d) PM_2.5_ and PM_10_ were treated as linear or quartile terms; (e) pregnancy outcomes were defined as dichotomous variables: TLBW(≥ 37 weeks and < 2500 g) or LBW (2500 g); (f) risk estimates were presented as hazard ratios (HRs) or odds ratios (ORs) and 95% confidence intervals (CI) with each specific increment in the PM; (g) if multiple articles reported results drawn from the same source dataset or cohort, we only included the most comprehensive study. Exclusion criteria were: (a) irrelevant studies; (b) time-series study, ecological study, and trial; (c) non-English studies; (d) studies with no standard diagnostic criteria for LBW (not < 2500 g); (e) studies with a rate of LBW < 1%, because we postulated that these studies findings may not be representative or underestimating effects; (f) abstract, review, letter, guidelines, case report, and animal or in vitro studies.

### Study selection

The search results were imported into the reference management software and deduplicated. Titles and abstracts were double screened by four independent authors (JL, YMC, DL, FY) with the observed agreement between reviewers being 97%. Two reviewers (JL, YMC) independently examined the full texts of identified articles against inclusion and exclusion criteria. Disagreements or uncertainty were reconciled by discussion or by a third reviewer (QZ).

### Risk of bias within individual studies

We developed a tailored version of the risk of bias tool (modified-OHAT, Supplementary Table S3) according to the Office of Health Assessment and Translation (OHAT) risk-of-bias questions and the Agency for Healthcare Research checklist (Rooney et al. 2014; Viswanathan et al. 2012), focusing on the bias questions applicable to the environmental health study designs. Two independent reviewers (JL, YMC) applied the tool to perform the quality assessment, and a third reviewer (QZ) settled discrepancies. In particular, we concentrate on the following potential sources of bias: (1) the birth cohort and reproductive data for the study population selection were gathered from the national birth certificate or birth registration database. Extreme gestational age and birth weight data (e.g., < 24 weeks, > 44 weeks, or > 5000 g) were eliminated in order to minimize their effect on the results. (2) Exposure assessment: studies used the geocoded maternal pregnancy residential address instead of infant birth addresses to determine daily prenatal exposure to PM and accounted for residential mobility during pregnancy; no restrictions on residing within 5 or 10 km of a ground monitoring site. (3) Analyses were adjusted for several potential confounding variables (such as maternal age, infant sex, parity, maternal education, and gestational age); studies compared whether there was a difference in birth weight and other parameters between children included in the analysis and those excluded due to missing data. (4) Using a uniform calculation formula, PM exposure or meteorological data are assigned to the entire pregnancy or each trimester; questionnaires or medical records were utilized to collect information on demographics, smoking history, and other factors. (5) More detailed analyses and complete data are provided in the main text or appendix; outcome reporting is not selective. Each item is categorized as having a low, moderate, high, or unclear risk of bias. Overall, the bias of a study was rated high if three or more of the five criteria were satisfied, medium if two, and low if one or fewer were met. Assessments are reported in Supplementary Table S5.

### Data extraction

We developed a structured and pretested standard form to extract the following information: reference (first author, publication year), study characteristics (country and city, duration, design, setting), participant characteristics (inclusion and exclusion criteria, total sample size and the number or rate of LBW or TLBW), exposure assessment (Supplementary Table S4a-c). We also extracted information on any adjustment for covariates (Supplementary Table S7, Fig. S1). We only extracted adjusted ORs or HRs with 95% CI from the single-pollutant model. Furthermore, the detailed description of the results in the original article was extracted (Supplementary Table S6a-c). Eligible articles were extracted independently (JL, YMC) in duplicate, and all were reconciled by QZ. Attempts were made to contact authors for information that was not reported in the study.

### Data synthesis and analysis

To reduce clinical heterogeneity, we grouped outcomes into three categories based on the reference birth group: TLBW among full-term births (≥ 37 weeks’ gestational age), TLBW among all births (regardless of gestational age), and LBW among all births. “Adjusted” studies were those that accounted for all three of the most significant variables (age, infant sex, and parity).

Inverse variance-weighted random effect meta-analysis was used to pool estimates. We used the most adjusted reported odds ratio when more than one potential confounding was reported. The increments were not transformed and classified into two groups (per interquartile range and per 10 μg/m3). We analyzed PM exposure for the entire pregnancy, the first, second, and third trimesters. *I*^2^ statistic was used to quantify heterogeneity. Associations between PM and TLBW were quantified and summarized (Supplementary Fig. S2-S6).

We paid specific attention to critical gestational periods or windows when exposure to PM might have had the largest impacts. Stratified analyses by the economic status of the country (a developing or advanced country according to the International Monetary Fund (2022), region of study (Asia, Europe, South America, North America), exposure assessment (LUR model, monitoring stations, IDW, dispersion model, Bayesian model), risk of bias (low, moderate, or high), and adjustment (yes versus no) were conducted to further evaluate the effects of PM on TLBW among term births. Next, we performed sensitivity analyses to evaluate the robustness of our findings by removing individual research from the initial analysis and recalculating the pooled effect (Supplementary Fig. S9). We investigated publication bias visually using funnel plots and numerically with Egger’s regression test (Supplementary Fig. S7-S8). Stata version 17 (StataCorp) was used for all statistical analyses. All tests were two-tailed with a significance threshold of 0.05.

## Results

**Fig. 1:**
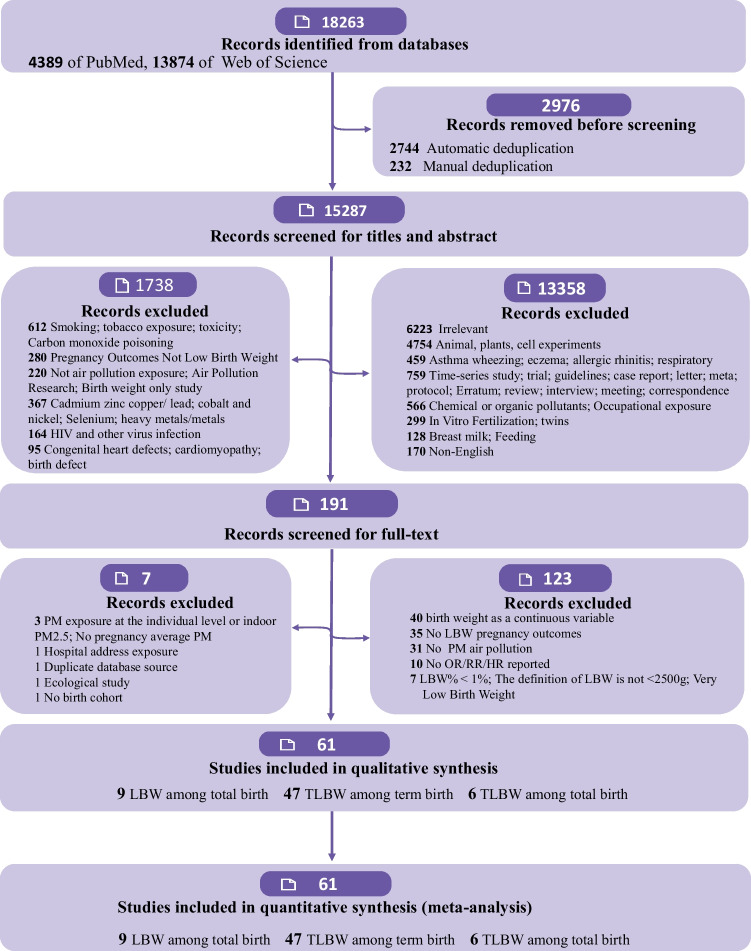
PRISMA flow diagram

Fifteen thousand two hundred eighty-seven records were identified after removing duplicates from 18,263. Of these, 15,096 articles were excluded after the first screening based on abstracts or titles, leaving 191 articles for full-text review. Furthermore, 130 articles were excluded because of predetermined criteria. Eventually, 61 articles involving 34,506,975 singleton live births set in 15 countries were selected. Most studies covered the impacts of PM on TLBW among term birth (*n* = 47); six presented data on TLBW among all birth; and nine on TLBW among all birth. Figure 1 depicts the study’s flowchart.

### Characteristics of included studies

Nearly two-thirds (40 studies, 66.7%) of these studies occurred in advanced countries. Most studies were conducted in North America (31 studies, 50.8%, 26 from America) and Asia (17 studies, 27.9%, 10 from China), with only five studies in South America, four studies in Europe, two studies in Oceania, and one study in Africa. Thirty-five studies found a link between PM_2.5_ and TLBW in term births (*n* = 31) or all births (*n* = 4) and between PM_10_ and TLBW in term births (*n* = 25) or all births (*n* = 3). There are seven studies on the effects of PM2.5 on LBW, but only three on the effects of PM_10_ on LBW. Overall, 46 (75.4%) studies exhibited a low risk of bias, 11 (18%) a medium risk, and 4 (6.6%) a high risk, resulting in a low risk of bias among all included research. Table 1 summarizes the characteristics of the study population, including the number of TLBW or LBW, country status, continent, exposure assessment, risk of bias, and adjustment. Table 1 provides a summary of the study’s characteristics, including population size, nation status, continent, exposure assessment, risk of bias, and adjustment.

**Table 1:** Characteristic of study population

	PM_2.5_			PM_10_		
Study group	TLBW (35 studies)		LBW (7 studies) (*n* = 1127827)	TLBW (28 studies)		LBW (3studies) (*n* = 23534)
	Term births (31studies) (*n* = 27287377)	All births (4 studies) (*n* = 1297277)		Term births (25 studies) (*n* = 13267404)	All births (3 studies) (*n* = 76285)	
Number of TLBW/LBW	418,048/19,002,369* (2.2%)	47,438 (3.66%)	808,50/934,927* (8.65%)	155,217/6,602,693* (2.35%)	981 (1.29%)	1021 (4.34%)
Country status						
Developing	4,341,313 (15.9%)	123,034 (9.5%)	303,565/934,927 (32.5%)	5,103,513 (38.5%)	6036 (7.9%)	23,534 (100%)
Advanced	22,946,064 (84.1%)	1,174,243 (90.5%)	631,362/934,927 (67.5%)	8,163,891 (61.5%)	70,249 (92.1%)	0
Continent						
Asia	4,335,166 (15.9%)	0	171,971/934,927 (18.4%)	6,273,564 (47.3%)	6036 (7.9%)	23,534 (100%)
Africa	0	0	131,594/934,927 (14.1%)	0	0	0
Europe	569,182 (2.1%)	0	0	568,646 (4.3%)	0	0
Oceania	0	285,594 (22%)	173,720/934,927 (18.6%)	0	0	0
South America	6147 (0.02%)	123,034(9.5%)	0	970,001 (7.3%)	0	0
North America	22,376,882 (82%)	888,649(68.5%)	457,642/934,927 (48.9%)	5,455,193 (41.1%)	70,249 (92.1%)	0
Exposure assessment						
LUR model	4,163,137† (15.3%)	355,843† (27.4%)	0	80,854 †(0.6%)	70,249 †(92.1%)	2144 (9.1%)
AOD-based model	0	0	3692 (0.3%)	0	0	0
Monitoring stations	9,579,369† (35.1%)	70,249 (5.4%)	173,720 (15.4%)	9,820,983 †(74%)	72,767† (95.4%)	2527 (10.7%)
IDW	2,817,819(10.3%)	888,649† (68.5%)	18,863 (1.7%)	2,877,820 (21.7%)	73,758† (96.7%)	18,863(80.2%)
Dispersion model	540,365† (2%)	0	0	636,624† (4.8%)	0	0
Bayesian model	10,254,956 (37.7%)	0	457,642 (40.6%)	0	0	0
ACAG	0	0	473,910 (42%)	0	0	0
Chemical transport model	0	123,034 (9.5%)	0	0	0	0
CATT-BRAMS Model	6147 (0.02%)	0	0	0	0	0
Risk of bias						
Low	25,355,324 (92.9%)	1,011,683 (78%)	978,411 (86.8%)	13,106,893 (98.8%)	70,249 (92.1%)	18,863 (80.2%)
Moderate	1,932,053 (7.1%)	285,594 (22%)	0	160,286 (1.2%)	0	2144 (9.1%)
High	0	0	149,416 (13.2%)	225 (0.0017%)	6036 (7.9%)	2527 (10.7%)
Adjustment‡						
Yes	18,917,704(69.3%)	888,649 (68.5%)	846,817 (75.1%)	8,579,851 (64.7%)	70,249 (92.1%)	21,007 (89.3%)
No	8,369,673 (30.7%)	408,628 (31.5%)	281,010 (24.9%)	4,687,553 (35.3%)	6036 (7.9%)	2527 (10.7%)

*LUR*, land use regression; *IDW*, inverse distance weighting; *ACAG*, atmospheric analysis group; *CATT-BRAMS*, coupled aerosol and trace gas transport model to the Brazilian developments of the regional atmospheric modeling system

^*^Guo (2020), Harris (2014), Fleischer (2014), Basu (2014), Laurent (2013), Bell (2010), Lee (2003), Maisonet (2001), and Morello-Frosch (2010) did not report detailed numbers of TLBW/LBW

^†^There are 3 methods of exposure assessment for Brauer (2008) and Laurent (2013)

^‡^Adjusted for maternal age, infant sex, and parity

### Relationships between prenatal PM exposure and pregnancy outcomes

#### TLBW among term births

**Fig. 2:**
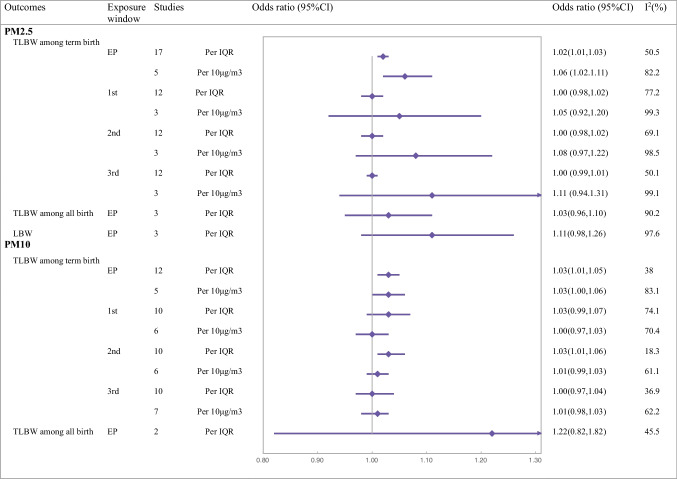
Findings of meta-analysis of association between prenatal exposure to particulate matter and term low birth weight

Thirty-nine studies assessed the impact of PM on TLBW among term births, and the TLBW rate is 2.2% (515,719/23,311,021). There were 31 studies reporting PM_2.5_. TLBW was positively related to PM_2.5_ exposure. The pooled OR for PM_2.5_ exposure during pregnancy was 1.02 (1.01 to 1.03) per IQR and 1.06 (1.01 to 1.11) per 10 μg/m^3^ increase, respectively (Fig. 2) (Fig. S2-a, b). Additionally, we discovered that the probabilities of TLBW rose by 1.03 for each 1 μg/m^3^ increase in PM_2.5_ exposure (1.02 to 1.04; Fig. S2-d). However, no conclusions can be drawn about the specific critical window of PM_2.5_ exposure. Twelve studies provided PM_2.5_ exposure (per IQR increase) for each trimester. Nine studies reported null findings in the first and second trimesters, and only two out of twelve studies found a favorable connection in the third trimester. There was considerable heterogeneity in estimates of the odds of TLBW per IQR increase in PM_2.5_ exposure for each trimester (*I*^2^ > 50%).

There were twenty-five studies reporting PM_10_. TLBW was also positively related to an increased level of PM_10_. The pooled odds ratios of TLBW associated with an IQR change in PM_10_ exposure were 1.03 (1.01 to 1.05), and per 10 μg/m^3^ increase was also 1.03 (1.00 to 1.06) (Fig. 2) (Fig. S5-a, b). Only one study reported an increased TLBW risk of 1.07 (1.01 to 1.03) for a 1 μg/m^3^ increase in PM_10_ (Dibben and Clemens 2015). The second trimester was associated with relatively higher unfavorable correlations (1.03, 1.01 to 1.06, per IQR increase) than the other time periods. Only two of the six studies involving the first and second trimesters of PM_10_ exposure (per 10 µg/m^3^ increase) revealed a significant adverse effect.

#### TLBW among all births

Six studies reported the incidence of TLBW among all births (3.6%, 47,525/1303313). Four studies reported on the association between PM_2.5_ and TLBW during the entire pregnancy, with two studies noting a decrease in TLBW and 2 studies reporting an increase, and pooled analysis suggested no overall effect. Only one study reported that there was a statistically significant increase in risk during the first-trimester window. There were three studies reporting PM_10_. We found that no higher pooled risk of TLBW related to IQR increases in PM_10_ during the entirety of pregnancy or each trimester.

#### LBW

In 8 of 9 studies (one study did not specify the number of LBW), the LBW rate is 8.7% (81,393/939,598). Four out of seven studies found an association between PM_2.5_ exposure and LBW, with considerable statistical heterogeneity. We were unable to evaluate the effect of PM_10_ exposure on LBW owing to insufficient data reported in the studies. No significant variations in the risk of LBW between PM_2.5_ and PM_10_ were observed during the entire pregnancy.

### Subgroup and sensitivity analyses

In TLBW subgroup analysis, the estimated effects of PM_2.5_ and PM_10_ were stronger for infants born in advanced countries, studies with low risk of bias, and those adjusted for maternal age, infant sex, and parity (Table 2 and 3). Stronger effects were present for PM_2.5_ exposure collected from the monitoring stations and PM_10_ exposure interpolated from the inverse distance weighting model. Region-subgroup analysis found substantial relationships between TLBW and PM_2.5_ in North America and Europe and PM_10_ in Asia. Subgroup analyses did not show significant heterogeneity for each trimester, except for the third trimester of the Bayesian PM_2.5_ assessment model. Country status, regions, monitoring station assessment, risk of bias, and adjustment all contributed significantly to the heterogeneity of the first and second trimesters of PM_10_.

**Table 2:** Subgroup analysis for term low birth weight among term birth in studies about PM_2.5_ (per IQR increase)

Exposure window	Country status		Continent			Exposure assessment					Risk of bias		Adjustment*	
	Developing	Advanced	Asia	Europe	North America	LUR model	Monitoring stations	IDW	Dispersion model	Bayesian model	Low	Moderate	Yes	No
EP	2	15	2	2	13	2	8	3	1	3	16	1	11	6
	0.99 (0.96,1.03)	1.02 (1.01,1.03)	0.99 (0.96,1.03)	1.07 (1.01,1.05)	1.02 (1.01,1.03)	1.06 (0.95,1.19)	1.02 (1.00,1.04)	1.00 (0.96,1.04)	1.06 (1.01,1.12)	1.01 (0.99,1.03)	1.02 (1.01,1.03)	0.98 (0.92,1.04)	1.02 (1.01,1.03)	1.02 (0.99,1.05)
1st trimester	2	10	2	1	9	1	4	3	0	4	11	1	7	5
	0.97 (0.87,1.09)	1.00 (0.99,1.02)	0.97 (0.87,1.09)	1.07 (0.88,1.29)	1.00 (0.99,1.02)	1.07 (0.88,1.29)	1.02 (1.00,1.04)	1.01 (0.92,1.10)	NA	0.99 (0.96,1.01)	1.01 (0.99,1.02)	0.92 (0.87,0.98)	1.02 (0.99,1.04)	0.99 (0.96,1.02)
2nd trimester	2	10	2	1	9	1	4	3	0	4	11	1	7	5
	0.95 (0.90,1.00)	1.00 (0.99,1.02)	0.95 (0.90,1.00)	1.19 (0.97,1.45)	1.00 (0.99,1.02)	1.19 (0.97,1.45)	1.01 (0.98,1.03)	0.97 (0.92,1.01)	NA	1.00 (0.97,1.02)	1.00 (0.99,1.02)	0.95 (0.87,1.03)	1.00 (0.98,1.02)	1.00 (0.97,1.03)
3rd trimester	2	10	2	1	9	1	4	3	0	4	11	1	7	5
	0.97 (0.93,1.02)	1.00 (0.99,1.01)	0.97 (0.93,1.02)	1.24 (1.03,1.49)	1.00 (0.99,1.01)	1.24 (1.03,1.49)	1.00 (0.98,1.02)	0.96 (0.92,1.00)	NA	1.01 (1.00,1.02)	1.00 (0.99,1.01)	1.00 (0.94,1.07)	0.99 (0.97,1,02)	1.00 (0.99,1.01)

*EP*, entire pregnancy; *IQR*, interquartile range; *LUR*, land use regression; *IDW*, inverse distance weighting; *NA*, not applicable

^*^Adjusted for maternal age, infant sex, and parity

**Table 3:** Subgroup analysis for term low birth weight among term birth in studies about PM_10_

Exposure window	Increase	Country status		Continent				Exposure assessment				Risk of bias			Adjustment*	
		Developing	Advanced	Asia	Europe	South America	North America	LUR model	Monitoring stations	IDW	Dispersion model	Low	Moderate	High	Yes	No
EP	IQR	4	8	5	2	0	5	1	6	4	1	10	1	1	9	3
		1.02 (0.99,1.05)	1.04 (1.01,1.07)	1.03 (1.00,1.06)	1.06 (0.96,1.18)	NA	1.03 (0.98,1.08)	1.16 (0.98,1.37)	1.02 (0.99,1.06)	1.04 (1.00,1.08)	1.03 (0.99,1.07)	1.03 (1.01,1.05)	1.02 (0.96,1.08)	0.63 (0.37,1.06)	1.03 (1.01,1.06)	1.03 (0.99,1.06)
1st trimester	IQR	4	6	5	1	0	4	1	5	4	0	8	1	1	8	2
		1.00 (0.91,1.10)	1.04 (1.01,1.06)	1.2 (0.96,1.08)	1.00 (0.82,1.22)	NA	1.05 (1.00,1.10)	1.00 (0.82,1.22)	1.05 (1.00,1.10)	1.02 (0.95,1.09)	NA	1.05 (1.02,1.08)	0.93 (0.88,0.99)	0.63 (0.37,1.06)	1.02 (0.98,1.06)	0.88 (0.52,1.49)
	10 μg/m^3^	4	2	2	1	2	1	0	5	0	1	6	0	0	2	4
		1.01 (0.98,1.04)	0.98 (0.89,1.08)	0.99 (0.94,1.05)	1.03 (0.95,1.11)	1.03 (1.00,1.06)	0.93 (0.85,1.00)	NA	1.00 (0.97,1.03)	NA	1.03 (0.95,1.11)	1.00 (0.97,1.03)	NA	NA	0.96 (0.93,0.99)	1.02 (1.01,1.03)
2nd trimester	IQR	4	6	5	1	0	4	1	5	4	0	8	1	1	8	2
		1.01 (0.96,1.05)	1.05 (1.02,1.08)	1.02 (1.00,1.05)	1.20 (0.96,1.48)	NA	1.06 (1.01,1.11)	1.20 (0.96,1.48)	1.03 (1.01,1.05)	1.03 (0.97,1.09)	NA	1.03 (1.01,1.06)	1.00 (0.91,1.09)	0.63 (0.33,1.19)	1.03 (1.00,1.07)	0.90 (0.58,1.38)
	10 μg/m^3^	4	2	2	1	2	1	0	5	0	1	6	0	0	2	4
		1.01 (0.99,1.03)	0.97 (0.90,1.06)	1.00 (0.95,1.05)	1.01 (0.94,1.08)	1.02 (1.01,1.03)	0.93 (0.85,1.02)	NA	1.01 (0.99,1.03)	NA	1.01 (0.94,1.08)	1.01 (0.99,1.03)	NA	NA	0.96 (0.93,0.99)	1.02 (1.01,1.03)
3rd trimester	IQR	4	6	5	1	0	4	1	5	4	0	8	1	1	8	2
		0.99 (0.96,1.03)	1.02 (0.96,1.08)	1.00 (0.97,1.02)	1.26 (1.06,1.51)	NA	1.00 (0.94,1.07)	1.26 (1.06,1.51)	1.00 (0.97,1.03)	0.99 (0.96,1.03)	NA	1.00 (0.97,1.04)	1.01 (0.95,1.08)	0.73 (0.32,1.64)	1.00 (0.96,1.05)	1.01 (0.97,1.05)
	10 μg/m^3^	4	3	2	1	2	2	0	6	0	1	7	0	0	3	4
		1.00 (0.97,1.03)	1.03 (0.99,1.08)	0.99 (0.98,1.00)	1.04 (0.98,1.11)	1.01 (0.93,1.10)	1.03 (0.97,1.08)	NA	1.00 (0.98,1.03)	NA	1.04 (0.98,1.11)	1.01 (0.98,1.03)	NA	NA	0.99 (0.95,1.03)	1.01 (0.98,1.05)

*EP*, entire pregnancy; *IQR*, interquartile range; *LUR*, land use regression; *IDW*, inverse distance weighting; *NA*, not applicable

^*^Adjusted for maternal age, infant sex, and parity

Almost most primary studies didn’t significantly influence the pooled estimate when a study was removed, demonstrating that no single study had a major effect on the pooled estimate. However, the effects became meaningful for TLBW in the second trimester of PM_10_ (10 μg/m^3^ increase) when the study of Mueller et al. was deleted. The absence of nonsymmetry in funnel plots in the included studies indicated low reporting bias (Fig. S7). The Egger test results revealed no indication of a small study impact (*P* > 0.05, Fig. S8).

## Discussion

### Principal findings

This meta-analysis of 61 studies from 15 countries, including 34,506,975 singleton live births, confirms that there may be a correlation between prenatal exposure to PM_2.5_ and PM_10_ and the risk of TLBW in offspring. The correlation with TLBW among term births in some analyses shows this outcome rising by about 1.02-fold every IQR increase in PM_2.5_ and 1.03-fold in PM_10_. The probability of TLBW was not statistically significantly increased with exposure to PM_2.5_ and PM_10_ during the entire pregnancy when all births (including preterm and term births) were included in the pooled analysis. Associations of PM exposure with LBW appear to be weaker and less inconsistent than those with TLBW. Only 4 of 7 studies that assessed LBW and PM_2.5_ found an association. Three studies evaluating the association between LBW and PM_10_ have reported conflicting results.

Many studies have revealed relationships between different increments of PM and TLBW, such as IQR, 1 μg/m^3^, 5 μg/m^3^, 10 μg/m^3^, 25th percentile, and high (75th or 90th percentile) vs low exposure (25th or 10th percentile). This makes it difficult to pool the specific effects of single pollutants. However, in this study, we used two exposure indicators for PM exposure, the IQR and 10 μg/m^3^ increase. We discovered that both PM2.5 and PM_10_ exposure indicators were linked with TLBW risk.

We were unable to discern clear windows of vulnerability during pregnancy, except that PM_10_ exposure in the second trimester appears most important for TLBW among term births. In other studies, the associations of early pregnancy higher ambient particulate matter with smaller fetal growth parameters, such as the biparietal diameter of the abdominal circumference (AC) and birth weight, also appear robust (Aguilera et al. 2010; Hansen et al. 2008; Leung et al. 2022). While most studies suggest that ambient air pollution effects on birth weight seem to occur in the first and third trimesters (Dadvand et al. 2014; Lavigne et al. 2018; Smith et al. 2017; Wu et al. 2018), a few studies refer to the second and third trimesters (Ha et al. 2014, 2017). Particles may affect fetal development differently at various gestational phases. Mechanistic analyses will be required to explore these possibilities due to the results derived from observational studies.

Associations between PM_2.5_ and PM_10_ exposure and TLBW appear especially pronounced among infants born in advanced countries. Note, however, that most previous studies included in our analysis come from high-resource countries. 15 of the 17 studies reporting PM_2.5_ exposure and TLBW, as well as 8 of the 12 studies, were for developed countries. As a matter of fact, pregnant women in low and middle-income regions might be at particular risk from air pollution, including household air pollution and ambient air pollution (Cohen et al. 2017; Lee et al. 2020). Therefore, more studies on low- and middle-income settings would be welcome to generate robust worldwide estimates in the future.

We found a generally higher effect size of TLBW for PM_2.5_ in North America and Europe than in Asia and for PM_10_ in Asia than in Europe and North America. Variations in the types and origins of PM could have differential health impacts. Firstly, PM_2.5_ in some areas may be dominated by industrial emissions and fuel combustion whereas secondary PM derived from the precursors SO2, nitrogen, and ammonia (NH3), accounts for the bulk of PM_2.5_ mass (U.S. Environmental Protection Agency 2019). Studies have revealed a wide variety of birth weight-reducing effects among PM_2.5_ constituent chemicals. Vanadium, sulfur, sulfate, iron, elemental carbon, and titanium exposure were related to the greatest decreases in birth weight (Basu et al. 2014). Another point that should not be overlooked is that different targets for regional policies hint at different roles played by different regions in health risk reduction. Other factors, such as access to medical services, may exacerbate disparities in health between regions.

Effect estimates of PM_2.5_ and PM_10_ on TLBW varied according to the exposure model in our analysis. The results are more reliable for PM_2.5_ with monitoring station data and for PM_10_ interpolated from the inverse distance weighting model. Different exposure assessment methods, whether the fixed-site monitoring stations or the modeling approaches, such as spatial interpolation methods, LUR, and dispersion models, were used to assign ambient air pollution levels to large populations. However, these methods often fail to integrate important information into the individual activity patterns and consider ambient pollutant infiltration into the indoor environment. There may be less of a relationship between the estimated exposure and the health effect of interest if PM infiltrates a building’s envelope and alters the PM concentration’s temporal variability within (Ambade et al. 2021; Meng et al. 2007; Mohammed et al. 2016; U.S. Environmental Protection Agency 2019).

### Strengths and limitations of the study

Our analysis had a larger sample size, a better subject grouping pattern, and used more rational exposure increment pooling methods than previous studies. Also, we assessed associations between PM exposure at individual time points during pregnancy and TLBW in order to identify windows of vulnerability. Notably, we considered a number of confounding factors adjusted for in the main adjusted models of each study and identified whether confounders were adjusted or not significantly affect the pooled effect size. Moreover, we developed a risk of bias tool capable of being applied to environmental health problems to enhance impartiality. For these reasons, the findings from the current pooled analysis are stouter than the conclusions regarding the impact of prenatal exposure to PM and TLBW in the previous meta-analyses.

It should be noted that our study had some limitations. We repeated the analysis using PM_2.5_ and PM_10_ data and stratified by exposure assessment. While each of the methods has attractive features, all have drawbacks. Additionally, we used single-pollutant models instead of two- or multiple-pollutant models. We also cannot exclude the possibility that other air pollutants confound the observed associations. Also, some maternal factors (e.g., education, body mass index before pregnancy, weight change during pregnancy, smoking status, prenatal care, marital status, alcohol use after conception, drug use, pregnancy complications, chronic disorders, mother with previous TLBW infant), neonatal factors (such as gestational age, ethnicity, year/season/quarter/month of birth, mode of delivery), social factors (socioeconomic status, household registration, insurance, for instance), and meteorological factors may confound the associations we observed. We were unable to control all of them in the models because some factors were not available.

## Conclusion

We performed a meta-analysis of the association between prenatal exposure to particulate matter and term low birth weight in more than 30 million singleton live births. We demonstrated that, after adjusting for confounders, maternal exposure to PM during the entire pregnancy could increase the risk of TLBW among term births, although no critical windows were identified. Stronger associations were observed in studies conducted in advanced countries, studies with low bias, and studies that adjusted for maternal age, infant sex, and parity. These findings broaden our understanding of the detrimental impact of PM_2.5_ on birth weight, underscoring the need to identify interventions targeting PM-related conditions in pregnant women. Future original study designs need to consider the impact of different exposure assessment modalities and all possible confounding factors.

## Supplementary Information

Below is the link to the electronic supplementary material.

Supplementary file1 (PDF 3573 KB)

## Data Availability

The datasets used and/or analyzed during the current study are available from the corresponding author on reasonable request.

## Abbreviations

PM: Particulate matter
LBW: Low birth weight
TLBW: Term low birth weight
EPA: Environmental Protection Agency
LUR: Land use regression
IDW: Inverse distance weighting
PRISMA: Preferred reporting items for systematic reviews and meta-analyses
OHAT: Office of Health Assessment and Translation
CI: Confidence interval
HR: Hazard ratios
OR: Odds ratios
IQR: Interquartile range
EP: Entire pregnancy
1st: The first trimester
2nd: The second trimester
3rd: The third trimester

## References

[CR1] Aguilera I, Garcia-Esteban R, Iniguez C, Nieuwenhuijsen MJ, Rodriguez A, Paez M, Ballester F, Sunyer J (2010). Prenatal exposure to traffic-related air pollution and ultrasound measures of fetal growth in the INMA Sabadell cohort. Environ Health Perspect.

[CR2] Aguilera J, Han X, Cao S, Balmes J, Lurmann F, Tyner T, Lutzker L, Noth E, Hammond SK, Sampath V, Burt T, Utz PJ, Khatri P, Aghaeepour N, Maecker H, Prunicki M, Nadeau K (2022). Increases in ambient air pollutants during pregnancy are linked to increases in methylation of IL4, IL10, and IFNgamma. Clin Epigenetics.

[CR3] Ambade B, Kumar A, Sahu LK (2021). Characterization and health risk assessment of particulate bound polycyclic aromatic hydrocarbons (PAHs) in indoor and outdoor atmosphere of Central East India. Environ Sci Pollut Res Int.

[CR4] Araban M, Kariman N, Tavafian SS, Motesaddi S, Alavimajd H, Shokravi FA (2012). Air pollution and low birth weight: a historical cohort study from Tehran. East Mediterr Health J.

[CR5] Basu R, Harris M, Sie L, Malig B, Broadwin R, Green R (2014). Effects of fine particulate matter and its constituents on low birth weight among full-term infants in California. Environ Res.

[CR6] Blencowe H, Krasevec J, de Onis M, Black RE, An X, Stevens GA, Borghi E, Hayashi C, Estevez D, Cegolon L, Shiekh S, Ponce Hardy V, Lawn JE, Cousens S (2019). National, regional, and worldwide estimates of low birthweight in 2015, with trends from 2000: a systematic analysis. Lancet Glob Health.

[CR7] Bongaerts E, Lecante LL, Bové H, Roeffaers MBJ, Ameloot M, Fowler PA, Nawrot TS (2022). Maternal exposure to ambient black carbon particles and their presence in maternal and fetal circulation and organs: an analysis of two independent population-based observational studies. Lancet Planet Health.

[CR8] Brunst KJ, Sanchez-Guerra M, Chiu YM, Wilson A, Coull BA, Kloog I, Schwartz J, Brennan KJ, BosquetEnlow M, Wright RO, Baccarelli AA, Wright RJ (2018). Prenatal particulate matter exposure and mitochondrial dysfunction at the maternal-fetal interface: effect modification by maternal lifetime trauma and child sex. Environ Int.

[CR9] Cohen AJ (2017). Estimates and 25-year trends of the global burden of disease attributable to ambient air pollution: an analysis of data from the Global Burden of Diseases Study 2015. Lancet.

[CR10] Dadvand P, Ostro B, Figueras F, Foraster M, Basagana X, Valentin A, Martinez D, Beelen R, Cirach M, Hoek G, Jerrett M, Brunekreef B, Nieuwenhuijsen MJ (2014). Residential proximity to major roads and term low birth weight: the roles of air pollution, heat, noise, and road-adjacent trees. Epidemiology.

[CR11] Daellenbach KR (2020). Sources of particulate-matter air pollution and its oxidative potential in Europe. Nature.

[CR12] Dibben C, Clemens T (2015). Place of work and residential exposure to ambient air pollution and birth outcomes in Scotland, using geographically fine pollution climate mapping estimates. Environ Res.

[CR13] Franklin M, Zeka A, Schwartz J (2007). Association between PM2.5 and all-cause and specific-cause mortality in 27 US communities. J Expo Sci Environ Epidemiol.

[CR14] GBD 2019 Risk Factors Collaborators (2020) Global burden of 87 risk factors in 204 countries and territories, 1990–2019: a systematic analysis for the Global Burden of Disease Study 2019. Lancet 396(10528):1223–1249. 10.1016/S0140-6736(20)30752-2 10.1016/S0140-6736(20)30752-2 PMC7566194 33069327

[CR15] Grevendonk L, Janssen BG, Vanpoucke C, Lefebvre W, Hoxha M, Bollati V, Nawrot TS (2016). Mitochondrial oxidative DNA damage and exposure to particulate air pollution in mother-newborn pairs. Environ Health.

[CR16] Ha S, Hu H, Roussos-Ross D, Haidong K, Roth J, Xu X (2014). The effects of air pollution on adverse birth outcomes. Environ Res.

[CR17] Ha S, Zhu Y, Liu D, Sherman S, Mendola P (2017). Ambient temperature and air quality in relation to small for gestational age and term low birthweight. Environ Res.

[CR18] Hansen CA, Barnett AG, Pritchard G (2008). The effect of ambient air pollution during early pregnancy on fetal ultrasonic measurements during mid-pregnancy. Environ Health Perspect.

[CR19] Hung TH, Chen PH, Tung TH, Hsu J, Hsu TY, Wan GH (2023). Risks of preterm birth and low birth weight and maternal exposure to NO(2)/PM(2.5) acquired by dichotomous evaluation: a systematic review and meta-analysis. Environ Sci Pollut Res Int.

[CR20] Kannan S, Misra DP, Dvonch JT, Krishnakumar A (2006). Exposures to airborne particulate matter and adverse perinatal outcomes: a biologically plausible mechanistic framework for exploring potential effect modification by nutrition. Environ Health Perspect.

[CR21] Kramer MS (1987). Determinants of low birth weight: methodological assessment and meta-analysis. Bull World Health Organ.

[CR22] Lavigne E, Burnett RT, Stieb DM, Evans GJ, Godri Pollitt KJ, Chen H, van Rijswijk D, Weichenthal S (2018). Fine particulate air pollution and adverse birth outcomes: effect modification by regional nonvolatile oxidative potential. Environ Health Perspect.

[CR23] Lee KK (2020). Adverse health effects associated with household air pollution: a systematic review, meta-analysis, and burden estimation study. Lancet Glob Health.

[CR24] Leung M, Weisskopf MG, Laden F, Coull BA, Modest AM, Hacker MR, Wylie BJ, Wei Y, Schwartz J, Papatheodorou S (2022). Exposure PM2.5 to during pregnancy and fetal growth in eastern Massachusetts, USA. Environ Health Perspect.

[CR25] Li X, Huang S, Jiao A, Yang X, Yun J, Wang Y, Xue X, Chu Y, Liu F, Liu Y, Ren M, Chen X, Li N, Lu Y, Mao Z, Tian L, Xiang H (2017). Association between ambient fine particulate matter and preterm birth or term low birth weight: an updated systematic review and meta-analysis. Environ Pollut.

[CR26] Li C, Yang M, Zhu Z, Sun S, Zhang Q, Cao J, Ding R (2020). Maternal exposure to air pollution and the risk of low birth weight: a meta-analysis of cohort studies. Environ Res.

[CR27] Li X, Cao X, Guo M, Xie M, Liu X (2020). Trends and risk factors of mortality and disability adjusted life years for chronic respiratory diseases from 1990 to 2017: systematic analysis for the Global Burden of Disease Study 2017. BMJ.

[CR28] Liang Z, Yang Y, Qian Z, Ruan Z, Chang J, Vaughn MG, Zhao Q, Lin H (2019). Ambient PM(2.5) and birth outcomes: estimating the association and attributable risk using a birth cohort study in nine Chinese cities. Environ Int.

[CR29] Lim SS (2012). A comparative risk assessment of burden of disease and injury attributable to 67 risk factors and risk factor clusters in 21 regions, 1990–2010: a systematic analysis for the Global Burden of Disease Study 2010. Lancet.

[CR30] Martens DS, Cox B, Janssen BG, Clemente DBP, Gasparrini A, Vanpoucke C, Lefebvre W, Roels HA, Plusquin M, Nawrot TS (2017). Prenatal air pollution and newborns' predisposition to accelerated biological aging. JAMA Pediatr.

[CR31] Meng QY, Turpin BJ, Lee JH, Polidori A, Weisel CP, Morandi M, Colome S, Zhang J, Stock T, Winer A (2007). How does infiltration behavior modify the composition of ambient PM2.5 in indoor spaces? An analysis of RIOPA data. Environ Sci Technol.

[CR32] Mohammed MOA, Song WW, Ma YL, Liu LY, Ma WL, Li WL, Li YF, Wang FY, Qi MY, Lv N, Wang DZ, Khan AU (2016). Distribution patterns, infiltration and health risk assessment of PM2.5-bound PAHs in indoor and outdoor air in cold zone. Chemosphere.

[CR33] Rooney AA, Boyles AL, Wolfe MS, Bucher JR, Thayer KA (2014). Systematic review and evidence integration for literature-based environmental health science assessments. Environ Health Perspect.

[CR34] Rosa MJ, Pajak A, Just AC, Sheffield PE, Kloog I, Schwartz J, Coull B, Enlow MB, Baccarelli AA, Huddleston K, Niederhuber JE, Rojo MMT, Wright RO, Gennings C, Wright RJ (2017). Prenatal exposure to PM(2.5) and birth weight: a pooled analysis from three North American longitudinal pregnancy cohort studies. Environ Int.

[CR35] Rosa MJ, Just AC, Guerra MS, Kloog I, Hsu HL, Brennan KJ, Garcia AM, Coull B, Wright RJ, Tellez Rojo MM, Baccarelli AA, Wright RO (2017). Identifying sensitive windows for prenatal particulate air pollution exposure and mitochondrial DNA content in cord blood. Environ Int.

[CR36] Smith RB, Fecht D, Gulliver J, Beevers SD, Dajnak D, Blangiardo M, Ghosh RE, Hansell AL, Kelly FJ, Anderson HR, Toledano MB (2017). Impact of London's road traffic air and noise pollution on birth weight: retrospective population based cohort study. BMJ.

[CR37] Thurston GD, Ahn J, Cromar KR, Shao Y, Reynolds HR, Jerrett M, Lim CC, Shanley R, Park Y, Hayes RB (2016). Ambient particulate matter air pollution exposure and mortality in the NIH-AARP diet and health cohort. Environ Health Perspect.

[CR38] Tian Y, Liu H, Wu Y, Si Y, Song J, Cao Y, Li M, Wu Y, Wang X, Chen L, Wei C, Gao P, Hu Y (2019). Association between ambient fine particulate pollution and hospital admissions for cause specific cardiovascular disease: time series study in 184 major Chinese cities. BMJ.

[CR39] U.S. Environmental Protection Agency (2019) Integrated science assessment (ISA) for particulate matter (final report, Dec 2019). Washington, DC. https://cfpub.epa.gov/ncea/isa/recordisplay.cfm?deid=347534#tab-3 36630543

[CR40] Viswanathan M, Ansari MT, Berkman ND, Chang S, Hartling L, McPheeters M, Santaguida PL, Shamliyan T, Singh K, Tsertsvadze A, Treadwell JR (2012) Assessing the risk of bias of individual studies in systematic reviews of health care interventions. Rockville (MD) https://www.effectivehealthcare.ahrq.gov/. PMID: 22479713. Accessed 8 Mar 2012 22479713

[CR41] WHO(2014) Comprehensive implementation plan on maternal infant and young child nutrition. Geneva https://www.who.int/publications/i/item/WHO-NMH-NHD-14.1 . Accessed 19 May 2014 10.3945/an.114.007781 PMC4288273 25593153

[CR42] Wilhelm M, Ghosh JK, Su J, Cockburn M, Jerrett M, Ritz B (2012). Traffic-related air toxics and term low birth weight in Los Angeles County, California. Environ Health Perspect.

[CR43] Wu H, Jiang B, Geng X, Zhu P, Liu Z, Cui L, Yang L (2018). Exposure to fine particulate matter during pregnancy and risk of term low birth weight in Jinan, China, 2014–2016. Int J Hyg Environ Health.

[CR44] Yuan L, Zhang Y, Gao Y, Tian Y (2019). Maternal fine particulate matter (PM(2.5)) exposure and adverse birth outcomes: an updated systematic review based on cohort studies. Environ Sci Pollut Res Int.

[CR45] Zhao Y, Wang P, Zhou Y, Xia B, Zhu Q, Ge W, Li J, Shi H, Xiao X, Zhang Y (2021). Prenatal fine particulate matter exposure, placental DNA methylation changes, and fetal growth. Environ Int.

[CR46] Zheng Y (2017). Traffic-derived particulate matter exposure and histone H3 modification: a repeated measures study. Environ Res.

