# Supplementary material for: Prenatal exposure to particulate matter and term low birth weight: systematic review and meta-analysis
Source: Environ Sci Pollut Res Int. 2023 Apr 14;30(23):63335–46. doi: 10.1007/s11356-023-26831-7 (PMC10172254; doi:10.1007/s11356-023-26831-7)

Supplementary materials

TITLE： Prenatal exposure to particulate matter and term low birth weight: systematic review and meta-analysis

Jing Liu[1,2], Yuanmei Chen[1], Die Liu[1], Fang Ye[1], Qi Sun[1], Qiang Huang[3,4], Jing Dong[2,5,6], Tao Pei[3,4], Yuan He[5,6], Qi Zhang[1]

[1] Department of Pediatrics, China-Japan Friendship Hospital, Beijing, China

[2] Graduate School of Peking Union Medical College, Chinese Academy of Medical Sciences, Beijing, China.

[3] State Key Laboratory of Resources and Environmental Information Systems, Institute of Geographical Sciences and Natural Resources Research, Chinese Academy of Sciences, Beijing, China.

[4] University of Chinese Academy of Sciences, Beijing, China.

[5] National Research Institute for Family Planning, Beijing, China.

[6] National Human Genetic Resources Center, Beijing, China.

Corresponding author：

Qi Zhang

E-mail address: zhangqikeyan@163.com

content

Supplementary Table S1. Search strategy in PubMed and Web of Science 4
Supplementary Table S2 Definitions of pregnancy outcomes in our review 6
Supplementary Table S3 Modified Office of Health Assessment and Translation (OHAT) risk-of-bias questions. 7
Supplementary Table S4a. Description of studies assessing associations between prenatal exposure to particulate matter and low birth weight among total births 9
Supplementary Table S4b. Description of studies assessing associations between prenatal exposure to particulate matter and term low birth weight among term births 10
Supplementary Table S4c. Description of studies assessing associations between prenatal exposure to particulate matter and term low birth weight among term births 14
Supplementary Table S5. Result of risk of bias apprised by OHAT in 61 eligible studies for this meta-analysis. 16
Supplementary Table S6a. Results of studies assessing associations between prenatal exposure to particulate matter and low birth weight among all births 18
Supplementary Table S6b. Results of studies assessing associations between prenatal exposure to particulate matter and term low birth weight among term births 20
Supplementary Table S6c. Results of studies assessing associations between prenatal exposure to particulate matter and term low birth weight among total births 26
Supplementary Table S7. Covariates adjusted in the original study 28
Supplementary Figure S1 Summary of adjustment for confounders in 61 eligible studies for this meta-analysis 32
Supplementary Figure S2 Meta-analysis of TLBW among term birth with exposure to PM2.5 33
Supplementary Figure S3 Meta-analysis of TLBW among all births with exposure to PM2.5 41
Supplementary Figure S4 Meta-analysis of LBW among all births with exposure to PM2.5 42
Supplementary Figure S5 Meta-analysis of TLBW among term births with exposure to PM10 43
Supplementary Figure S6 Meta-analysis of TLBW among all births with exposure to PM10 48

Supplementary Figure S7 Funnel plots..51
Supplementary Figure S8 Egger's test..53
Supplementary Figure S9 Sensitivity analysis..55

Supplementary Table S1. Search strategy in PubMed and Web of Science

NO.	Subject Headings	Entry Terms
**Particulate Matter**		
1	Particulate Matter	Ultrafine Fibers; Ultrafine Fiber; Fiber, Ultrafine; Airborne Particulate Matter; Particulate Matter, Airborne; Air Pollutants, Particulate; Particulate Air Pollutants; Ambient Particulate Matter; Particulate Matter, Ambient; Ultrafine Particulate Matter; Particulate Matter, Ultrafine; Ultrafine Particles; Particles, Ultrafine; Ultrafine Particle; Particle, Ultrafine; Fine particles; Fine particulate matter; PM; PM2.5; PM10
2	Air Pollutants	Pollutants, Air; Air Pollutant; Pollutant, Air; Air Pollutants, Environmental; Environmental Pollutants, Air; Air Environmental Pollutants; Pollutants, Air Environmental; Environmental Air Pollutants; Pollutants, Environmental Air; atmospheric pollutants
3	Air Pollution	Pollution, Air; Air Quality; Air Pollutions; air contamination; ambient air pollution; outdoor air pollution; atmospheric pollution; environmental pollution;
4	Particle Size	Particle Sizes; Size, Particle; Sizes, Particle
5	Nitrogen Dioxide	Dioxide, Nitrogen; Nitrogen Peroxide; Peroxide, Nitrogen; NO2; NOx
6	sulfur dioxide	Sulfurous Anhydride; SO2

7	ozone;	Tropospheric Ozone; Ozone, Tropospheric; Low Level Ozone; Level Ozone, Low; Ozone, Low Level; Ground Level Ozone; Level Ozone, Ground; Ozone, Ground Level; O3
8	smog	
9	Soot	Carbon Black; Black, Carbon
10	carbon monoxide	Monoxide, Carbon; CO; carbonic oxide
**Pregnancy outcomes**		
11	Infant, Low Birth Weight	Low-Birth-Weight Infant; Infant, Low-Birth-Weight; Infants, Low-Birth-Weight; Low Birth Weight Infant; Low-Birth-Weight Infants; Low Birth Weight; Birth Weight, Low; Birth Weights, Low; Low Birth Weights; LBW; BW; abnormal pregnancy; adverse pregnancy; adverse birth outcomes; birth outcomes; adverse pregnancy outcomes; small for gestational age; SGA; pregnancy outcomes; fetal outcome; Health impact assessment

Search strategy:(#1 or #2 or #3 or # 4 or # 5 or #6 or # 7 or #8 or #9 or #10) AND #11

Date of search 2022/04/07 PubMed 4389 records located.

Date of search 2022/04/07 Web of Science   13874 records located.

Supplementary Table S2 Definitions of pregnancy outcomes in our review

Outcomes	Abbreviations	Definition
Low birth weight	LBW	LBW is defined as weight at birth below 2500 g regardless of gestational age.
Term low birth weight	TLBW	≥37 weeks' gestational age, and weight at birth≥2500 g.
The First trimesters	-	1–13weeks of pregnancy
The second trimesters	-	14–26 weeks of pregnancy
The third trimester	-	commencing at week 27 and ending at birth.

Supplementary Table S3 Modified Office of Health Assessment and Translation (OHAT) risk-of-bias questions.

**Selection bias**
**Q1: Were the comparison groups appropriate?**<br>Comparison group appropriateness refers to having similar baseline characteristics between the groups aside from the exposures and outcomes under study.
**Confounding bias**
**Q2: Did the study design or analysis account for important confounding and modifying variables?**<br>Note: a parallel question under detection bias addresses reliability of the measurement of confounding variables.
**Q3: Did researchers adjust or control for other exposures that are anticipated to bias results?**
**Performance bias**
**Q4: Did researchers adhere to the study protocol?**
**Attrition/exclusion bias**
**Q5: Were outcome data complete without attrition or exclusion from analysis?**<br>Attrition rates are required to be similar and uniformly low across groups with respect to withdrawal or exclusion from analysis.
**Detection bias**
**Q6: Were the outcome assessors blinded to study group or exposure level?**<br>Blinding requires that outcome assessors do not know the study group or exposure level of the human subject or animal when the outcome was assessed.
**Q7: Were confounding variables assessed consistently across groups using valid and reliable measures?**<br>Consistent application of valid, reliable, and sensitive methods of assessing important confounding or modifying variables is required across study groups.

Note: a parallel question under selection bias addresses whether design or analysis account for confounding.
**Q8: Can we be confident in the exposure characterization?**<br>Confidence requires valid, reliable, and sensitive methods to measure exposure applied consistently across groups.
**Q9: Can we be confident in the outcome assessment?**<br>Confidence requires valid, reliable, and sensitive methods to assess the outcome and the methods should be applied consistently across groups.
**Selective reporting bias**
**Q10: Were all measured outcomes reported?**

Note: Six risk-of-bias domains were assessed for each study, and each domain was in in the form of specific questions. Each question is rated with one of four options as low, moderate, unclear (or not applicable), or high risk, rated as 1,2,3 and 4, respectively.

Studies will get a “low” tier 1 risk of bias, if they score a low risk of bias rating in most of the domains. We will use tier 3 “high” risk of bias for studies that score a high risk of bias rating in all three key domains (Selection/ Confounding/ reporting). Studies that do not receive a tier 1 or tier 3 rating will be rated as tier 2.

Supplementary Table S4a. Description of studies assessing associations between prenatal exposure to particulate matter and low birth weight among total births

**LBW of all births**																	
Study	Country	Economic status	Continent	Province	City	Study design	Study years	Setting	Total birth	Total male birth (N, %)	LBW	LBW%	Male LBW (n, %)	Exposure	Exposure window	Exposure assessment	Outcomes measured
Lin (2020)	China	developing	Asia	Beijing		RC	2014-2017	Tongzhou Maternal and Child hospital	18863	9661(51.2)	478	2.5	NA	PM2.5, PM10	EP	4	LBW, SGA
Bachwenkizi (2022)	15 African countries[1]	developing	Africa			CS	2005-2015		131594	57942(44)	13214	10	NA	PM2.5	EP	8	LBW, PTB, BW
Zou (2021)	China	developing	Asia	Shanghai		O	2006-2008		2527	1284(50.8)	75	3	NA	SO2.NO2.PM10	1st,2nd and 3rd trimester; EP	3	LBW, TLBW, PTB, SGA
Goyal (2021)	India	developing	Asia			CS	2010-2016		149416	NA	25886	17	NA	PM2.5	EP	8	LBW, BW
Yuan (2020)	China	developing	Asia	Shanghai		PC	2013-2016		3692	1891(51.2)	106	2.9	NA	PM2.5	1st,2nd and 3rd trimester; EP	2	LBW,PTB, BW
Nakhjirgan (2019)	Iran	developing	Asia	Tehran		CC	2011-2012	Three hospitals[2]	2144	1109(51.7)	468	NA	231(49.4%)	PM10, SO2, and NO2	EP	1	LBW, SGA
Chen (2018)	Australia	Advanced	Oceania	Brisbane		C	2003–2013		173720	89717(51.6)	10708	6.2	5076(47.4)	PM2.5, SO2,NO2,O3	1st,2nd and 3rd trimester; EP	3	PTB, LBW
Fleischer (2014)	22 of 24 countries in the WHOGS					C	2004-2008		192900	NA	NA	NA	NA	PM2.5	EP	8	PTB, LBW
Gray (2014)	USA	Advanced	North America	North Carolina		C	2002-2006		457642	NA	30458	6.7	NA	PM2.5,O3	EP	6	LBW, SGA, PTB,BW

Supplementary Table S4b. Description of studies assessing associations between prenatal exposure to particulate matter and term low birth weight among term births

TLBW of term birth																	
Study	Country	Economic Status	Continent	Province	City	Study design	Study years	setting	Total-term birth	Total term male birth(N,%)	TLBW (n)	TLBW (%)	Male TLBW (n, %)	Exposure	Exposure window	Exposure assessment	Outcomes measured
Guo (2020)	China	developing	Asia	Guangdong	21 cities	RC	2014-2017		2567456	1359066 (52.9)	NA	2.3	NA	PM2.5,PM10,CO,O3,NO2, SO2	1st,2nd and 3rd trimester; EP	4	TLBW, term SGA, BW
Smith (2017)	UK	Advanced	Europe	Greater London	NA	RC	2006-2010		540365	275546(51.0)	13804	2.6	5786 (41.9)	PM2.5,PM10,NO2,NOX,PM2.5,Traffic exhaust/non-exhaust	1st,2nd and 3rd trimester; EP	5	TLBW, term-SGA, term BW
Vinikoor-Imler (2014)	USA	Advanced	North America	North Carolina		RC	2003-2005		297043	151774(51.1)	6398	2.2	2575 (40.2)	PM 2.5;O3	1st,2nd and 3rd trimester;	6	TLBW, SGA,
Shang (2021)	China	developing	Asia	Shanxi	Xian	RC	2015-2018		321521	NA	4369	1.36	NA	PM2.5;PM10;CO;O3;NO2;SO2;AQI	1st,2nd and 3rd trimester; EP	3	term BW, TLBW, macrosomia
Liang (2019)	China	developing	Asia	Guangdong	Nine cites[3]	RC	2014-2017		1390998	NA	26898	1.93	10752 (40.0)	PM2.5	1st,2nd and 3rd trimester	3	PTB, TLBW
do Nascimento (2022)	Brazil	developing	South America	São Paulo municipality		CS	2011-2016		888133	452026(50.9)	31586	3.5	13080 (41.4)	PM10, NO2 and O3	1st,2nd and 3rd trimester; EP	3	TLBW, PTB
Mueller (2021)	Thailand	developing	Asia	ChonBuri,Rayong,Lampang,Phrae,Nan,Phayao,Nakhon Sawan		O	2015-2018		83931	43343(51.6)	4413	5.3	NA	PM10, NO2 and O3	1st,2nd and 3rd trimester; EP	3	TLBW, BW
Ottone (2020)	Italy	Advanced	Europe	48 of the municipalities		C	2012-2014		22379	NA	424	1.89	155 (36.6)	PM2.5	EP	1	TLBW,PTB,SGA
Ng (2017)	USA	Advanced	North America	California	4	C	2002-2009		1050330	NA	24685	2.35	NA	PM2.5	EP	3	TLBW
Wang (2019)	China	developing	Asia	Guangdong	Shenzhen	C	2005-2012		1137634	613829(54.0)	21149	1.86	8702 (41.1)	PM10, SO2, and NO2	1st,2nd and 3rd trimester; EP	3	TLBW,SGA
Kirwa (2019)	USA	Advanced	North America	Puerto Rico	37 municipalities [5]	C	1999-2013		275814	140669(51.0)	14739	5.3	6224 (42.2)	PM2.5	1st,2nd and 3rd trimester; EP	3	TLBW
Kim	Korea	Advanced	Asia			C	2010-2013		165965	NA	28728	1.7	NA	PM10	EP	3	TLBW

(2019)									9								
Li (2019)	USA	Advanced	North America	New York	Monroe County	C	2005-2016		76500	38833(50.8)	1644	2.2	659 (40.1)	PM2.5,S02,O3, BC	last 31 days, gestational month 1-9	3	TLBW
Wu (2018)	China	developing	Asia	Shandong	Jinan	CC	2014-2016	Jinan Maternity and Child Care Hospital	32446	NA	369	1.1	145 (39.3)	PM2.5 , SO2,NO2	1st,2nd and 3rd trimester; EP	4	TLBW
Lavigne (2018)	Canada	Advanced	North America	Ontario	31 cities	C	2006-2012		180793	NA	4023	2.2	1646 (40.9)	PM2.5	1st,2nd and 3rd trimester; EP, Last month	3	PTB,TLBW
Ye (2018)	China	developing	Asia	Jiangsu	Taizhou	C	2013-2016	Taizhou Maternal and Child Care Service Center	22745	12015(52.8)	823	3.6	434 (52.7)	PM2.5, PM10,NO2	1st,2nd and 3rd trimester; EP	4	PTB,TLBW
Kingsley (2017)	USA	Advanced	North America	Providence	Rhode Island	C	2002-2012	Women and Infants Hospital of Rhode Island	56633	NA	1145	2.0	NA	PM 2.5, BC	1st,2nd and 3rd trimester; EP	3,10	PTB,TLBW
Ha (2017)	USA	Advanced	North America			C	2002-2008	12 clinical sites [6]	195172	NA	4322	2.2	1716 (39.7)	PM2.5, PM10, SO2, NOX, CO, and O3	1st,2nd and 3rd trimester; EP,3-month preconception	4	SGA,TLBW
Twum (2017)	USA	Advanced	North America	Georgia	nine counties[7]	C	2014		48172	NA	1418	2.9	581 (41.0)	AQI	EP	3	TLBW
Stieb (2016)	Canada	Advanced	North America			C	1999-2008		2700620	NA	43080	1.6	17720 (41.1)	NO2	EP	1	TLBW,PTB,SGA,BW
Laurent (2016)	USA	Advanced	North America	California		C	2001-2008		3534708	NA	72632	2.1	NA	PM2.5.SO2.O3	EP	6	TLBW
Balsa (2016)	Uruguay	developing	South America	Montevideo		C	2010-2013		72920	NA	1939	2.7	NA	PM10	1st,2nd and 3rd trimester	3	TLBW,BW, PTB
Hao (2016)	USA	Advanced	North America	48 states and district of		C	2002		3271203	1664423(50.9)	81977	2.5	33947 (41.4)	PM2.5	1st,2nd and 3rd trimester; EP	6	TLBW

				Columbia)													
Dibben (2015)	UK	Advanced	Europe	Scotland		C	1994-2008		21843	NA	457	2.1	NA	PM10,SO2,NO2	EP	5	TLBW, PTB
Coker (2015)	USA	Advanced	North America	California	Los Angeles	C	1995-2006		1359284	688568(50.7)	27714	2.0	11890 (42.9)	PM2.5	EP	1	TLBW
Brown (2015)	USA	Advanced	North America	New York		C	2001-2006		431545	NA	9782	2.3	NA	PM2.5, O3	1st,2nd and 3rd trimester; EP	6	TLBW
Laurent (2014)	USA	Advanced	North America	California	Los Angeles	C	2001-2008		960945	488200(50.8)	22420	2.3	NA	PM2.5.O3.NO2	1st,2nd and 3rd trimester; EP	6	TLBW
Hyder (2014)	USA	Advanced	North America	Connecticut and Massachusetts		C	2000-2006		628131	NA	11641	1.9	NA	PM2.5	1st,2nd and 3rd trimester; EP	3	TLBW,SGA,PTB,BW at term
Harris (2014)	USA	Advanced	North America	CT, ME, MN, NJ, NY, UT, WI [8]		C	2001-2004		1374875	NA	NA	2.53	NA	PM2.5	1st,2nd and 3rd trimester; EP	6	TLBW
Cândido da Silva (2014)	Brazil	developing	South America	Mato Grosso		C	2004-2005		6147	3196(52.0)	193	3.1	NA	PM2.5,CO	1st,2nd and 3rd trimester; EP	10	TLBW
Ebisu (2012)	USA	Advanced	North America	CT, DE, MD, MA, NH, NJ, NY, PA, RI, VT, VA, WA, VA [9]	49 countries	C	2000-2007		1207800	614923(50.9)	34038	2.8	NA	PM2.5,PM10,CO,NO2,O3	EP	3	TLBW
Araban (2012)	Iran	developing	Asia	Tehran		C	2007	6 teaching hospitals [10]	225	NA	35	15.5	NA	CO,PM10,SO2, NO2,O3	1st,2nd and 3rd trimester; EP	3	TLBW
Xu (2011)	USA	Advanced	North America	Pennsylvania	Allegheny County, Pittsburgh	C	1994-2000		92447	47221(51.1)	2058	2.2	814 (39.6)	PM10	1st,2nd and 3rd trimester; EP	4	TLBW
Nascimento (2017)	Brazil	developing	South America	São José do Rio Preto (SP)		C	2012-2013		8948	4491(50.2)	301	3.4	127 (42.2)	PM10,NO2,O3	30,60 and 90-day windows before delivery	3	TLBW
Ha (2014)	USA	Advanced	North America	Florida		C	2004-2005		384637	NA	9320	2.4	3742 (40.2)	PM2.5,O3	1st,2nd and 3rd trimester;	6	TLBW, PTB,

															EP		
Dadvand (2014)	Spain	Advanced	Europe	Barcelona		C	2001-2005	obstetrics department of the Hospital clinic de Barcelona	6438	3295(51.2)	190	3.0	NA	PM2.5,PM10, NO2, NOX,	1st,2nd and 3rd trimester; EP	1	TLBW, BW, SGA
Basu (2014)	USA	Advanced	North America	California	eight counties	C	2000-2006		646296	NA	NA	2.4	NA	PM2.5 and chemical constituents	EP	3	TLBW, BW
Dugandzic (2006)	Canada	Advanced	North America	Nova Scotia	Atlee	C	1988-2000		74284	NA	1193	2.9	NA	PM10, O3, SO2	1st,2nd and 3rd trimester	3	TLBW, BW
Laurent (2013)	USA	Advanced	North America	Los Angeles and Orange		C	1997-2006	four hospitals [11]	74416	38283(51.4)	NA	2.3	NA	NO, NO2, NOX, CO,PM2.5,PM10, O3	EP	1,3,5	TLBW, BW
Bell (2010)	USA	Advanced	North America	CT, MA	[12]	C	2000-2004		76788	NA	NA	1.7	NA	PM2.5, chemical constituents	EP	3	TLBW, BW
Wilhelm (2005)	USA	Advanced	North America	California	Los Angeles	C	1994-2000		136134	70015(51.4)	2778	1.8	1188 (42.8)	PM2.5, PM10,CO	3rd	3	TLBW
Salam (2005)	USA	Advanced	North America	California		C	1975-1987		3901	1888(48.4)	72	1.3	NA	PM10,O3,NO2, CO	1st,2nd and 3rd trimester, EP	3	TLBW, BW
Lin (2004)	ROC	Advanced	Asia	Taiwan	Taipei and Kaoshiung	C	1995-1997		92288	47761(51.8)	2069	2.2	804 (38.9)	CO, SO2, O3, NO2, and PM10	1st,2nd and 3rd trimester,EP	3	TLBW
Lee (2003)	Korea	Advanced	Asia	Seoul		C	1996-1998		388105	NA	NA	2.9	NA	PM10,CO, SO2, NO2	1st,2nd and 3rd trimester,EP	3	TLBW
Chen (2002)	USA	Advanced	North America	NEVADA		C	1991-1999		36305	18465(50.9)	893	2.5	390 (43.7)	PM10, CO, and O3	3rd	3	TLBW, BW
Maisonet (2001)	USA	Advanced	North America	MA, CT, WA	[13]	C	1994-1996		89557	NA		NA	NA	PM10, CO, and SO2	1st,2nd and 3rd trimester	3	TLBW
Morello-Frosch (2010)	USA	Advanced	North America	California		C	1996-2006		3545177	NA	NA	2.3	NA	PM2.5, PM10, CO, NO2,SO2, and O3	EP	3	TLBW

Supplementary Table S4c. Description of studies assessing associations between prenatal exposure to particulate matter and term low birth weight among term births

TLBW of Total birth																	
	Country	Economic Status	Continent	Province	City	Study design	Study years	setting	Total birth(N)	Total male birth(N,%)	TLBW (n)	TLBW (%)	male TLBW (n,%)	exposure	Exposure window	Exposure assessment	Outcomes measured
Melody (2020)	Australia	Advanced	Oceania	Victoria		RC	2012-2015		285594	146615(51.3)	4730	1.7	NA	PM2.5, N02	EP	1	BW, SGA, TLBW,LGA,
Zou (2021)	China	developing	Asia	Shanghai		O	2006-2008		2527	1284(50.8)	42	1.7	NA	SO2.NO2.PM10	1st,2nd and 3rd trimester; EP	3	LBW,TLBW, PTB,SGA
Tapia (2020)	Peru	developing	South America	Lima		O	2012-2016		123034	NA	2074	1.7	NA	PM2.5	1st,2nd and 3rd trimester; EP	9	TLBW, PTB, SGA, BW
Lu (2020)	China	developing	Asia	Changsha		C	2011-2012	14	3509	1878(53.5)	45	1.3	19(42.2%)	PM10, SO2, and NO2	conception month, 1st, 2nd, and 3rd trimester, birth month, EP	4	TLBW, PLBW
Brauer (2008)	Canada	Advanced	North America	British Columbia	Vancouver	C	1999-2002		70249	36138(51.4)	894	1.3	NA	NO, NO2, PM2.5, PM10, CO, SO2, O3	EP	1,3 ,4	TLBW, PTB,SGA
Lavigne (2016)	Canada	Advanced	North America	Ontario		C	2005-2012		818400	NA	39740	4.9	19214(48.3%)	PM2.5,NO2,O3	1st,2nd and 3rd trimester; EP	4	TLBW.PTB ,SGA

Study design: C, cohort study; CC, case-control study; CS, cross-sectional study; O, observational study; PC: prospective birth cohort; RC, Retrospective cohort
Exposure assessment:1, Land use regression (LUR) model;2, AOD-based model;3, Monitoring stations; 4, Inverse distance weighting (IDW) spatial interpolation algorithm;5, dispersion modelling (KCLurban)/Airviro dispersion model/CALINE 4 dispersion model; 6,Bayesian model;8, Atmospheric Analysis Group (ACAG);9, satellite measurement and chemical transport model;10,a hybrid of land-use regression and satellite remote sensing;11, Coupled Aerosol and Trace Gas Transport Model to the Brazilian Developments of the Regional Atmospheric Modeling System:CATT-BRAMS Model

Outcomes measured: LBW, low birth weight; TLBW, term low birth weight; PTB, preterm birth; PLBW, preterm low birth weight; SGA, Small for gestational age; BW, birth weight;

[1] **Tanzania, Cameroon, Zimbabwe, Mali, Nigeria, Chad, Benin, South Africa, Burundi, Uganda, Ethiopia, Guinea, Zambia, Angola, and Malawi.** Five African countries, eight countries in the Americas, and nine Asian countries.
[2] Three hospitals affiliated with Tehran University of Medical Sciences (Imam Khomeini, Arash, and Yas)

[3] the Pearl River Delta (PRD) region. Nine cites (Guangzhou, Dongguan, Foshan, Huizhou, Jiangmen, Zhaoqing, Shenzhen, Zhuhai, and Zhongshan)
[4] Sacramento, Bakersfield, Fresno, Simi Valley, Los Angeles, Rubidoux, El Cajon, San Jose
[5] 37 municipalities with at least 50% of their land area within 10 miles of a US Environmental Protection Agency (EPA) Air Quality System (AQS) regulatory monitor.
[6] 15 hospital referral regions, 19 hospitals.
[7] nine counties: Bibb, Chatham, Clarke, Cobb, DeKalb, Fulton, Gwinnett, Muscogee, and Richmond.
[8] CT, Connecticut; ME, Maine; MN, Minnesota; NJ, New Jersey; NY, New York; UT, Utah; WI, Wisconsin.
[9] DE, Delaware; MD, Maryland; MA, Massachusetts; NH, New Hampshire; NJ, New Jersey; NY, New York, PA, Pennsylvania; RI, Rhode Island; VT, Vermont; VA, Virginia, WA, Washington, DC; VA, West Virginia;
[10] 6teaching hospitals affiliated to medical science universities in Tehran.
[11] Four hospitals (Anaheim, Long Beach, Orange Coast and Saddleback Memorial Medical Centers)
[12] New Haven/Hartford/Fairfield County of CT; Hampden of MA.
[13] Boston, Hartford, Philadelphia, Pittsburgh, Springfield
[14] A questionnaire survey on children's health in 36 kindergartens of Changsha as described in detail elsewhere.

Supplementary Table S5. Result of risk of bias apprised by OHAT in 61 eligible studies for this meta-analysis.

Study	Selection Bias Q1	Confounding Bias Q2	Confounding Bias Q3	Performance Bias Q4	Attrition Bias Q5	Detection Bias Q6	Detection Bias Q7	Detection Bias Q8	Detection Bias Q9	Reporting Bias Q10	Overall study risk of bias
Guo (2020)	1	1	1	1	1	1	1	1	1	2	1
Smith (2017)	1	1	1	1	1	1	1	1	1	1	1
Vinikoor-Imler (2014)	1	1	1	1	1	1	1	1	1	2	1
Shang (2021)	1	1	2	1	1	1	1	1	1	1	1
Liang (2019)	2	1	1	1	1	1	1	1	1	2	2
Lin (2020)	2	1	1	1	1	1	1	1	1	1	1
Melody (2020)	1	1	2	1	1	1	1	2	1	1	2
Do Nascimento (2022)	1	1	1	1	1	1	1	1	1	2	1
Bachwenkizi (2022)	1	1	1	1	1	1	1	1	2	1	1
Zou (2021)	2	1	2	1	1	1	1	1	2	2	3
Mueller (2021)	1	1	1	1	1	1	1	1	1	1	1
Goyal (2021)	2	1	2	1	1	1	1	1	2	2	3
Yuan (2020)	1	1	1	1	1	1	1	1	1	1	1
Tapia (2020)	1	1	1	1	1	1	1	1	1	1	1
Ottone (2020)	1	1	1	1	1	1	1	1	1	1	1
Lu (2020)	2	1	2	1	2	1	2	1	2	2	3
Ng (2017)	1	1	1	1	1	1	1	1	1	1	1
Wang (2019)	1	1	1	1	1	1	1	1	1	1	1
Nakhjirgan (2019)	2	1	1	1	1	1	1	1	2	1	2
Kirwa (2019)	1	1	1	1	1	1	1	1	1	1	1
Kim (2019)	1	1	1	1	1	1	1	2	1	1	1
Li (2019)	1	1	1	1	1	1	1	1	1	2	1
Wu (2018)	2	1	1	1	1	1	1	1	2	1	2
Lavigne (2018)	1	1	1	1	1	1	1	1	1	1	1
Chen (2018)	1	1	1	1	2	1	1	1	1	2	1
Ye (2018)	2	1	1	1	1	1	1	1	1	2	2
Kingsley (2017)	2	1	1	1	1	1	1	1	1	1	1
Ha (2017)	1	1	1	1	1	1	1	1	1	1	1
Twum (2017)	2	1	1	1	1	1	1	2	1	1	2
Stieb (2016)	1	1	1	1	1	1	1	1	1	1	1
Laurent (2016)	1	1	2	1	1	1	1	1	1	1	1
Balsa (2016)	1	1	1	1	1	1	1	1	1	1	1
Hao (2016)	1	1	1	1	1	1	1	1	1	1	1
Dibben (2015)	1	1	1	1	1	1	1	1	1	1	1

Coker (2015)	1	1	1	1	1	1	1	1	1	1	1
Brown (2015)	1	1	1	1	1	1	1	2	1	2	2
Laurent (2014)	1	1	1	1	1	1	1	1	1	1	1
Hyder (2014)	1	1	1	1	1	1	1	1	1	1	1
Harris (2014)	1	1	1	1	2	1	1	1	1	1	1
Fleischer (2014)	1	1	1	1	1	1	1	1	1	1	1
Cândido da Silva (2014)	1	1	1	1	1	1	1	2	1	2	2
Ebisu (2012)	1	1	1	1	1	1	1	1	1	1	1
Araban (2012)	2	1	2	1	1	1	2	1	1	3	3
Xu (2011)	1	1	1	1	2	1	1	1	1	1	1
Brauer (2008)	1	1	1	1	1	1	1	1	1	1	1
Nascimento (2017)	1	2	2	1	1	1	1	1	1	1	2
Lavigne (2016)	1	1	1	1	1	1	1	1	1	1	1
Ha (2014)	1	1	1	1	1	1	1	1	1	1	1
Gray (2014)	1	1	1	1	1	1	1	1	1	1	1
Dadvand (2014)	1	1	1	1	1	1	1	1	1	1	1
Basu (2014)	1	1	1	1	1	1	1	1	1	2	1
Dugandzic (2006)	1	1	1	1	1	1	1	1	1	1	1
Laurent (2013)	1	1	1	1	1	1	1	1	1	2	1
Bell (2010)	1	1	1	1	1	1	1	1	1	2	1
Wilhelm (2005)	1	1	1	1	1	1	1	1	1	2	1
Salam (2005)	2	1	1	1	1	1	1	1	1	1	1
Lin (2004)	2	1	1	1	1	1	1	1	1	2	2
Lee (2003)	1	1	1	1	1	1	1	1	1	2	1
Chen (2002)	1	1	1	1	1	1	1	1	1	2	2
Maisonet (2001)	1	1	1	1	1	1	1	1	1	2	1
Morello-Frosch (2010)	1	1	1	1	1	1	1	1	1	1	1

Risk of bias(1=low,2=moderate,3=high 4=unclear)

Supplementary Table S6a. Results of studies assessing associations between prenatal exposure to particulate matter and low birth weight among all births

LBW of all births					
Study	Country	Total birth(N)	LBW(n)	Study outcome	subgroup analysis
Lin (2020)	China	18863	478	A 10μg/m3 increase in PM2.5 was associated with higher risk of LBW (OR = 1.240, 95% CI: 1.019, 1.508).	
Bachwenkizi (2022)	15 African countries	131594	13214	An interquartile range (IQR)(33.9μg/m3) increase in PM2.5 during pregnancy was associated with increased odds of LBW and PTB, with odds ratios (ORs) of 1.28 (95% CI: 1.23, 1.34) and 1.08 (95% CI: 1.01, 1.16), respectively.	Region-specific analyses revealed significant associations between PM2.5 and LBW in all regions, and significant associations between PM2.5 and PTB in Western and Southern Africa. Subgroup analyses revealed that the association between PM2.5 and LBW was present in all subgroups, and stronger associations were observed in female infants, while the association between PM2.5 and PTB was larger in subgroups of older individuals living in urban areas.
Zou (2021)	China	2527	75	In the multivariate logistic regression analyses, exposures to outdoor NO2 were consistently associated with the higher odds of LBW and T-LBW. These associations were generally stronger for early months than for later months of the gestation.	This association was stronger in girls, renters, and children whose mothers≥30 years-old, with household dampness-related exposures, and with parental smoking during pregnancy
Goyal (2021)	India	149416	25886	In comparison to the reference category of in-utero PM2.5 level less than 26.7 μg m−3, the adjusted OR of LBW increases non-linearly from 1.098 (95% CI: 0.954, 1.263) for children in the exposure band 39.3–44.7 μg m−3 (i.e., the fourth octile) to 1.241 (95% CI: 1.065, 1.447) for those in the exposure band 44.7–51.6 μg m−3 (i.e., the fifth octile) and 1.405 (95% CI: 1.126,1.753) for those with in-utero PM2.5 level greater than 77.3 μg m−3 (i.e., the last octile)	
Yuan (2020)	China	3692	106	Gestational exposure to PM2.5 was associated with adverse birth outcomes in infants, and critical windows were identified as 31ste34th gestational weeks for reduced birth weight,38 the 42nd weeks for LBW and 27the30th weeks for PTB, respectively	
Nakhjirgan (2019)	Iran	2144	468	After adjustment for potential confounding variables, no statistically significant association was observed between air pollutants and LBW. The adjusted odds ratios (95% confidence interval) for PM10, SO2, and benzene were 0.999 (0.994–1.005), 0.998 (0.993–1.003), and 0.980 (0.901–1.067), respectively.	
Chen (2018)	Australia	173720	10708	Exposures to PM2.5, SO2, NO2, a n d O_3 during the whole pregnancy were associated with increased risk of LBW [IQR HRs and 95% CIs: 1.06 (1.02, 1.10), 1.12 (1.08, 1.16), 1.11 (1.03, 1.18), and 1.13 (1.09, 1.17), respectively]. Highest HRs were observed during trimester 3, and lowest in trimester 1.	For each air pollutant, stronger effects on PTB and LBW were present for exposure to low and moderate temperatures than exposure to high ambient temperature.
Fleischer (2014)	22 of 24 countries in the WHOGS	192900	NA	Across all countries, adjusting for seasonality, PM2.5 was not associated with preterm birth, but was associated with low birth weight [odds ratio (OR) = 1.22; 95% CI: 1.07, 1.39 for fourth quartile of PM2.5 (> 20.2μg/m3) compared with the first quartile (< 6.3 μg/m3)]. In China, the country with the largest PM2.5 range, preterm birth and low birth weight both were associated with the highest quartile of PM2.5 only, which suggests a possible threshold effect (OR = 2.54; CI: 1.42, 4.55 and OR = 1.99; CI: 1.06, 3.72 for	

				preterm birth and low birth weight, respectively, for PM2.5 ≥ 36.5 μg/m3 compared with PM2.5 < 12.5 μg/m3)	
Gray(2014)	USA	457642	30458	Predicted concentrations of PM2.5 and O3 were also associated with an additional effect on reductions inbirth weight and increased risks of being born low birth weight and small for gestational age.	

Supplementary Table S6b. Results of studies assessing associations between prenatal exposure to particulate matter and term low birth weight among term births

TLBW of term birth					
Study	Country	Total-term birth(N)	TLBW (n)	Study outcome	subgroup analysis
Guo (2020)	China	2567456	NA	No significant association was detected between maternal exposure to air pollutants and term LBW.	
Smith (2017)	UK	540365	13804	Interquartile range increases in NO2, NOx, PM2.5, PM10, and source specific PM2.5 from traffic exhaust (PM2.5 traffic exhaust) and traffic non-exhaust (brake or tyre wear and resuspension) (PM2.5 traffic non-exhaust) were associated with 2% to 6% increased odds of term LBW.	
Vinikoor-Imler(2014)	USA	297043	6398	In adjusted single-pollutant models for the third trimester, O3 concentration was positively associated with small for gestational age and term low birthweight births [risk ratios for an interquartile range increase in O3: 1.16 (95% CI 1.11, 1.22) for small for gestational age and 2.03 (95%CI 1.80, 2.30) for term low birthweight]; however, inverse or null associations were observed for PM2.5 [risk ratios for an interquartile range increase in PM2.5: 0.97 (95% CI 0.95, 0.99) for small for gestational age and 1.01 (95% CI 0.97, 1.06) for term low birthweight].	For term low birthweight, the associations with PM2.5 were slightly less than the null among those living in urban environments and null among those living in less urban areas. However, the opposite was observed for O3 concentrations and term low birthweight. Associations between third trimester O3 concentration and term low birthweight were observed among those living in urban environments, whereas the associations was smaller among those living in less urban environments.
Shang (2021)	China	321521	4369	During the whole pregnancy, maternal exposure to PM2.5, PM10, SO2, and CO all significantly reduced the term birth weight and increased the risk of TLBW. However, NO2 and O3 exposure have significantly increased the term birth weight, and O3 even increased the risk of macrosomia significantly. Those effects were also observed in the first and second trimesters of pregnancy. But during the third trimester, high level of air quality index (AQI) and maternal exposure to PM2.5, PM10, SO2, NO2, and CO increased the term birth weight and the risk of macrosomia, while O3 exposure was contrary to this effect.	
Liang (2019)	China	1390998	26898	We found 20% (HR = 1.20; 95% CI: 1.18, 1.22), 18% (HR = 1.18; 95% CI: 1.15, 1.20), and 20% (HR = 1.20; 95% CI: 1.17, 1.23) increases in risk of LBW, with each 10μg/m3 increase in PM2.5 from trimester 1 to trimester 3, respectively. For PTB, highest HRs were observed during trimester 3, as for LBW, stronger effect were observed during trimester 1 and trimester 3. We further estimated that 14.85% (95% CI: 13.00%, 16.61%) of the LBW cases could be attributable to PM2.5 exposure during the third trimester.	
Do Nascimento (2022)	Brazil	888133	31586	In fully adjusted models, over the entire pregnancy, a 10μg/m3 increase in O3 and PM10 was associated with increased chance of PTB (odds ratio; OR = 1.14 CI 1.13, 1.16 and 1.08 CI = 1.02, 1.15 respectively) and PM10 with TLBW (OR = 1.08 CI 1.03, 1.14).	Associations were modified by maternal educational and area-level SES for both outcomes. Mothers of lower education had an additional chance of PTB and TLBW due to PM10 exposure (OR = 1.04 CI 1.04, 1.05 and 1.10 CI 1.08, 1.14 respectively), while mothers living in low SES areas have an additional chance for TLBW (OR = 1.05 CI 1.03, 1.06).
Mueller (2021)	Thailand	83931	4413	A reduced odds ratio (OR) of LBW was associated with PM10 exposure only in trimesters	Associations with biomass burning were limited to

				one and two, with no relationship across the entire pregnancy period.	increased ORs of LBW with exposure in trimester three, but only for male births.
Ottone (2020)	Italy	22379	424	There was no association between LBW or SGA and source-specific PM2.5 components or the residual PM2.5 related to all other sources.	
Ng (2017)	USA	1050330	24685	Increased risks of TLBW associated with each interquartile range increase in exposure were 4.9% (95% confidence interval: 2.6, 7.3) for total PM2.5,	
Wang (2019)	China	1137634	21149	An interquartile range (IQR) increase in PM10 exposure during the first trimester (23.1 μg/m3) and NO2 during both the first and second trimesters (15.1 and 13.4 μg/m3) was associated with SGA and TLBW risk; odds ratios ranged from 1.01 (95% confidence interval [CI] = 1.00, 1.02) to 1.09 (1.07, 1.12).	
Kirwa (2019)	USA	275814	14739	Among term births, a SD increase in PM2.5 was associated with a 3.2% (95% CI = −1.0%, 6.3%) higher risk of LBW. First (risk ratio, 1.02; 95% CI = 1.00, 1.04) and second (1.02; 95% CI = 1.01, 1.05) trimester exposures were associated with increased LBW risk. In a 2-stage approach that longitudinally modeled monthly prenatal exposure levels, a standard deviation increase in average PM2.5 was associated with higher risk of LBW (odds ratio, 1.04; 95% CI = 1.01, 1.08).	
Kim (2019)	Korea	1659659	28728	The rate of low birth weight in term infants increased when women were exposed to PM10 > 70 μg/m3 (1.9% vs. 1.7%, P = 0.278), but this difference was not statistically significant (aOR 1.060, 95% CI: 0.953–1.178). In conclusion, PM10 exposure > 70 μg/m3 was associated with preterm births.	
Li (2019)	USA	76500	1644	Similar to our main analysis findings with term birth weight as the outcome, we did not find any increased odds of LBW associated with IQR increases in concentrations of any pollutant during any gestational month.	
Wu(2018)	China	32446	369	Term low birth weight (TLBW) increased in association with per10 μg/m3 increment in PM2.5 for the 8th month [OR = 1.13, 95% confidence interval (CI): 1.04, 1.22], the 9th month (OR = 1.06, 95% CI: 0.99, 1.15), the third trimester (OR = 1.17, 95% CI: 1.05, 1.29), and the entire pregnancy (OR = 1.38, 95% CI: 1.07, 1.77) in models adjusted for one pollutant (PM2.5).	
Lavigne (2018)	Canada	180793	4023	An interquartile increase (2:6 lg=m3) i n first-trimester PM2:5 was positively associated with term LBW among women in the highest quartile of glutathione (GSH)-related oxidative potential [odds ratio OR = 1.28; 95% confidence interval (CI): 1.10, 1.48], but not the lowest quartile (OR = 0:99; 95% CI: 0.87, 1.14; p-interaction = 0:03).	
Ye (2018)	China	22745	823	In models of adjusted single pollutant for second trimester, NO2 concentration was positively correlated with term low birth weight and preterm birth [aRR for an interquartile range increase: 1.59 (1.44, 1.75); 1.27 (1.12, 1.44)].	
Kingsley (2017)	USA	56633	1145	In adjusted models, An IQR increase(2.5 μg/m3) in modelled and monitored PM2.5 was associated with a 12.1 g (95% CI −24.2 to –0.1) and 15.9 g (95% CI −31.6 to –0.3) lower birth weight. Results for BC were highly sensitive to choice of exposure metric.	
Ha (2017)	USA	195172	4322	Air pollutant analyses were generally null but preconception elemental carbon was associated with a 4% increase in SGA while dust particles increased tLBW by 10%. Particulate matter ≤10 μm in the second trimester and whole pregnancy also appeared related to tLBW.	

Twum (2017)	USA	48172	1418	Multivariate logistic regression revealed that infants with maternal exposure to PM2.5falling within 75 to < 95th percentiles were at increased risk of LBW (OR: 1.36; 95 % CI: 1.03, 1.79), after adjusting for potential confounders.	
Stieb (2016)	Canada	2700620	43080	Associations were reduced considerably after adjustment for individual covariates and neighbourhood per cent visible minority, but remained significant for SGA (odds ratio 1.04, 95%CI 1.02–1.06 per 20 ppb NO2) and term birth weight (16.2 g reduction, 95% CI 13.6–18.8 g per 20 ppb NO2).	Associations with NO2 were of greater magnitude in a sensitivity analysis using monthly monitoring data, and among births to mothers born in Canada, and in neighbourhoods with higher incomes and a lower proportion of visible minorities.
Laurent (2016)	USA	3534708	72632	Term LBW was positively and significantly associated with interpolated measurements of ozone but not total fine PM or nitrogen dioxide. No significant association was observed between term LBW and primary PM from all sources grouped together.	
Balsa (2016)	Uruguay	72920	1939	Exposures were not consistently associated with birth weight or low birth weight among term births, though second-trimester exposures were associated with higher birth weight, contrary to expectations.	
Hao (2016)	USA	3271203	81977	Without adjusting for covariates, the odds of term LBW increased 2% [odds ratio (OR) = 1.02; 95% CI: 1.00, 1.03] for every 5-μg/m3 increase in PM2.5 exposure during the second trimester only, which remained unchanged after adjusting for county-level poverty (OR = 1.02; 95% CI: 1.01, 1.04). The odds did change to null after adjusting for individual-level predictors (OR = 1.00; 95% CI: 0.99, 1.02). Multi-level analyses, stratified by census division, revealed significant positive associations of term LBW and PM2.5 exposure (during the entire pregnancy or a specific trimester) in three census divisions of the United States: Middle Atlantic, East North Central, and West North Central, and significant negative association in the Mountain division.	
Dibben (2015)	UK	21843	457	Odds ratios of low birthweight of 1.02 (95% CI, 1.01–1.03) and 1.07 (95% CI, 1.01–1.12) with concentration increases of 1 mg/m3 for NO2 and PM10 respectively. SO2 showed no significant associations.	
Coker (2015)	USA	1359284	27714	Increased PM2.5 level was associated with higher prevalence of TLBW county-wide.	
Brown (2015)	USA	431545	9782	There were no consistent dose-response relationships between the pollutants and TLBW. Ozone exposure was associated with a higher risk of TLBW only in the first trimester, but these results were not statistically significant. Exposure to the third quartile of ozone for the full gestational period had negative associations with TLBW (odds ratio = 0.86; 95% confidence interval, 0.81-0.92)	
Laurent (2014)	USA	960945	22420	Increased LBW risks were associated with the mass of primary fine and ultrafine PM.	
Hyder (2014)	USA	628131	11641	Overall, the exposure assessment method modified the magnitude of the effect estimates of PM2.5 on birth outcomes. change in birth weight per interquartile range (2.41 μg/m3) increase in PM2.5 was −6 g (95% confidence interval = −8 to −5), −16 g (−21 to −11), and −19 g (−23 to −15), using the monitor, satellite (1), and satellite (2) methods, respectively. adjusted odds ratios, based on the same three exposure methods, for term low birth weight were 1.01 (0.98–1.04), 1.06 (0.97–1.16), and 1.08 (1.01–1.16).	
Harris (2014)	USA	1374875	NA	When we pooled the data across states, the OR for the full gestation period was 1.030 (95% CI: 1.022–1.037) and it was highest for the first trimester (OR 1.018; CI: 1.013–1.022) and decreasing during the later trimesters.	
Cândido da	Brazil	6147	193	The association between exposure to air pollutants and the risk of LBW was significant	

Silva (2014)				for the 4th quartile of PM2.5 concentrations in the 2nd trimester (OR = 1.51, 95% CI = 1.04 to 2.17) and in the 3rd trimester, and for the 4th quartile of CO concentrations in the 2nd trimester only, in adjusted analysis.	
Ebisu (2012)	USA	1207800	34038	Several PM2.5 chemical components were associated with LBW. Risk increased 4.9% (95% CI: 3.4, 6.5%), 4.7% (3.2, 6.2%), 5.7% (2.7, 8.8%), and 5.0% (3.1, 7.0%) per interquartile range increase of PM2.5 aluminum, elemental carbon, nickel, and titanium, respectively. Other PM2.5 chemical components and gaseous pollutants showed associations, but were not statistically significant in multipollutant models. The trimester associated with the highest relative risk differed among pollutants.	Effect estimates for PM2.5 elemental carbon and nickel were higher for infants of white mothers than for those of African-American mothers, and for males than females.
Araban (2012)	Iran	225	35	The results showed a significant association between exposure to CO and LBW (OR = 2.08, 95% CI: 1.70–4.60), particularly during the second trimester (OR = 3.96, CI: 1.83–12.5.)	
Xu (2011)	USA	92447	2058	The results showed that the odds ratios of term LBW per inter-quartile range increase in PM10 were 1.13.(95% CI: 1.02–1.25) during the first trimester and 1.10(95% CI: 1.00–1.22) during the second trimester after adjustment for other important covariates, respectively.	
Nascimento (2017)	Brazil	8948	301	Exposure to ozone was significantly associated with low birth weight in both sexes in the 30-day window (odds ratio, OR = 1.38) and 90-day window (OR = 1.48); and among females, in the 30-day window (OR = 1.58) and 90-day window (OR = 1.59). Exposure to particulate matter had a paradoxical protective effect.	No association was found among male newborns.
Ha (2014)	USA	384637	9320	After adjustment for potential confounders such as demographics, medical and lifestyle factors PM2.5 exposures in all trimesters were found to be significantly and positively associated with the risk of all adverse birth outcomes. Second-trimester exposure had the strongest effects. For an interquartile range (IQR) increase in PM2.5 during the second trimester, the risk of term LBW, PTB and VPTB increased by 3% [95% confidence interval (CI): 1–6%)], 12% (11–14%) and 22% (18–25%), respectively. O3 was also found to be positively associated with PTB and VPTB with the strongest effects over the whole pregnancy period [3% (1–5%) for PTD and 13% (7–19%) for VPTB for each IQR increase]. However, O3 was observed to have protective effects on term LBW. Results were consistent for multi-pollutant models.	
Dadvand (2014)	Spain	6438	190	During the whole pregnancy, maternal exposure to PM2.5, PM10, SO2, and CO all significantly reduced the term birth weight and increased the risk of TLBW. However, NO2 and O3 exposure have significantly increased the term birth weight, and O3 even increase	
Basu(2014)	USA	646296	NA	Higher full gestational exposures to PM2.5 mass and several PM2.5 constituents were significantly associated with reductions in term birth weight. The largest reductions in birth weight were associated with exposure to vanadium, sulfur, sulfate, iron, elemental carbon, titanium, manganese, bromine, ammonium, zinc, and copper. Several of these PM2.5 constituents were associated with increased risk of term LBW.	Reductions in birth weight were generally larger among younger mothers and varied by race/ethnicity.
Dugandzic (2006)	Canada	74284	1193	In the analyses unadjusted for year of birth, first trimester exposures in the highest quartile for SO2 and PM10 suggested an increased risk of delivering a LBW infant (relative risk = 1.36,95% confidence interval = 1.04 to 1.78 for SO2 exposure and relative risk = 1.33, 95% confidence interval = 1.02 to 1.74 for PM10). After adjustment for birth year, the	

				relative risks were attenuated somewhat and not statistically significant. A dose-response relationship for SO2 was noted with increasing levels of exposure. No statistically significant effects were noted for ozone.	
Laurent (2013)	USA	74416	NA	Increased risks of LBW were associated with ambient O3 concentrations as measured by monitoring stations, as well as traffic density and proximity to major roadways. LBW was not significantly associated with other air pollution metrics, except that a decreased risk was associated with ambient NO2 concentrations as measured by monitoring stations.	
Bell (2010)	USA	76788	NA	Road dust and related constituents such as silicon and aluminum were associated with lower birth weight, as were the motor-vehicle-related species such as elemental carbon and zinc, and the oil-combustion-associated elements vanadium and nickel. An interquartile range increase in exposure was associated with low birthweight for zinc (12% increase in risk), elemental carbon (13%), silicon (10%), aluminum (11%), vanadium (8%), and nickel (11%).Analysis by trimester showed effects of third-trimester exposure to elemental carbon, nickel, vanadium, and oil-combustion PM2.5.	
Wilhelm (2005)	USA	136134	2778	For women residing within a 1-mi dis-tance, we observed a 27% increase in risk for high (≥ 75th percentile) first-trimester CO exposures and preterm birth and a 36% increase for high third-trimester pregnancy CO exposures and term LBW. For particles, we observed similar size effects during early and late pregnancy for both term LBW and preterm birth. In contrast, smaller or no effects were observed beyond a 1-mi distance of a residence from a station. Associations between CO and PM10 averaged over the whole pregnancy and term LBW were generally smaller than effects for early and late pregnancy. These new results for 1994–2000 generally confirm our previous observations for the period 1989–1993, again linking CO and particle exposures to term LBW and preterm birth.	
Salam (2005)	USA	3901	72	Results from linear mixed-effects regression models showed that a 12-ppb increase in 24-hr ozone averaged over the entire pregnancy was associated with 47.2 g lower birth weight [95% confidence interval (CI), 27.4–67.0 g], and this association was most robust for exposures during the second and third trimesters. A 1.4-ppm difference in first-trimester carbon monoxide exposure was associated with 21.7 g lower birth weight (95% CI,1.1–42.3 g) and 20% increased risk of intrauterine growth retardation (95% CI, 1.0–1.4). First-trimester CO and third-trimester O3 exposures were associated with 20% increased risk of intrauterine growth retardation. A 20-µg/m3 difference in levels of particulate matter ≤ 10 µm in aerodynamic diameter (PM10) during the third trimester was associated with a 21.7-g lower birth weight (95% CI, 1.1–42.2 g), but this association was reduced and not significant after adjusting for O3.	
Lin (2004)	ROC	92288	2069	This study suggested a 26% increase in term LBW risk given maternal ambient exposure to SO2 concentration exceeding 11.4 ppb during pregnancy compared to low exposure (o7.1 ppb) (OR=1.26, 95% CI=1.04–1.53). Since the relative risk of term LBW was reassessed according to exposure level in each trimester, mothers exposed to 412.4 ppb of SO2 in the last trimester showed 20% higher risk (OR=1.20, 95% CI=1.01–1.41) of term LBW delivery than mothers with lower exposure (o6.8 ppb). No significant elevation ORs was observed for other air pollutants.	
Lee (2003)	Korea	388105	NA	The monthly analyses suggested that the risks for LBW tended to increase with carbon monoxide (CO) exposure between months 2–5 of pregnancy, with exposure to particles <10 mm (PM10) in months 2 and 4, and for sulphur dioxide (SO2) and nitrogen dioxide	

				(NO_2) exposure between months 3–5.	
Chen (2002)	USA	36305	893	PM10 was not found to be related with the risk of LBW from logistic regression.CO and O3 were not found to be associated with birth weight or risk of LBW of newborns by the same modeling procedure.	
Maisonet (2001)	USA	89557		Our results suggest that exposures to ambient CO and SO2 increase the risk for term LBW. This risk increased by a unit increase in CO third trimester average concentration [adjusted odds ratio (AOR) 1.31; 95% confidence interval (CI)1.06,1.62]. Infants with SO2 second trimester exposures falling within the 25 and < 50th (AOR 1.21;CI 1.07,1.37), the 50 to < 75th (AOR 1.20; CI 1.08,1.35), and the 75 to < 95th (AOR 1.21; CI 1.03,1.43) percentiles were also at increased risk for term LBW when compared to those in the reference category (< 25th percentile). There was no indication of a positive association between prenatal exposures to PM10 and term LBW.	
Morello-Frosch (2010)	USA	3545177	NA	In multivariate models, pollutants were associated with decreased birth weight; -5.4 grams (95% confidence interval -6.8 g, -4.1 g) per ppm carbon monoxide, -9.0 g (-9.6 g, -8.4 g) per pphm nitrogen dioxide, -5.7 g (-6.6 g, -4.9 g) per pphm ozone, -7.7 g (-7.9 g, -6.6 g) per 10 µg/m3 particulate matter under 10 µm, -12.8 g (-14.3 g, -11.3 g) per 10 µg/m3 particulate matter under 2.5 µm, and -9.3 g (-10.7 g,-7.9 g) per 10 µg/m3 of coarse particulate matter. With the exception of carbon monoxide, estimates were largely unchanged after controlling for co-pollutants. Effect estimates for the third trimester largely reflect the results seen from full pregnancy exposure estimates; greater variation in results is seen in effect estimates specific to the first and second trimesters.	

Supplementary Table S6c. Results of studies assessing associations between prenatal exposure to particulate matter and term low birth weight among total births

**TLBW of Total birth**					
Study	country	Total birth(N)	TLBW (n)	Study outcome	subgroup analysis
Melody (2020)	Australia	285594	4730	IQR increases in ambient NO2 and PM2.5 were associated with fetal growth restriction, including decrements in birth weight (NO2β−22.8 g; 95%CI -26.0,−19.7; PM2.5β−14.8 g; 95%CI -17.4,−12.2) and increased risk of SGA (NO2 RR 1.08; 95%CI 1.06, 1.10; PM2.5 RR 1.05; 95%CI 1.04, 1.07) and tLBW (NO2 RR 1.06; 95%CI 1.01, 1.10; PM2.5 RR 1.04; 95%CI 1.03, 1.08).	NA
Zou (2021)	China	2527	42	In the multivariate logistic regression analyses, exposures to outdoor NO2 were consistently associated with the higher odds of LBW and T-LBW. These associations were generally stronger for early months than for later months of the gestation.	This association was stronger in girls, renters, and children whose mothers≥30 years-old, with household dampness-related exposures, and with parental smoking during pregnancy.
Tapia (2020)	Peru	123034	2074	PM2.5 exposure was positively associated with low birth weight at term (TLBW) during entire pregnancy (OR: 1.11; 95% CI: 1.03–1.20), and at the first (OR: 1.11; 95% CI: 1.03–1.20), second (OR: 1.09; 95% CI: 1.01–1.17), and third trimester (OR: 1.10; 95% CI: 1.02–1.18) per IQR (9.2 µg/m3) increase.	
Lu (2020)	China	3509	45	Term LBW (TLBW) was significantly associated with exposure to ambient PM10 during pregnancy, with OR (95% CI)=1.47 (1.00-2.14) for per IQR increase after adjustment for the covariates and home environmental factors. Specifically, we identified the significant association in early phase of pregnancy including conception month (1.90, 1.09-3.30) and the first trimester (1.72, 1.10-2.69).	We further found that TLBW was significantly related with parental smoking at home, OR (95% CI) = 2.17 (1.09-4.33).
Brauer (2008)	Canada	70249	894	Residence within 50 m of highways was associated with a 26% increase in SGA [95% confidence interval (CI), 1.07–1.49] and an 11% (95% CI, 1.01–1.23) increase in LBW. Exposure to all air pollutants except O3 was associated with SGA, with similar odds ratios (ORs) for LUR and monitoring estimates (e.g., LUR: OR = 1.02; 95% CI, 1.00–1.04; IDW: OR = 1.05; 95% CI, 1.03–1.08 per 10-µg/m3 increase in NO). For preterm births, associations were observed with PM2.5 for births < 37 weeks gestation (and for other pollutants at < 30 weeks). No consistent patterns suggested exposure windows of greater relevance.	
Lavigne (2016)	Canada	818400	39740	We did not find statistically significant effect modification for the other outcomes investigate.	

Supplementary Table S7. Covariates adjusted in the original study

Study	A	B	C	D	E	F	G	H	I	G	K	L	M	N	O	P	Q	R	S	T	U	V	W	X	Y
Guo (2020)	+				+		+		+				+			+	+			+			+		
Smith (2017)	+				+		+		+									+	+			+			
Vinikoor-Imler (2014)	+				+	+		+					+			+		+					+		
Shang (2021)	+				+		+																		
Liang (2019)	+								+							+				+					
Lin (2020)	+	+			+		+		+							+			+	+			+		
Melody (2020)	+												+			+			+	+			+		
do Nascimento (2022)	+	+				+			+								+		+	+					
Bachwenkizi (2022)	+	+						+	+				+						+	+					
Zou (2021)	+								+				+												
Mueller (2021)	+						+		+							+			+	+		+			
Goyal (2021)																			+			+			
Yuan (2020)	+	+					+		+			+			+	+				+	+	+			
Tapia (2020)	+					+	+			+						+									
Ottone	+	+							+				+			+									

(2020)																									
Lu (2020)	+								+										+			+			
Ng (2017)	+	+			+		+												+	+		+			
Wang (2019)	+	+				+	+			+						+					+		+		
Nakhjirgan (2019)	+						+		+							+									
Kirwa (2019)	+	+				+	+	+	+							+		+	+			+			
Kim (2019)	+	+						+								+			+			+			
Li (2019)		+	+		+	+	+		+				+			+				+	+	+	+		+
Wu (2018)		+	+			+			+	+						+							+		
Lavigne (2018)	+		+				+		+	+			+			+	+	+	+	+		+			
Chen (2018)	+								+				+			+									
Ye (2018)	+	+							+							+						+	+		
Kingsley (2017)	+	+			+		+						+			+		+	+					+	
Ha (2017)	+		+		+				+	+		+	+			+		+		+	+		+	+	
Twum (2017)	+	+			+	+			+			+	+					+							
Stieb (2016)	+	+					+	+	+							+	+	+	+			+			
Laurent (2016)	+	+			+														+						
Balsa (2016)	+	+				+	+						+					+		+					
Hao (2016)	+	+			+	+	+		+							+		+	+			+			
Dibben	+	+			+		+						+			+		+	+			+			

(2015)																									
Coker (2015)	+	+			+		+		+							+									
Brown (2015)	+	+			+	+			+	+			+			+									
Laurent (2014)	+	+			+	+	+		+							+			+						
Hyder (2014)	+	+			+	+	+		+		+		+			+	+	+					+		
Harris (2014)	+	+			+	+			+	+			+					+	+						
Fleischer (2014)	+	+				+			+							+			+						
Cândido da Silva (2014)	+	+				+			+								+								
Ebisu (2012)	+	+			+	+	+		+			+	+			+	+	+	+			+			
Araban (2012)																									
Xu (2011)	+	+			+	+	+		+		+		+	+		+						+			
Brauer (2008)	+	+							+				+			+			+			+			
Nascimento (2017)																									
Lavigne (2016)	+	+						+	+				+			+	±		+			+			
Ha (2014)	+	+			+	+			+			+	+					+	+				+		
Gray (2014)	+					+	+		+				+			+		+				+			
Dadvand (2014)	+	+	+		+				+			+	+			+		+	+		+		+		

Basu (2014)	+	+			+		+		+									+	+		+			
Dugandzic (2006)	+						+		+		+		+	+		+			+			+			
Laurent (2013)	+				+		+		+							+			+					+	
Bell (2010)	+	+			+	+	+		+			+	+			+	+			+		+			
Wilhelm (2005)	+	+		+	+	+	+		+		+					+						+			
Salam (2005)	+			+	+		+		+	+			+			+		+	+			+			
Lin (2004)	+	+					+		+							+						+			
Lee (2003)	+	+					+		+							+						+			
Chen (2002)	+	+			+	+	+		+			+	+	+					+						+
Maisonet (2001)	+	+			+	+	+		+			+	+	+		+		+				+			
Morello-Frosch (2010)	+	+			+	+	+		+							+		+	+			+			

A, maternal age, maternal education, Anemia/hypertension/ diabetes pre-pregnancy ; D, Interval since previous live birth; E, Ethnicity; F, Prenatal care/examination/the trimester begins prenatal visits; G, Gestational age; H, Household registration/ rural-urban continuum codes category; I, Infant sex; J, Gestational diabetes/ hypertension /preeclampsia; K, Mother with previous SGA/ LBW infant; L, Maternal alcohol use after conception ; M, Maternal smoking status (after conception/ during pregnancy); N, Maternal weight change during pregnancy; O, Passive smoking ; P, Parity/ gravidity/number of birth /previous pregnancy history/birth order; Q, Mode of delivery/previous caesarean section delivery; R, marital status (married, single); S, Socioeconomic status (SES)/income/employment/Region/city; T, Meteorological factors; U, Body mass index before pregnancy; V, year/season/quarter/month of birth; W, year/season/quarter/month of conception; X, Insurance; Y, Drug use

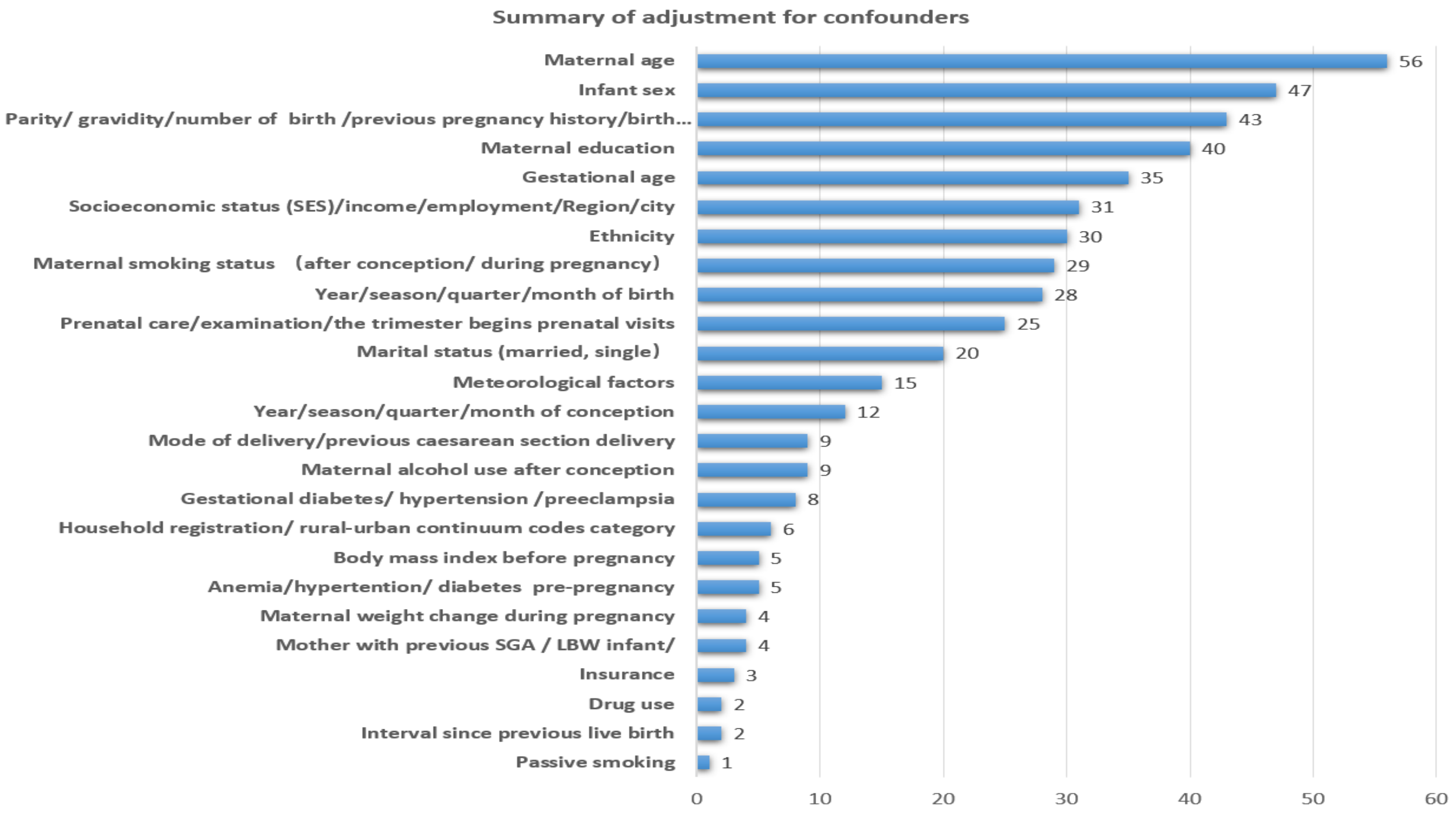

Supplementary Figure S1 Summary of adjustment for confounders in 61 eligible studies for this meta-analysis

Supplementary Figure S2 Meta-analysis of TLBW among term birth with exposure to PM2.5

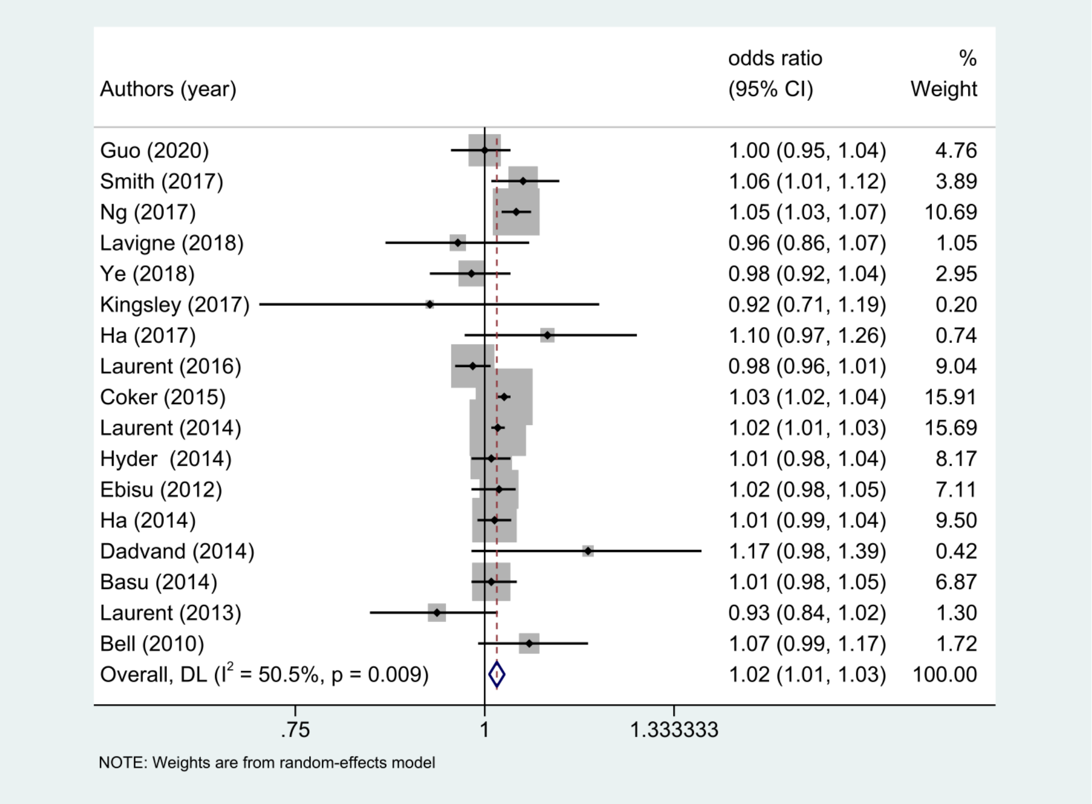

Supplementary Figure S2-a Meta-analysis of TLBW among term birth with exposure to PM2.5 for per IQR increase in EP

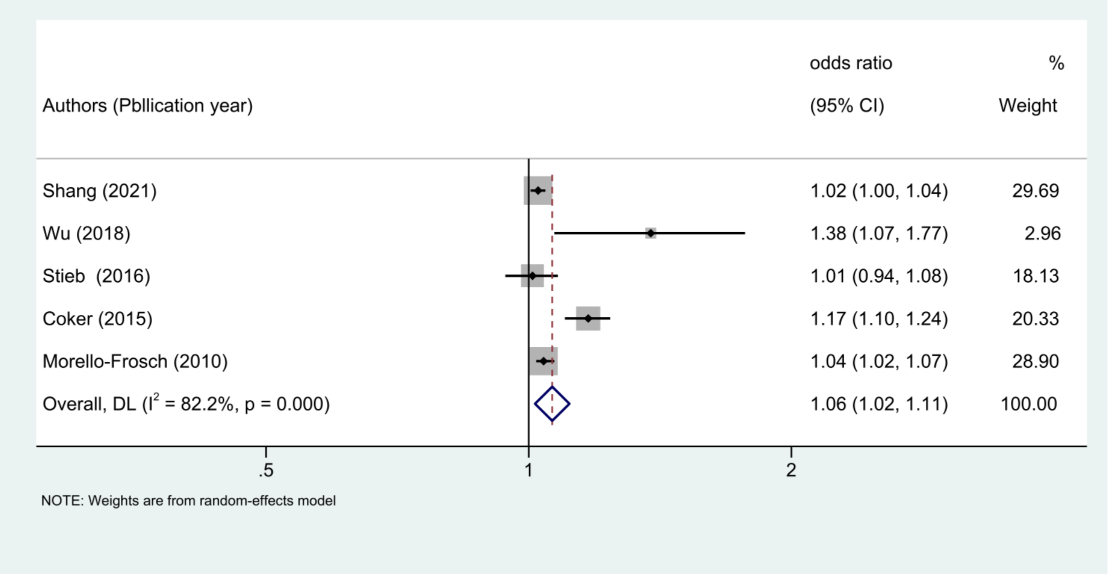

Supplementary Figure S2-b Meta-analysis of TLBW among term birth with exposure to PM2.5 for per 10μg/m3 increase in EP

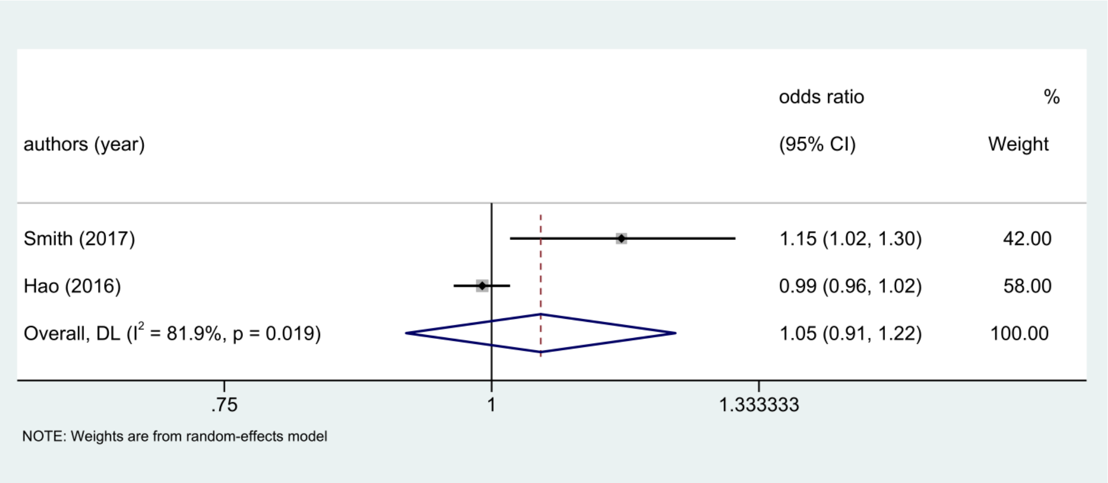

Supplementary Figure S2-c Meta-analysis of TLBW among term birth with exposure to PM2.5 for per 5μg/m3 increase in EP

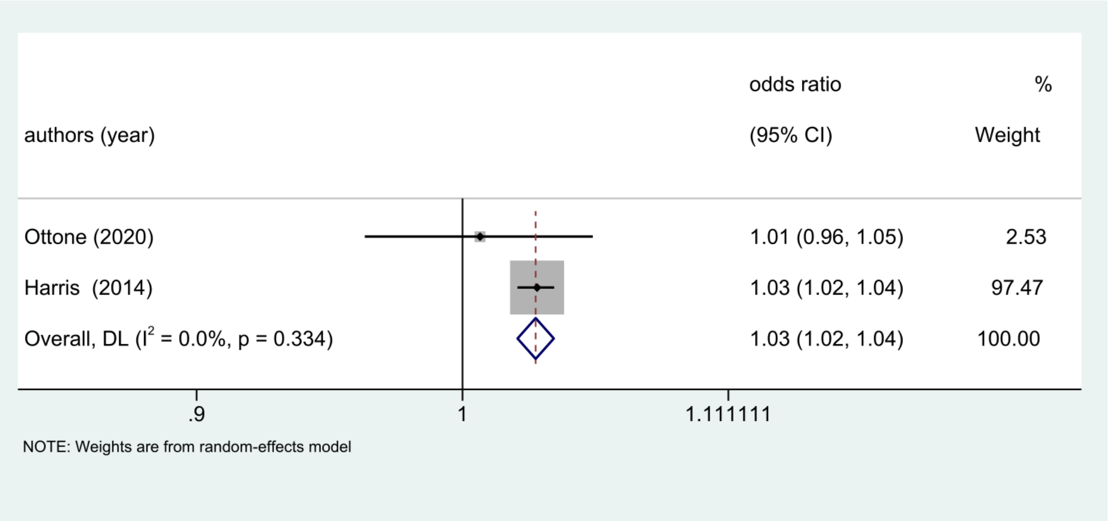

Supplementary Figure S2-d Meta-analysis of TLBW among term birth with exposure to PM2.5 for per 1μg/m3 increase in EP

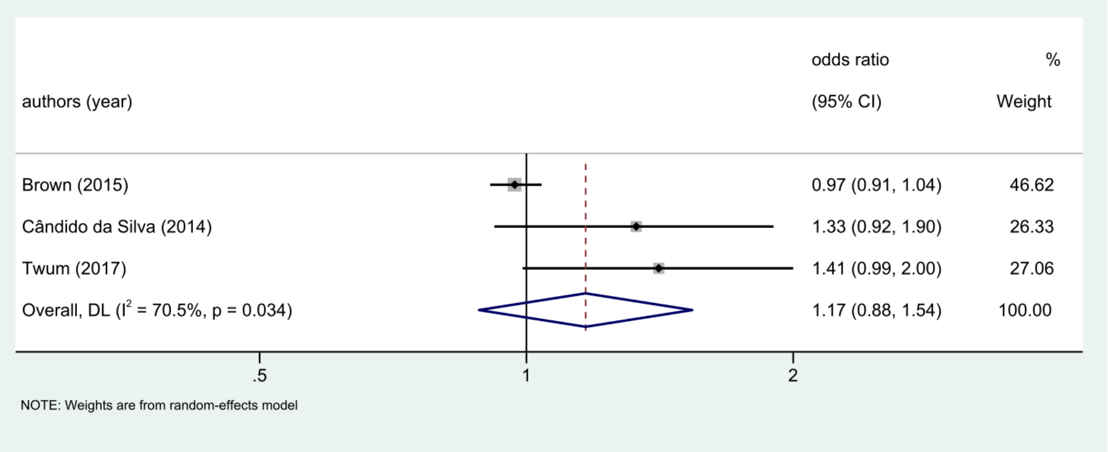

Supplementary Figure S2-e Meta-analysis of TLBW among term birth with high versus low exposure to PM2.5 in EP

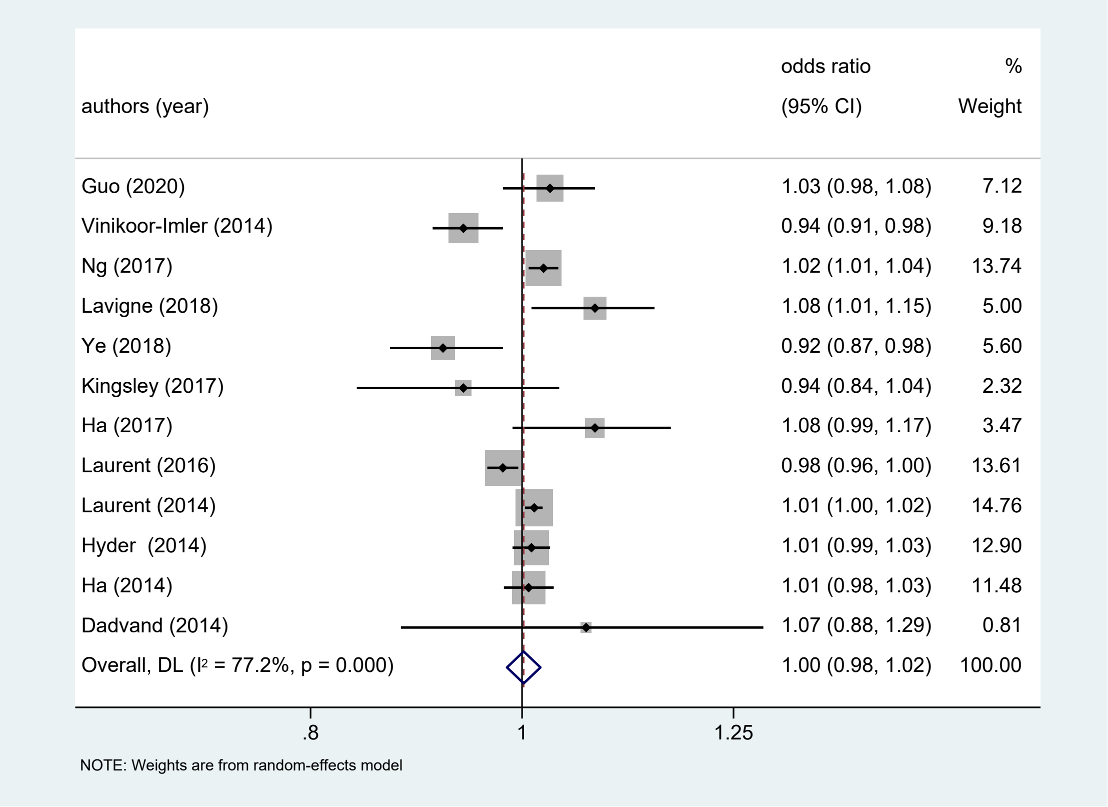

Figure S2-f Meta-analysis of TLBW among term birth with exposure to PM2.5 for per IQR increase in 1st

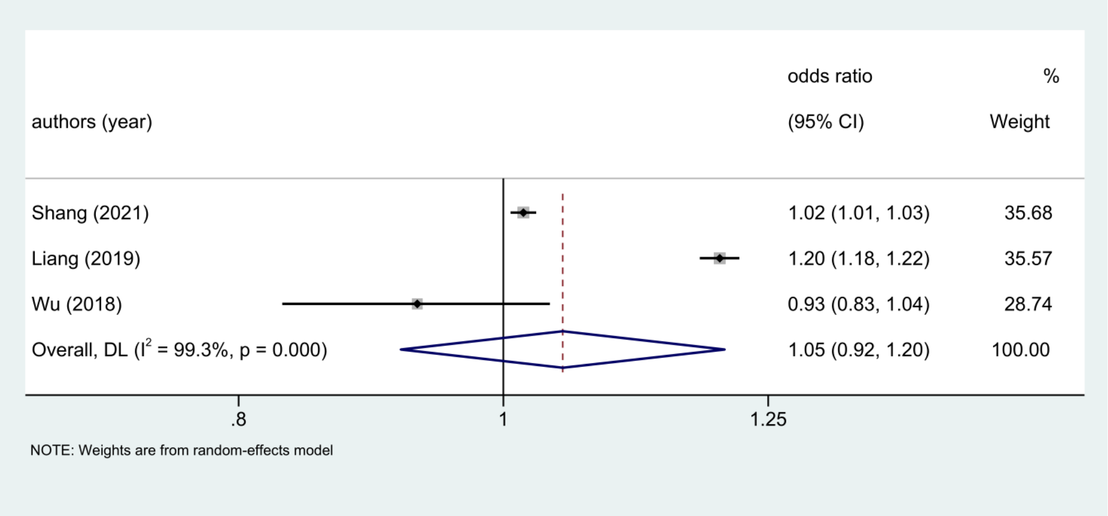

Figure S2-g Meta-analysis of TLBW among term birth with exposure to PM2.5 for per 10μg/m3 increase in 1st

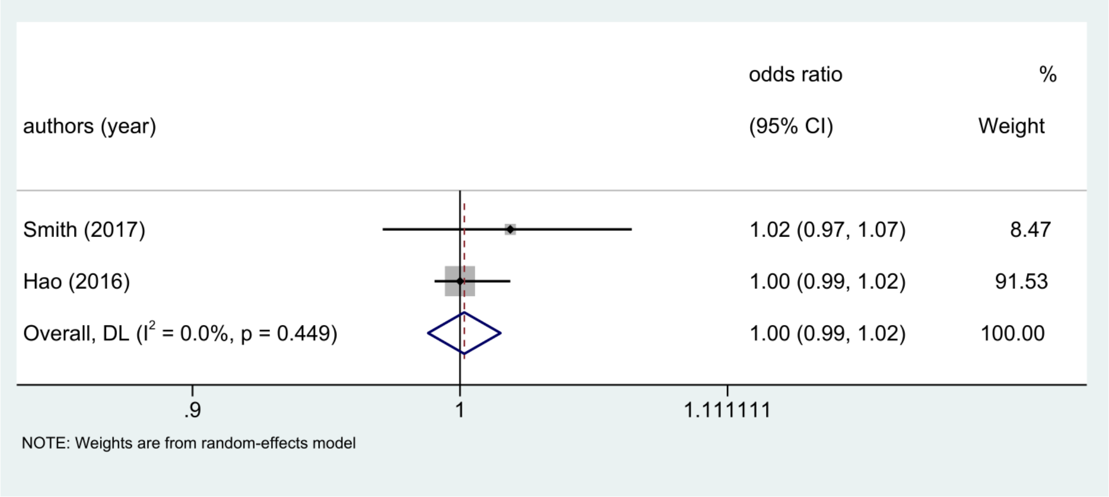

Figure S2-h Meta-analysis of TLBW among term birth with exposure to PM2.5 for per 5μg/m3 increase in 1st

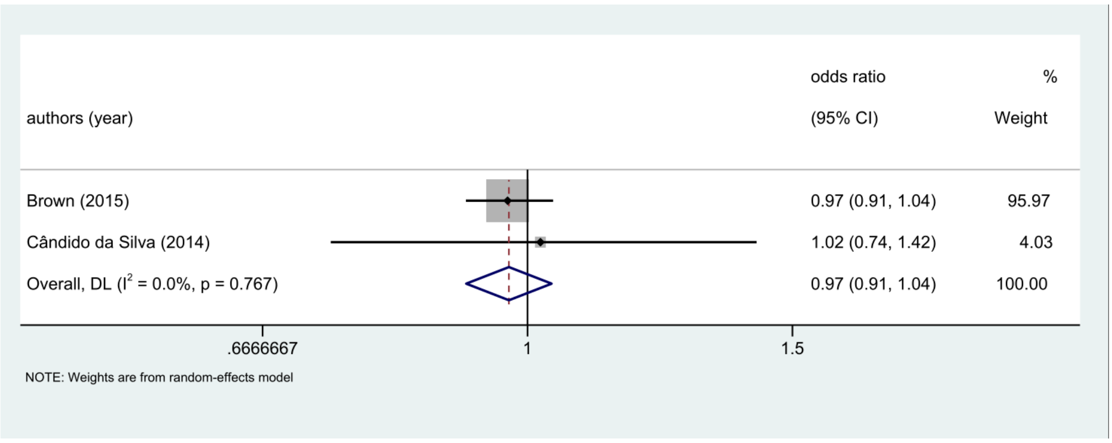

Figure S2-i Meta-analysis of TLBW among term birth with high versus low exposure to PM2.5 in 1st

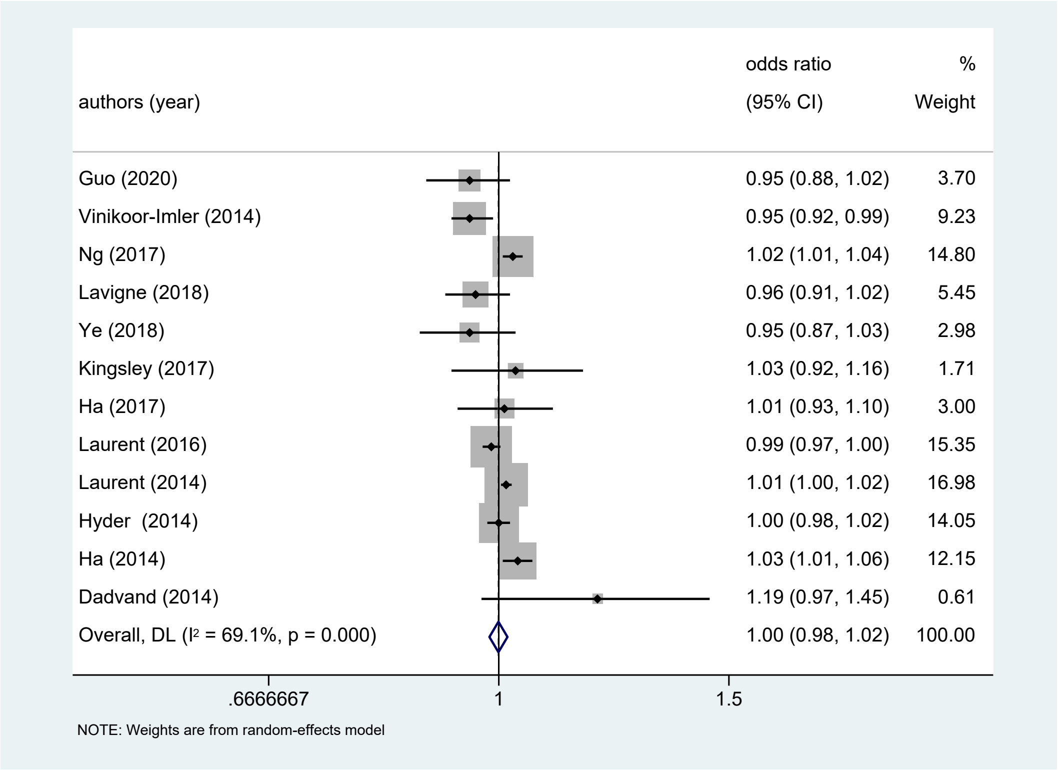

Figure S2-j Meta-analysis of TLBW among term birth with exposure to PM2.5 for per IQR increase in 2nd

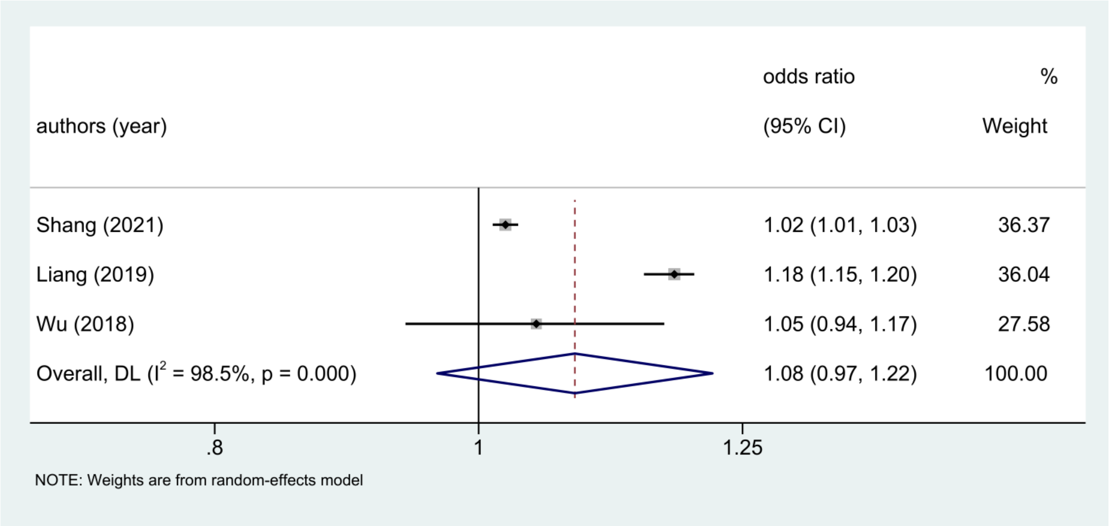

Figure S2-k Meta-analysis of TLBW among term birth with exposure to PM2.5 for per 10μg/m3 increase in 2nd

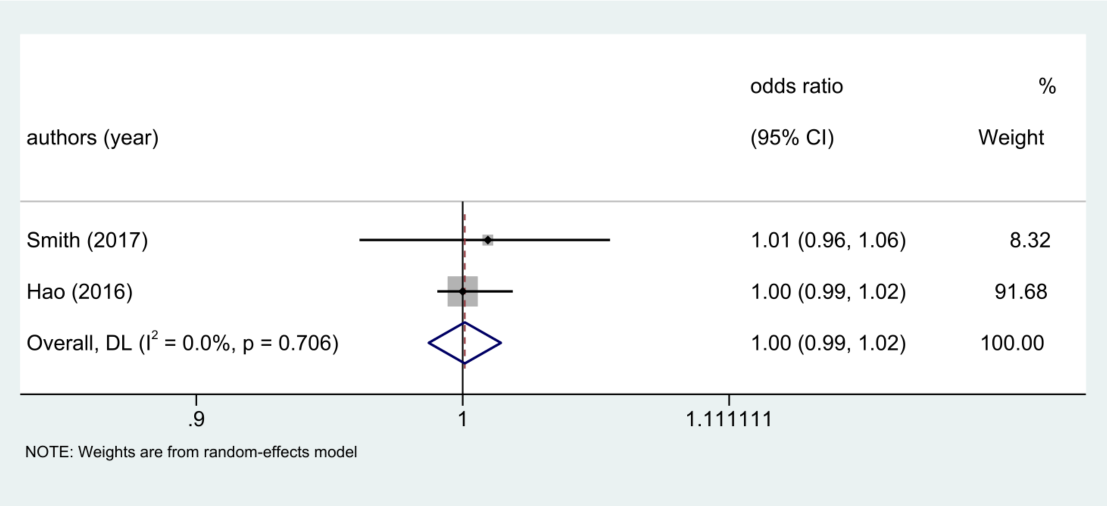

Figure S2-l Meta-analysis of TLBW among term birth with exposure to PM2.5 for per 5μg/m3 increase in 2nd

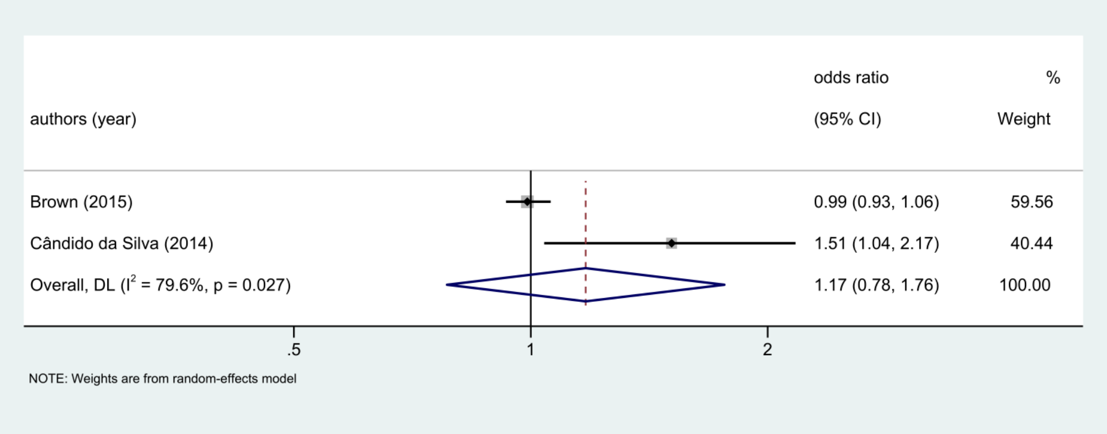

Figure S2-m Meta-analysis of TLBW among term birth with high versus low exposure to PM2.5 in 2nd

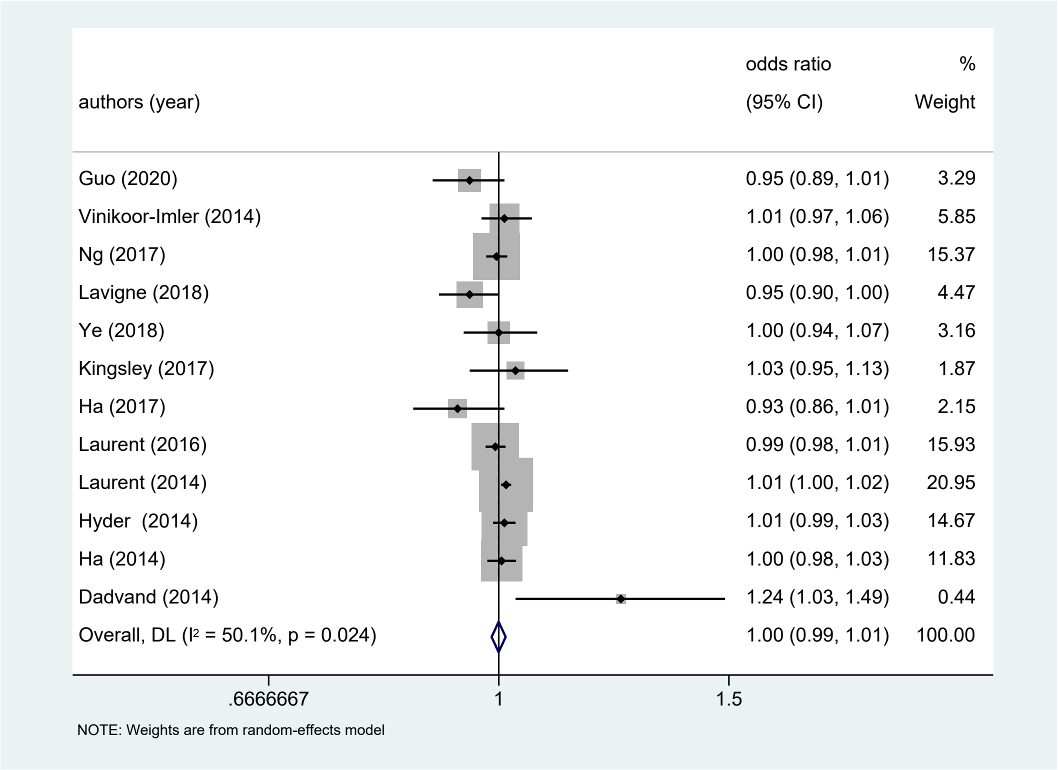

Figure S2-n Meta-analysis of TLBW among term birth with exposure to PM2.5 for per IQR increase in 3rd

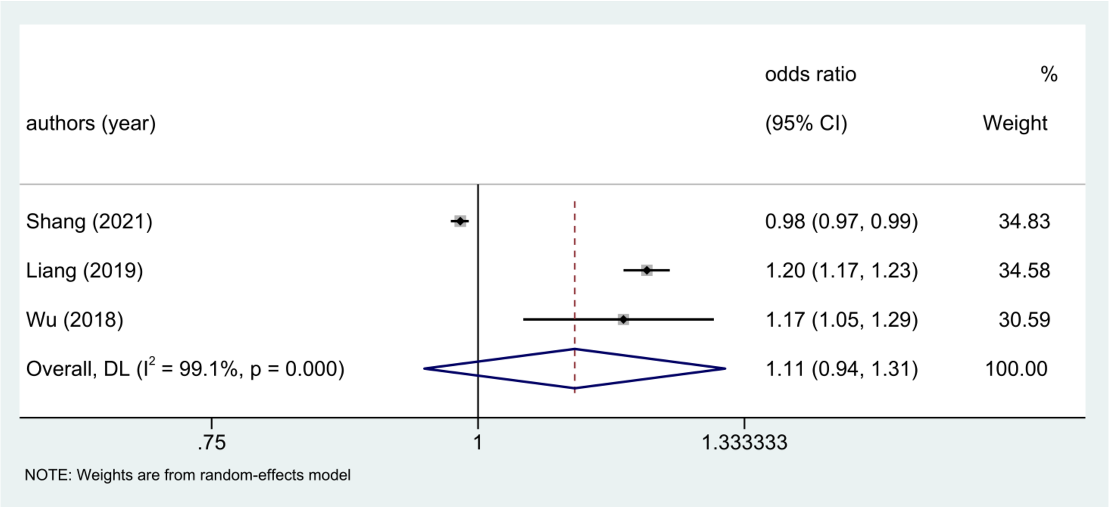

Figure S2-o Meta-analysis of TLBW among term birth with exposure to PM2.5 for per 10μg/m3 increase in 3rd

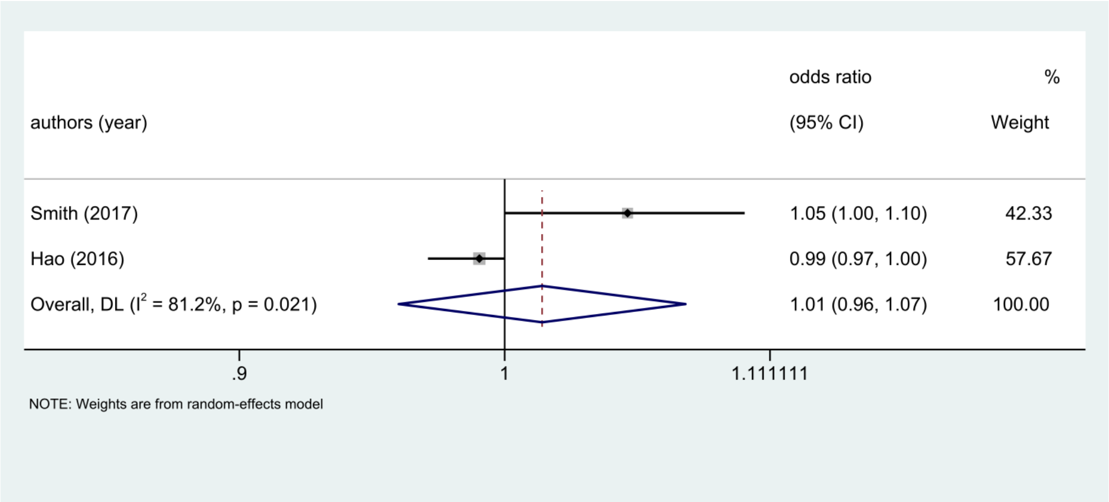

Figure S2-p Meta-analysis of TLBW among term birth with exposure to PM2.5 for per 5μg/m3 increase in 3rd

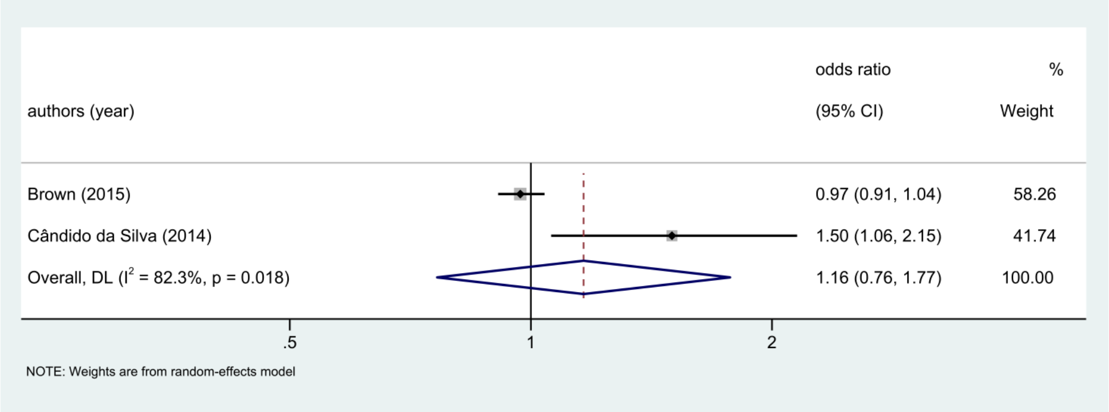

Figure S2-q Meta-analysis of TLBW among term birth with high versus low exposure to PM2.5 in 3rd

Supplementary Figure S3 Meta-analysis of TLBW among all births with exposure to PM2.5

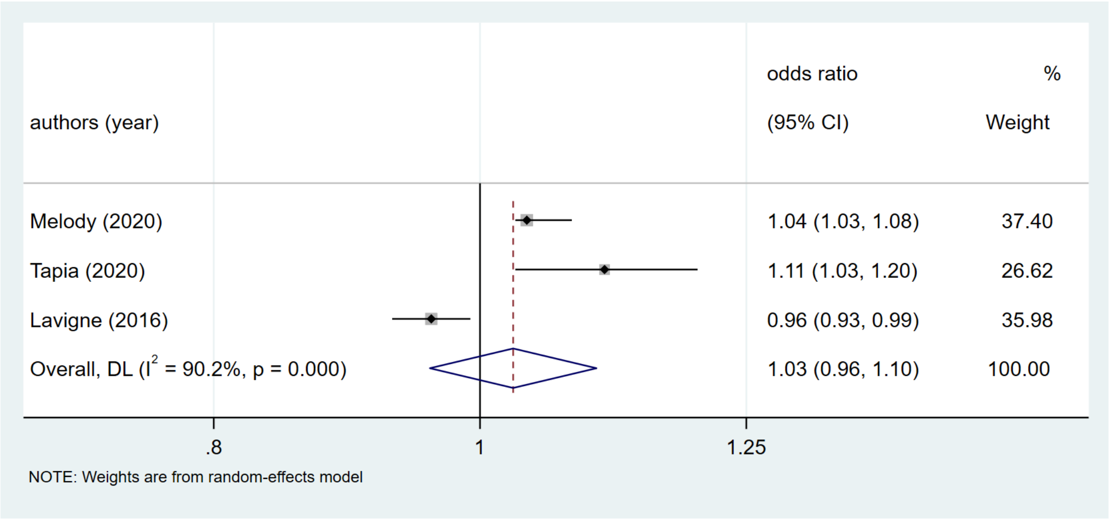

Supplementary Figure S3-a Meta-analysis of TLBW among all births with exposure to PM2.5 for per IQR increase in EP

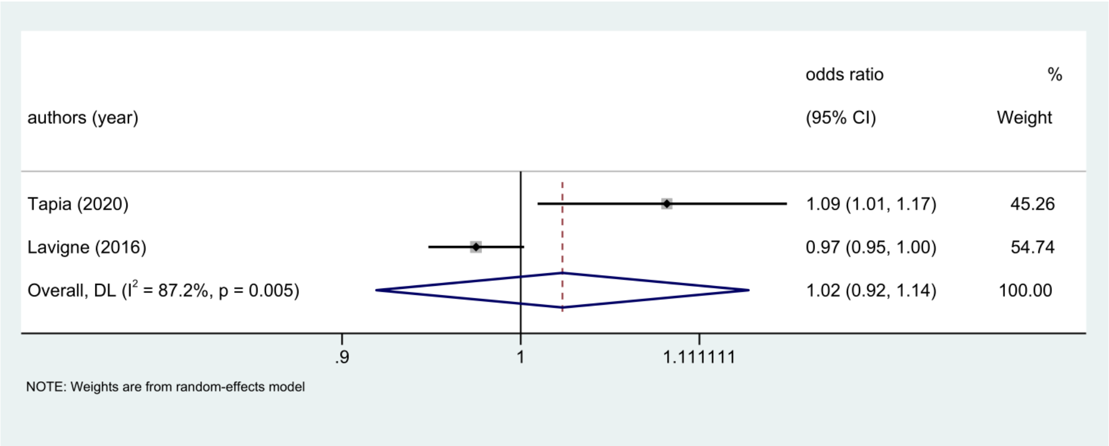

Supplementary Figure S3-b Meta-analysis of TLBW among all births with exposure to PM2.5 for per IQR increase in 2nd

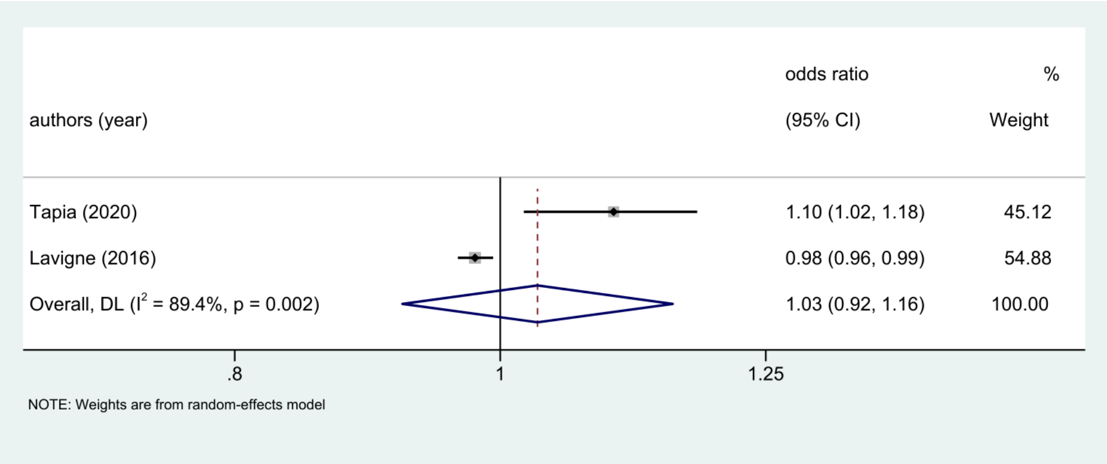

Supplementary Figure S3-c Meta-analysis of TLBW among all births with exposure to PM2.5 for per IQR increase in 3rd

Supplementary Figure S4 Meta-analysis of LBW among all births with exposure to PM2.5

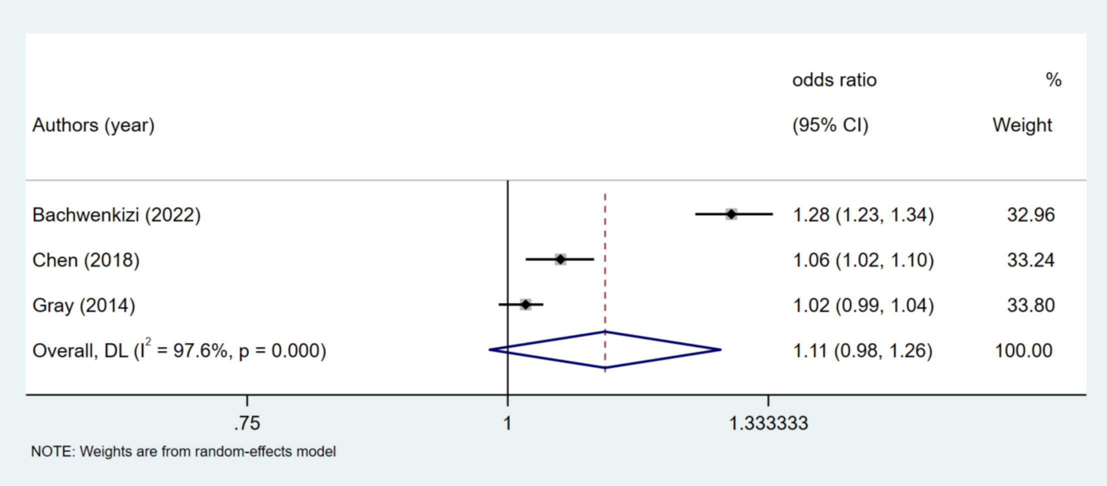

Supplementary Figure S4-a Meta-analysis of LBW among all births with exposure to PM2.5 for per IQR increase in EP

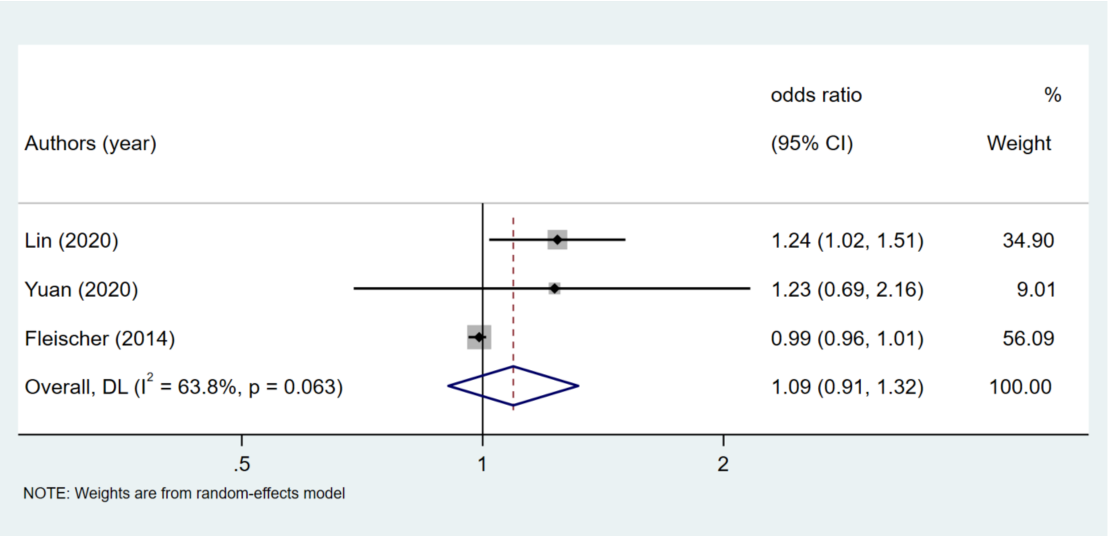

Supplementary Figure S4-b Meta-analysis of LBW among all births with exposure to PM2.5 for per 10μg/m3 increase in EP

Supplementary Figure S5 Meta-analysis of TLBW among term births with exposure to PM10

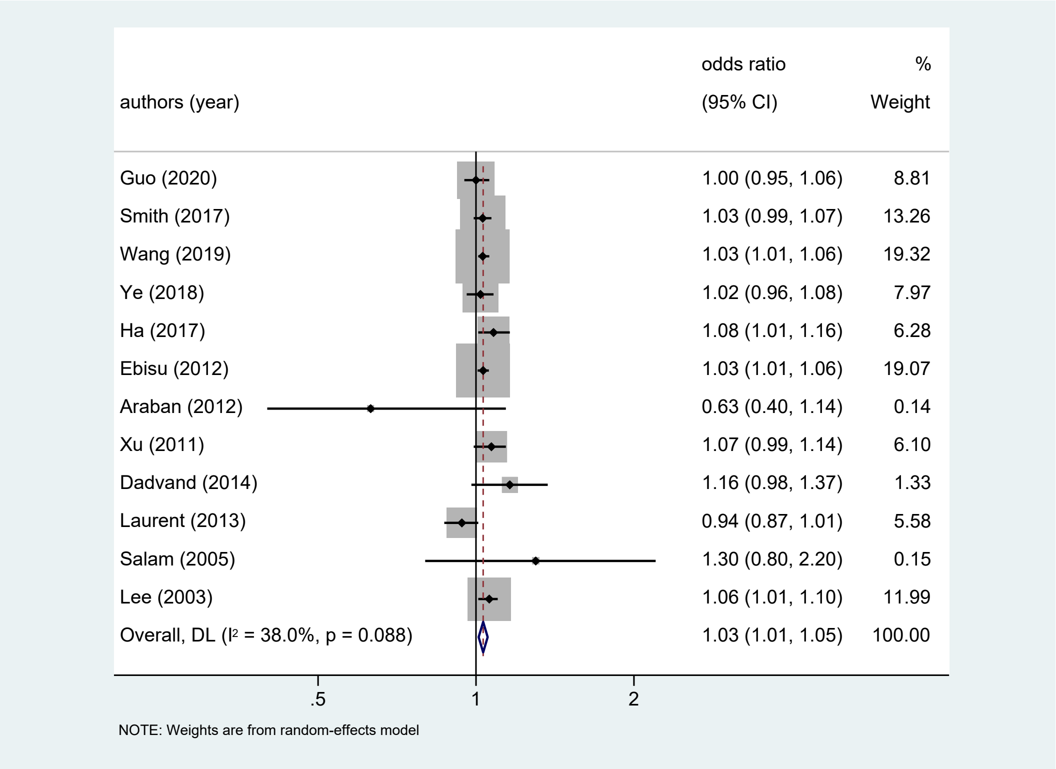

Supplementary Figure S5-a Meta-analysis of TLBW among term births with exposure to PM10 for per IQR increase in EP

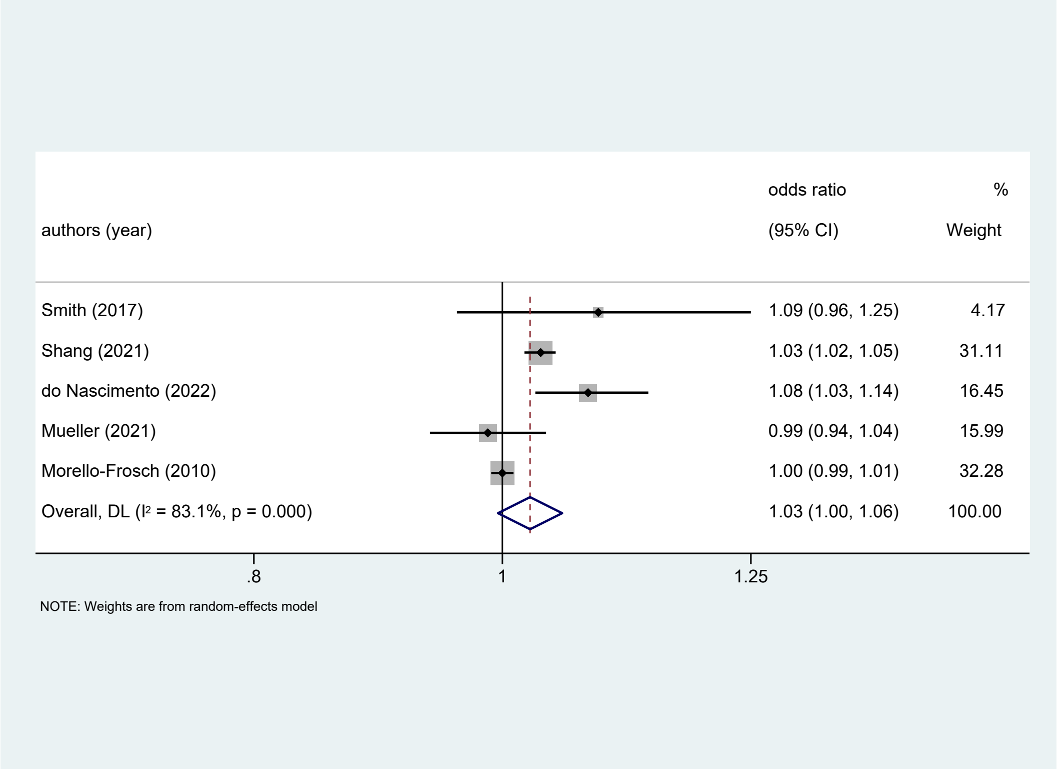

Supplementary Figure S5-b Meta-analysis of TLBW among term births with exposure to PM10 for per 10μg/m3 increase in EP

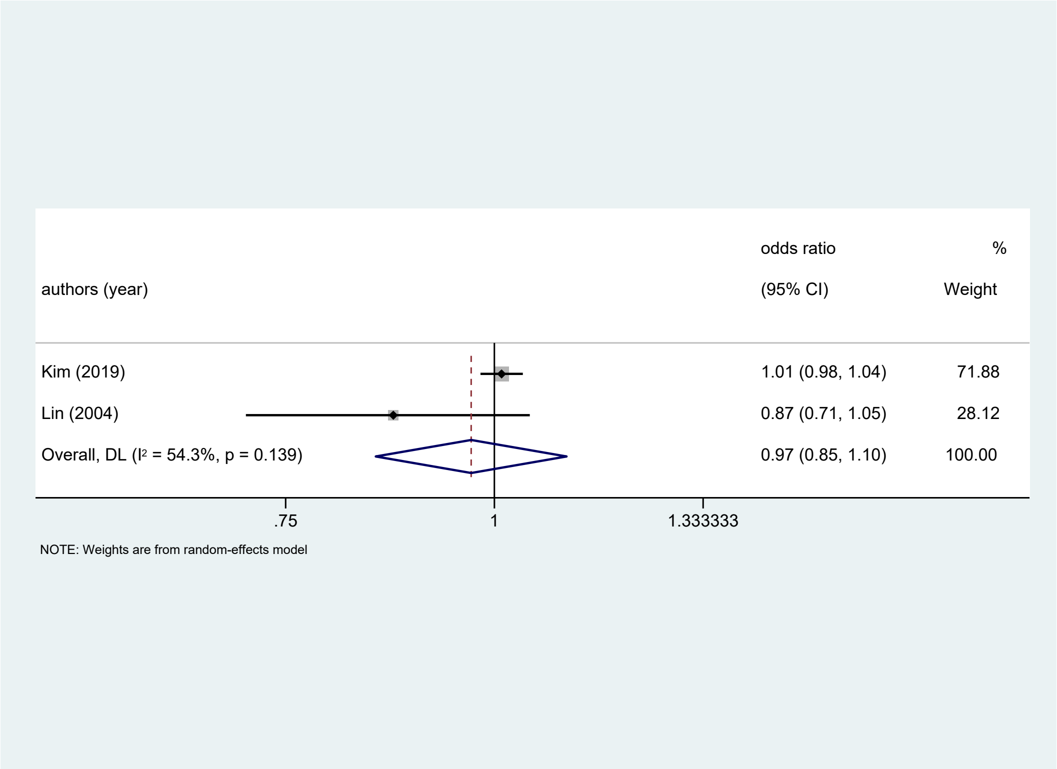

Supplementary Figure S5-c Meta-analysis of TLBW among term births with high versus low exposure to PM10 in EP

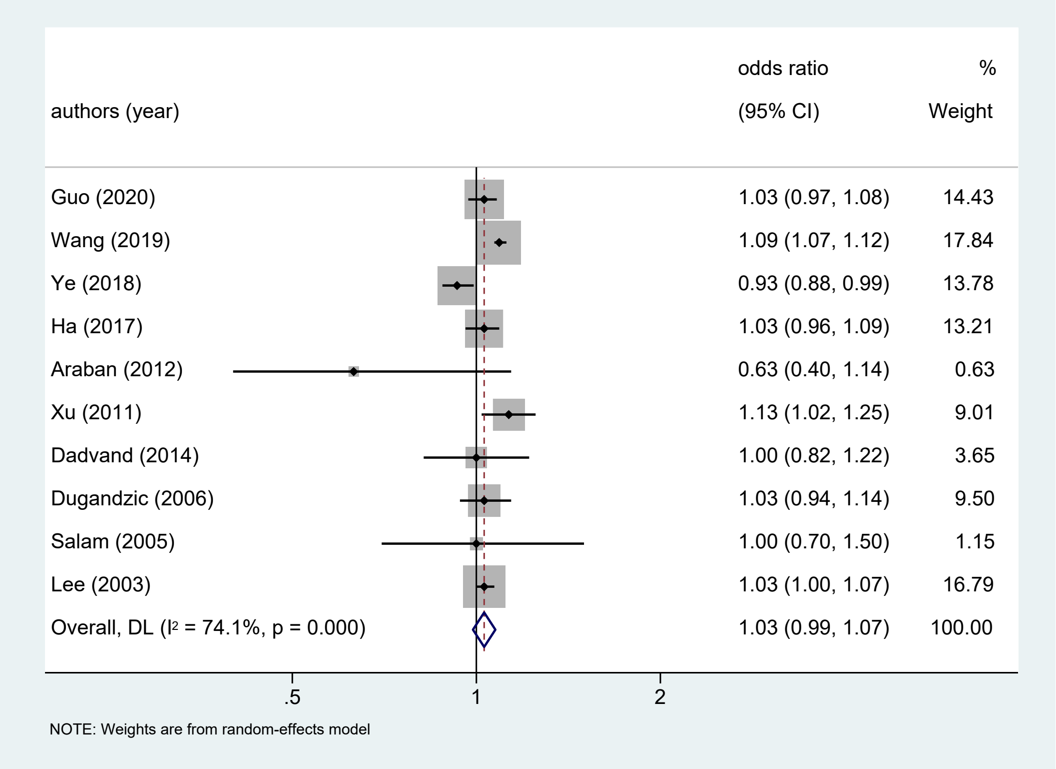

Supplementary Figure S5-d Meta-analysis of TLBW among term births with exposure to PM10 for per IQR increase in 1st

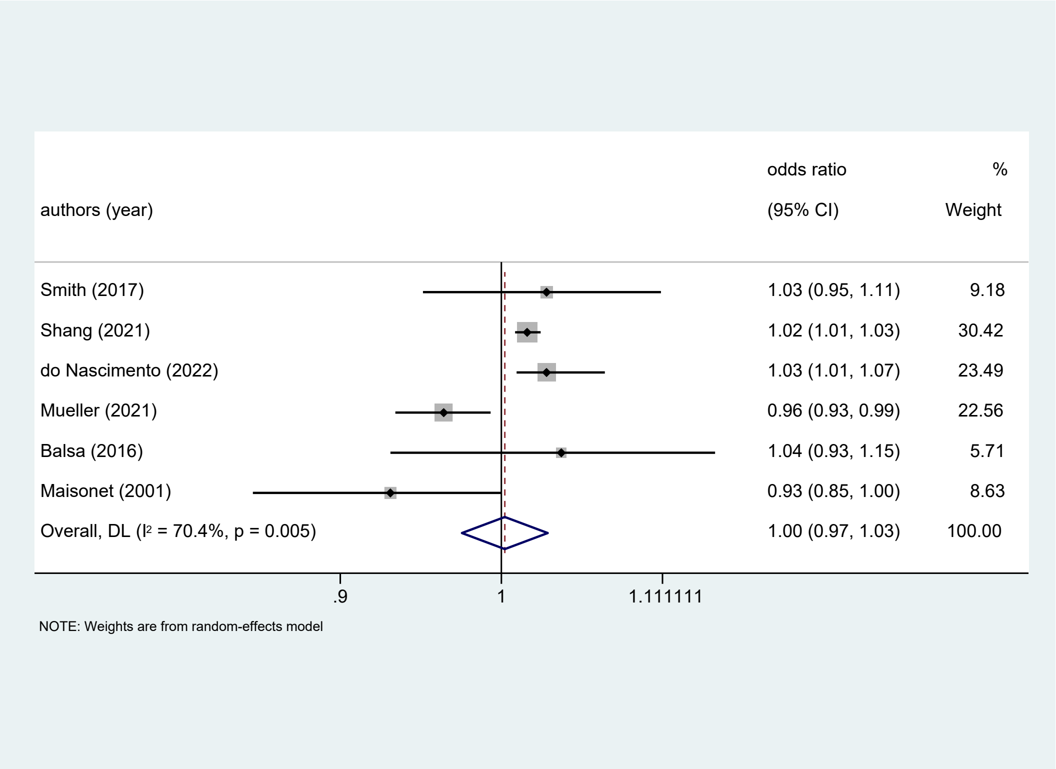

Supplementary Figure S5-e Meta-analysis of TLBW among term births with exposure to PM10 for per 10μg/m3 increase in 1st

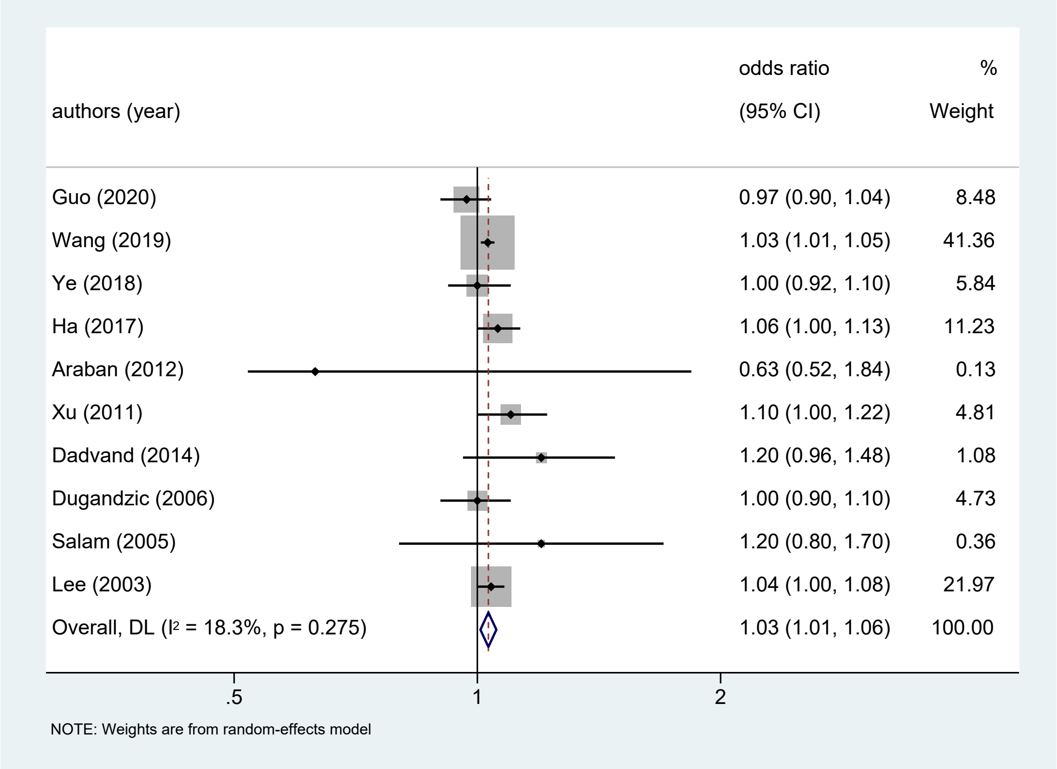

Supplementary Figure S5-f Meta-analysis of TLBW among term births with exposure to PM10 for per IQR increase in 2nd

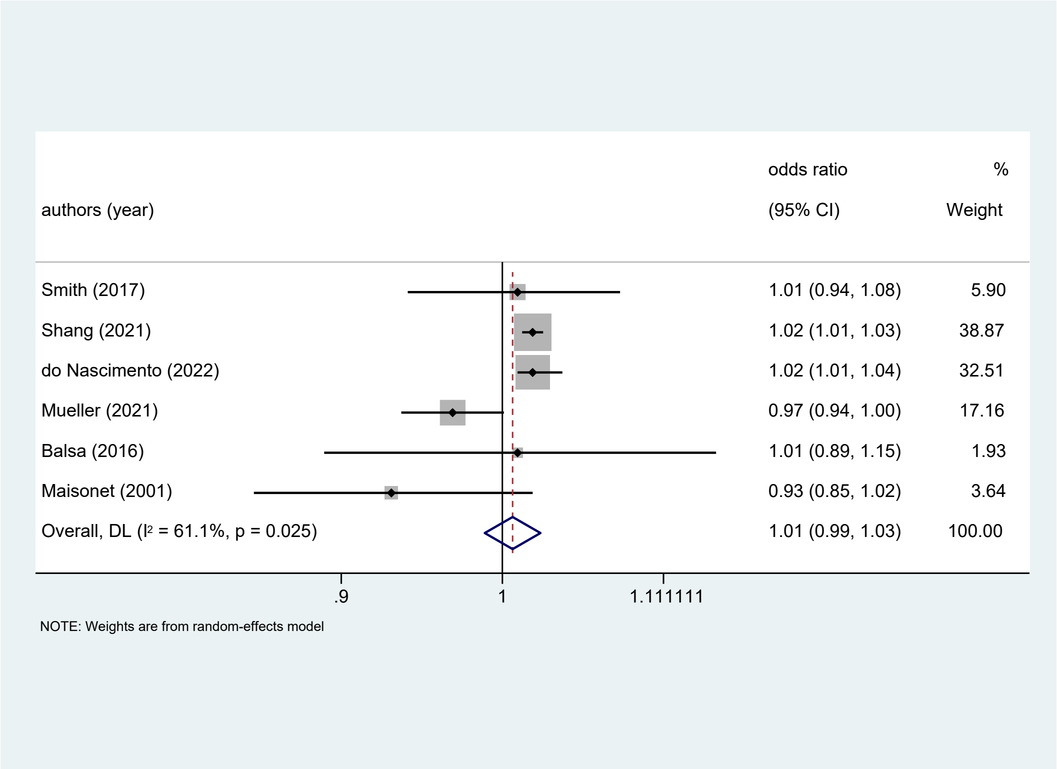

Supplementary Figure S5-g Meta-analysis of TLBW among term births with exposure to PM10 for per 10μg/m3 increase in 2nd

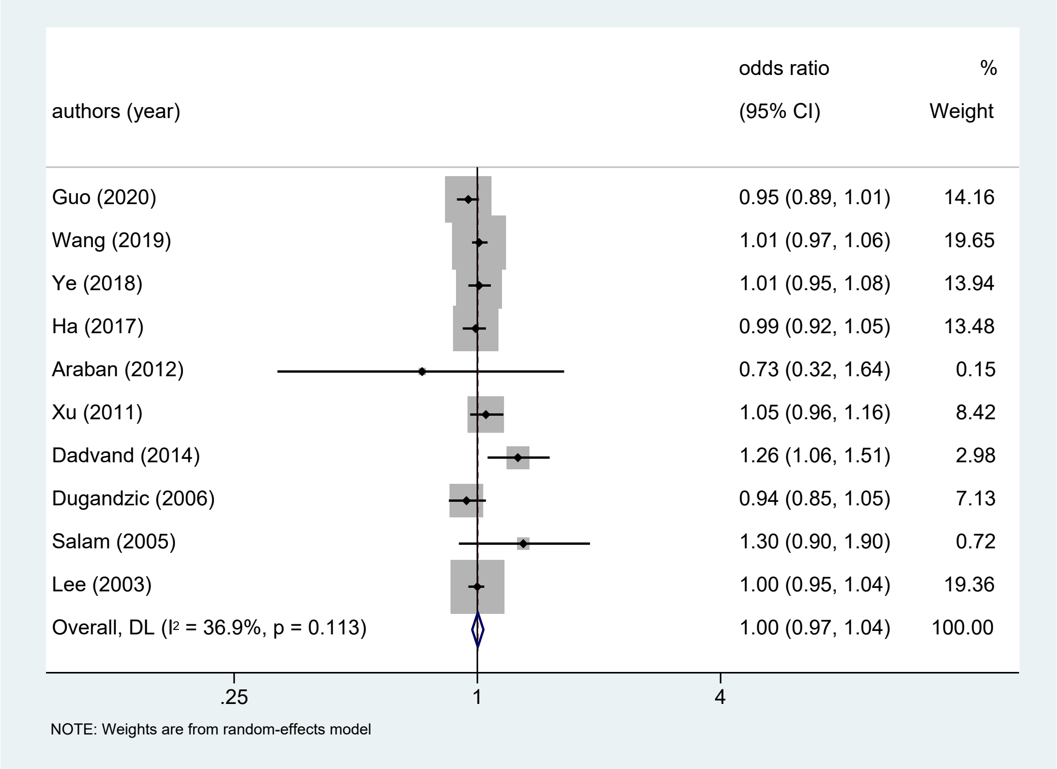

Supplementary Figure S5-h Meta-analysis of TLBW among term births with exposure to PM10 for per IQR increase in 3rd

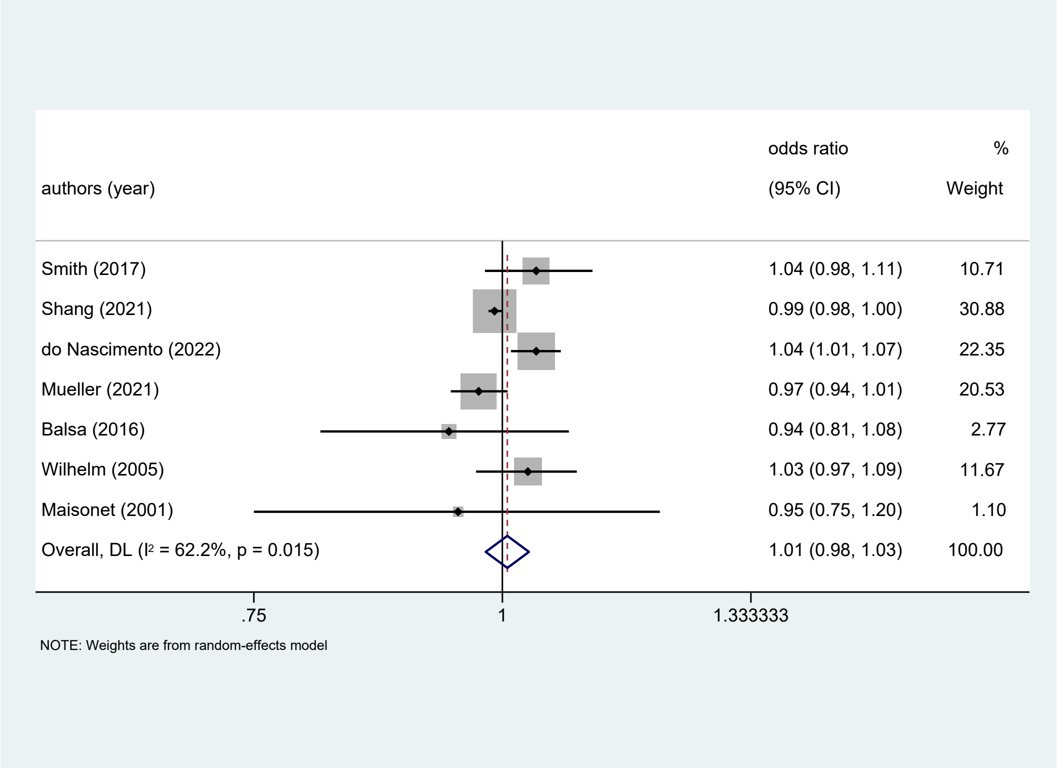

Supplementary Figure S5-i Meta-analysis of TLBW among term births with exposure to PM10 for per 10μg/m3 increase in 3rd

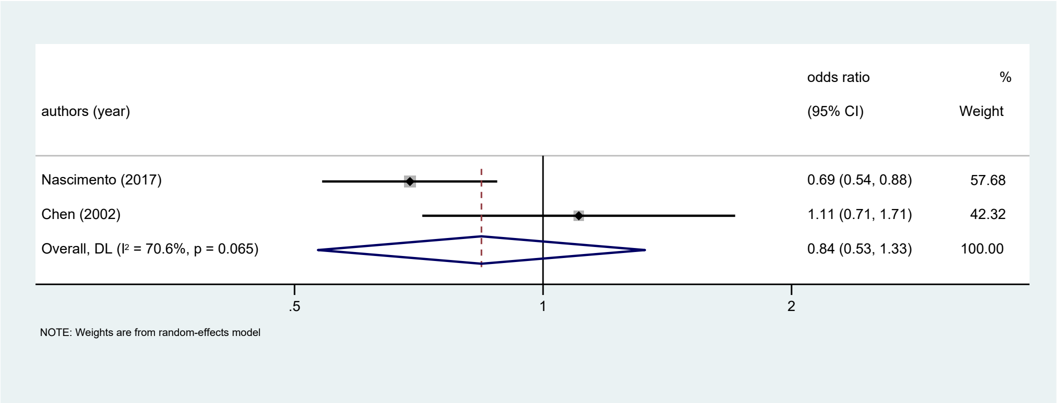

Supplementary Figure S5-j Meta-analysis of TLBW among term births with high versus low exposure to PM10 in 3rd

Supplementary Figure S6 Meta-analysis of TLBW among all births with exposure to PM10

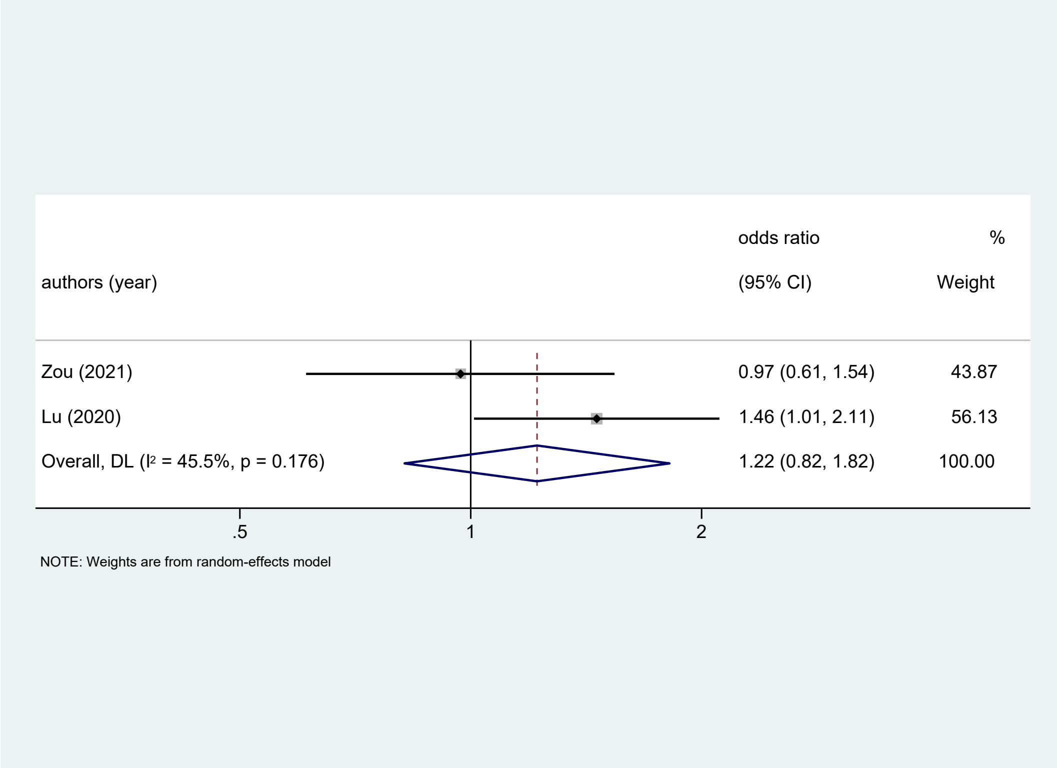

Supplementary Figure S6-a Meta-analysis of TLBW among all births with exposure to PM10 for per IQR increase in EP

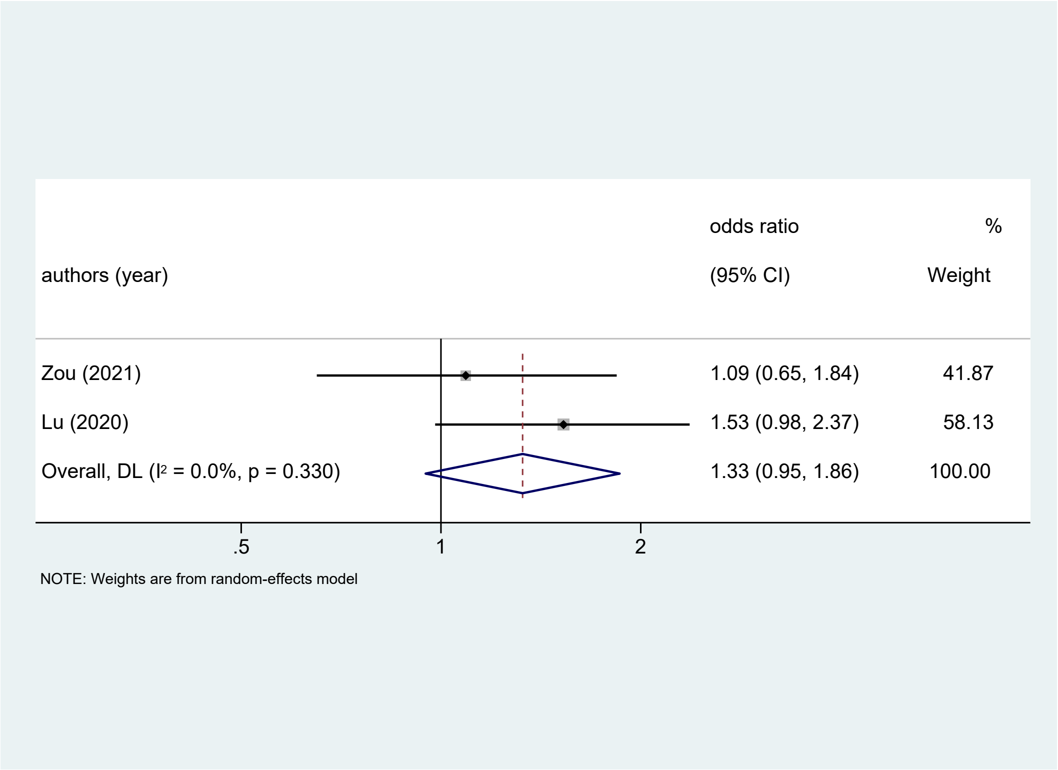

Supplementary Figure S6-b Meta-analysis of TLBW among all births with exposure to PM10 for per IQR increase in 1st

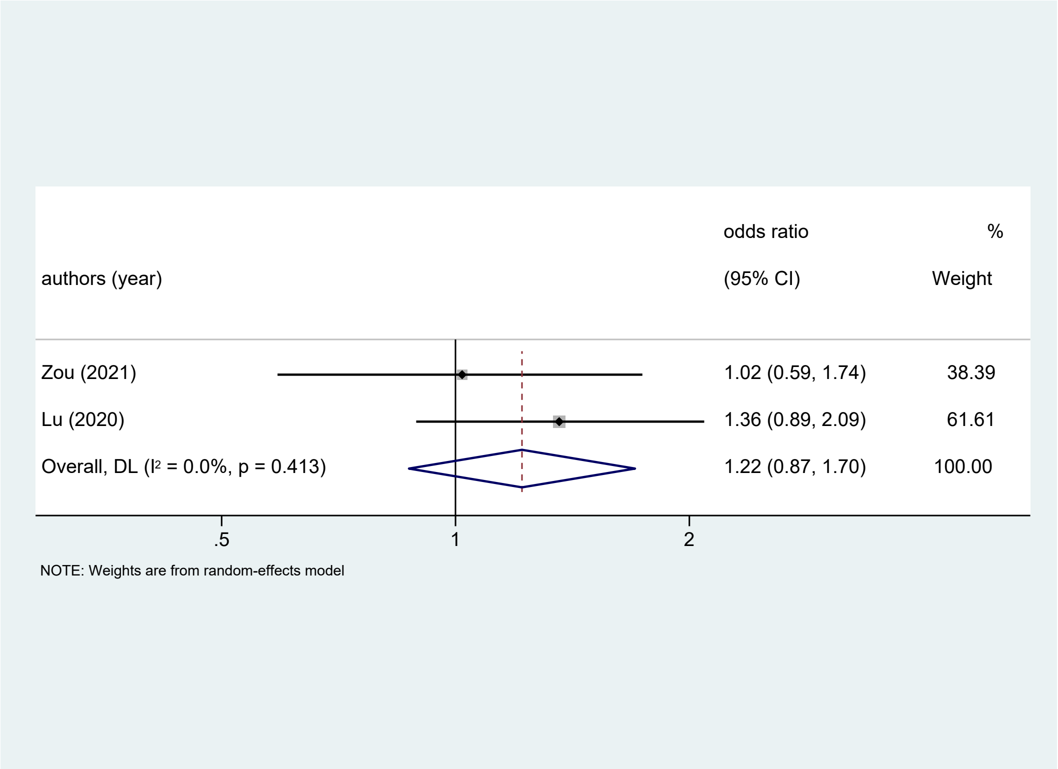

Supplementary Figure S6-c Meta-analysis of TLBW among all births with exposure to PM10 for per IQR increase in 2nd

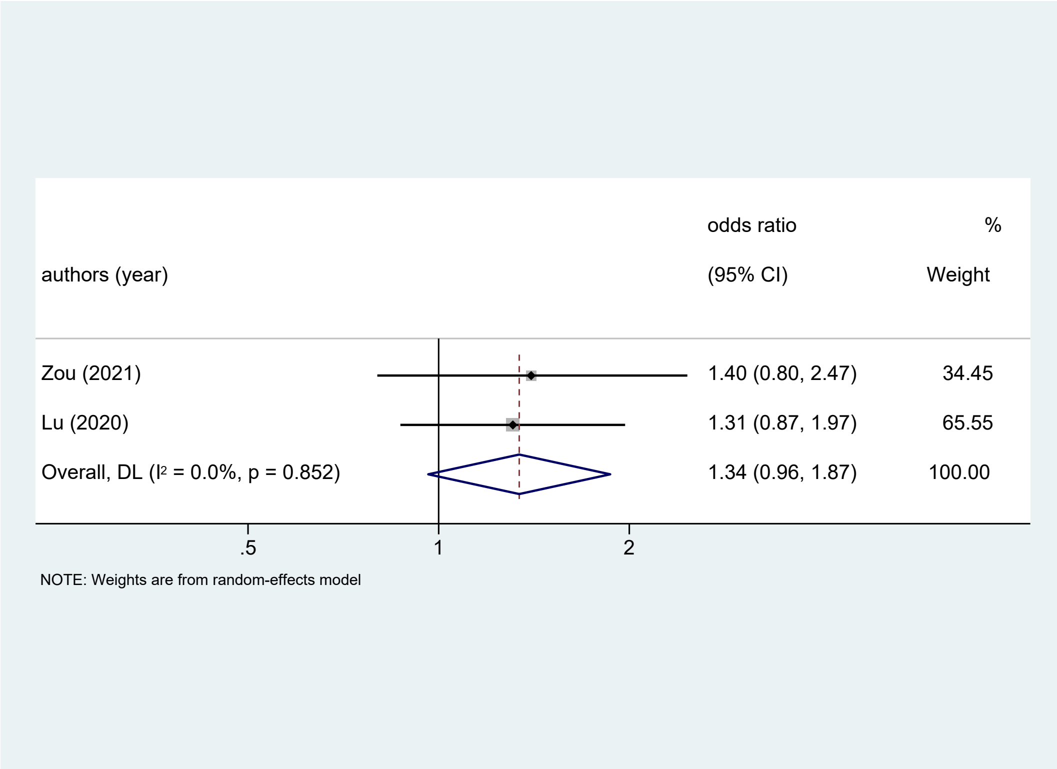

Supplementary Figure S6-d Meta-analysis of TLBW among all births with exposure to PM10 for per IQR increase in 3rd

Supplementary Figure S7 Funnel plots

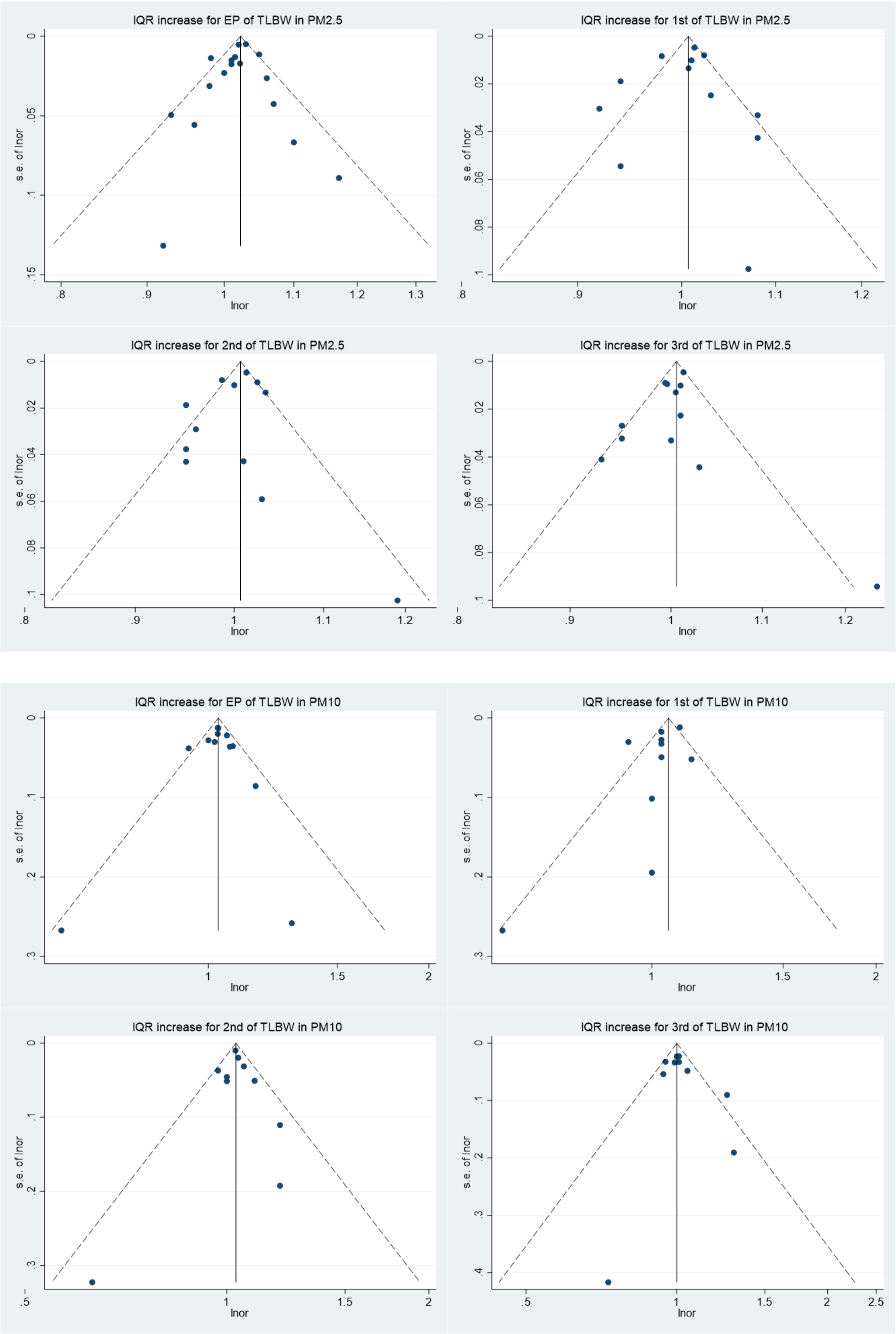

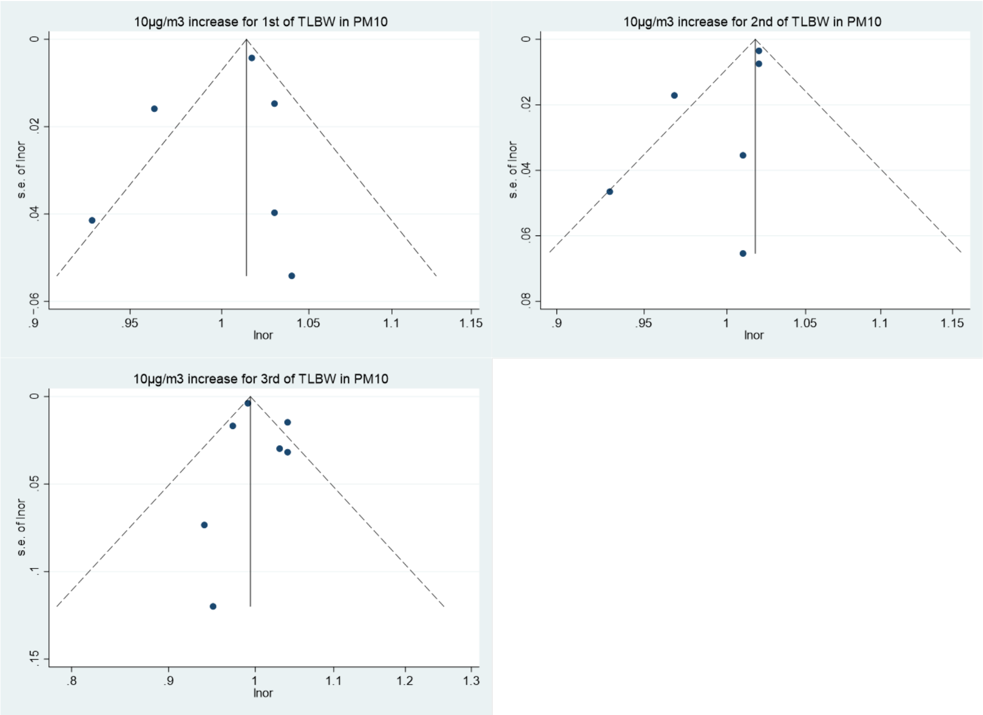
10μg/m3 increase for 1st of TLBW in PM10
s.e. of lnor
0
.02
.04
.06
.9
.95
1
1.05
1.1
1.15
lnor
10μg/m3 increase for 2nd of TLBW in PM10
s.e. of lnor
0
.02
.04
.06
.08
.9
.95
1
1.05
1.1
1.15
lnor
10μg/m3 increase for 3rd of TLBW in PM10
s.e. of lnor
0
.05
.1
.15
.8
.9
1
1.1
1.2
1.3
lnor

Supplementary Figure S8 Egger's test

IQR increase for EP of TLBW in PM2.5

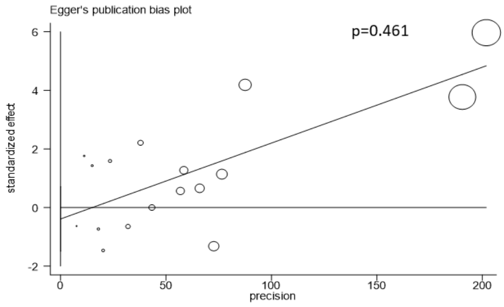

IQR increase for 1st of TLBW in PM2.5

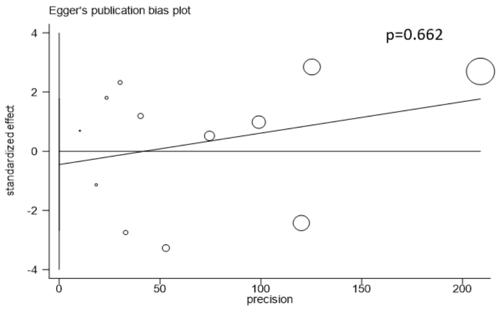

IQR increase for 2nd of TLBW in PM2.5

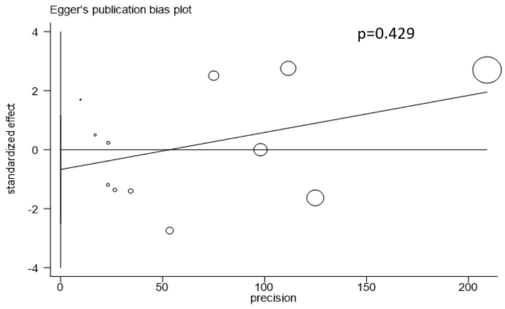

IQR increase for 3rd of TLBW in PM2.5

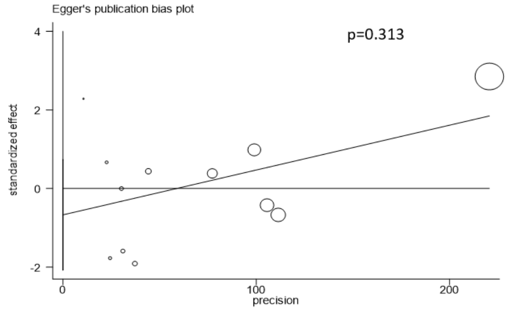

IQR increase for EP of TLBW in PM10

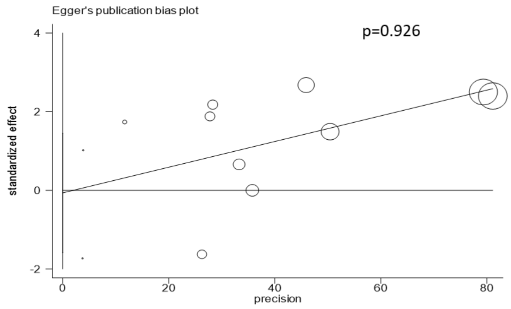

IQR increase for 1st of TLBW in PM10

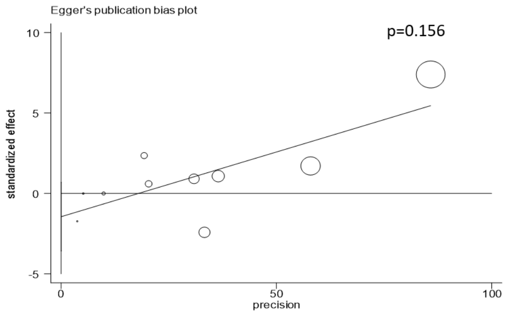

IQR increase for 2nd of TLBW in PM10

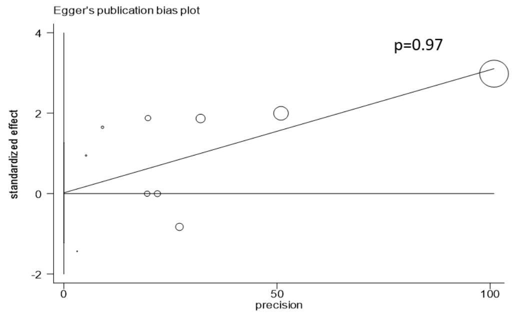

IQR increase for 3rd of TLBW in PM10

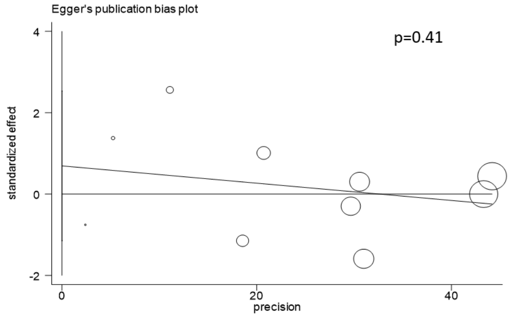

10μg/m3 increase for 1st of TLBW in PM10

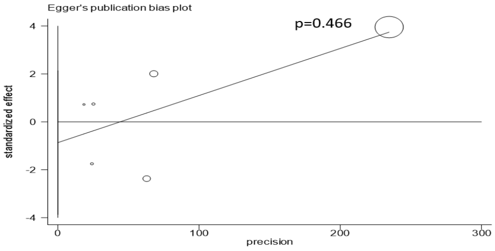

10μg/m3 increase for 2nd of TLBW in PM10

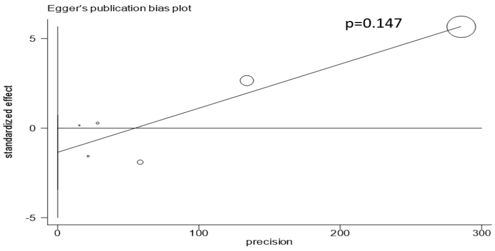

10μg/m3 increase for 3rd of TLBW in PM10

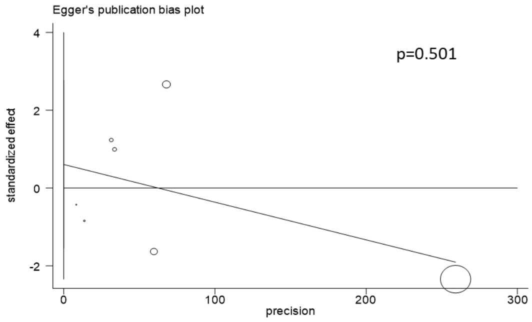

Supplementary Figure S9 Sensitivity analysis

IQR increase for EP of TLBW in PM2.5

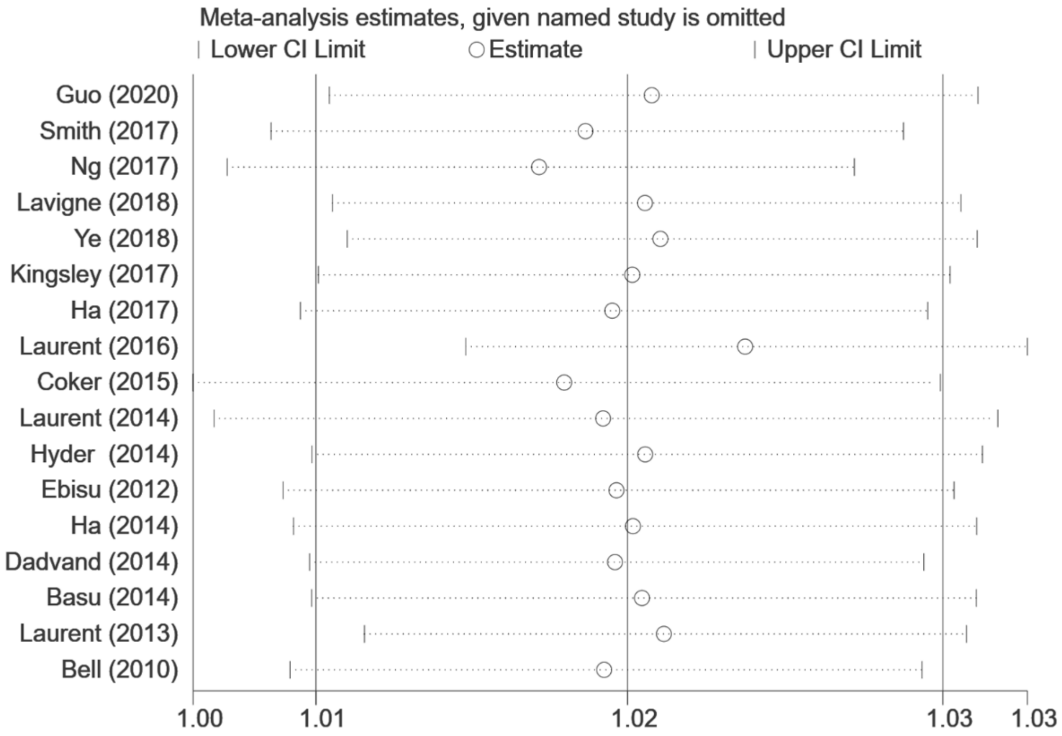

IQR increase for 1st of TLBW in PM2.5

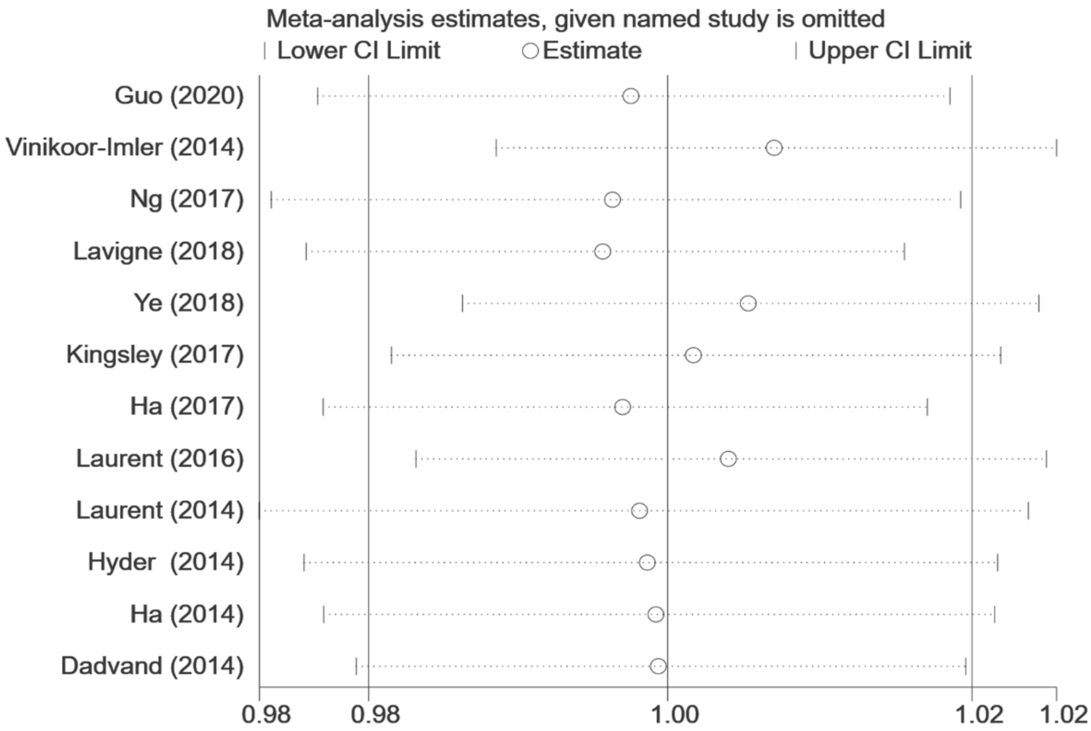

IQR increase for 2nd of TLBW in PM2.5

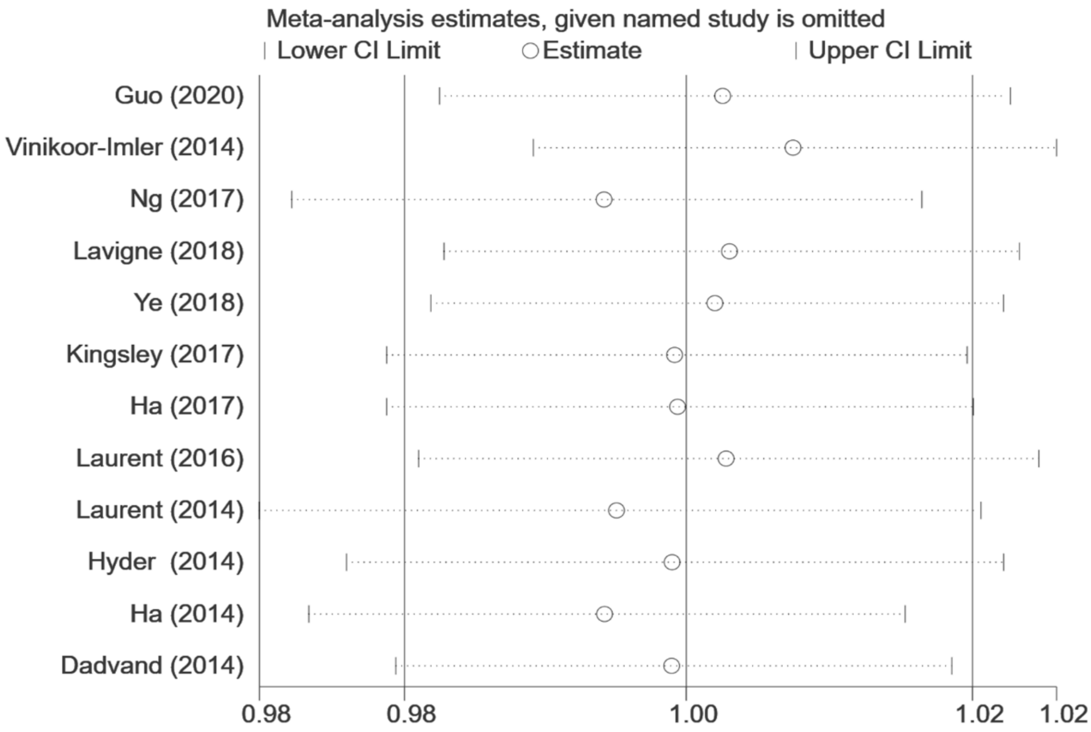

IQR increase for 3rd of TLBW in PM2.5

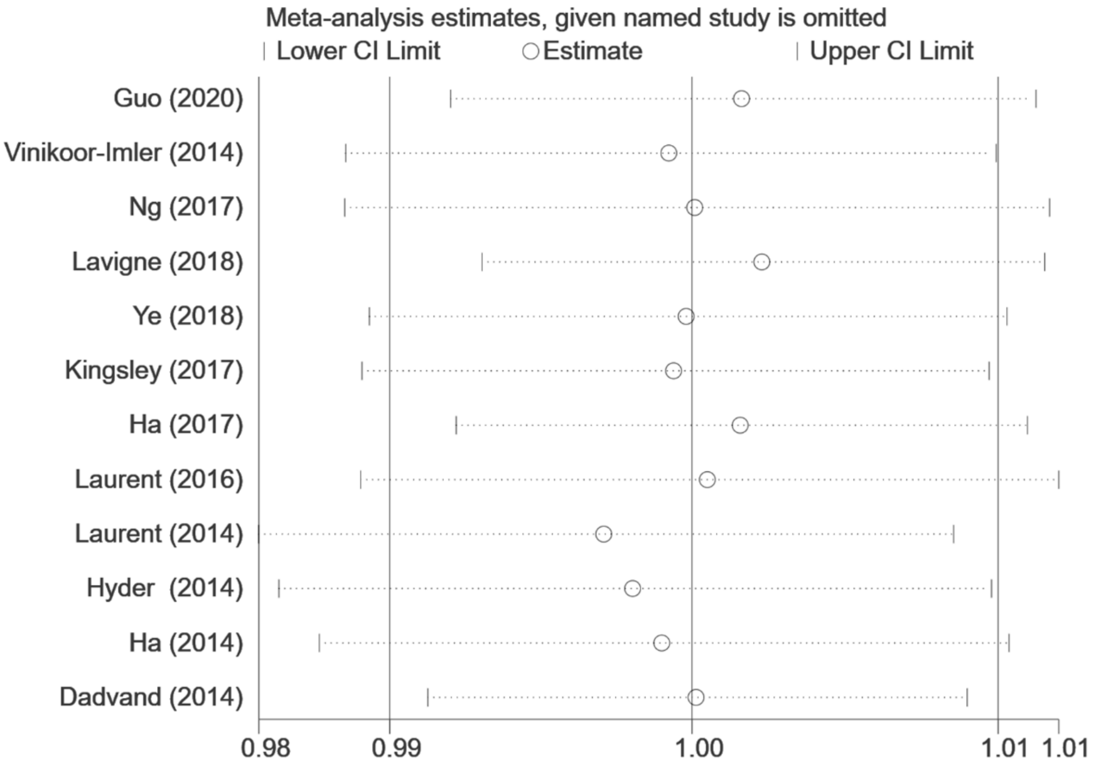

IQR increase for EP of TLBW in PM10

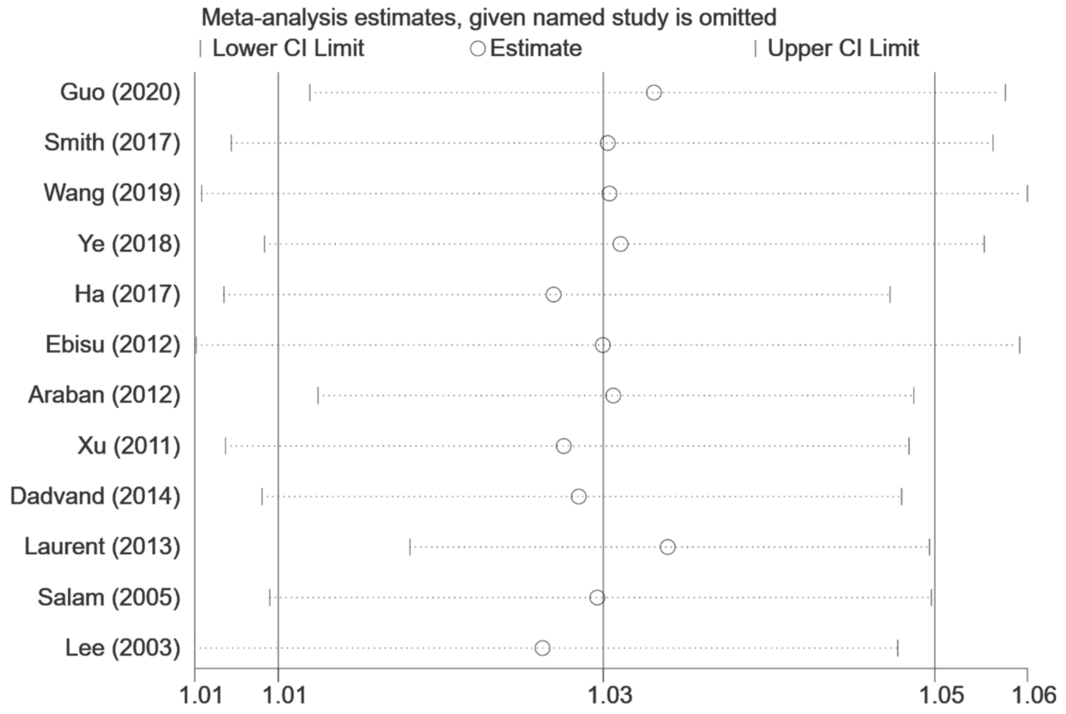

IQR increase for 1st of TLBW in PM10

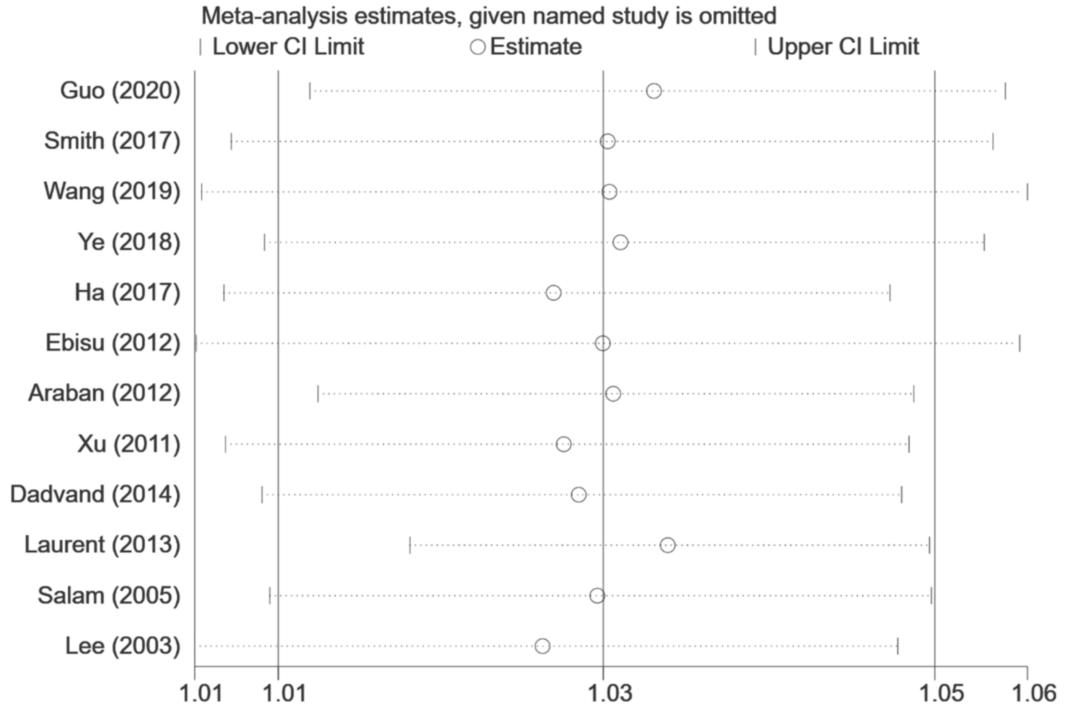

IQR increase for 2nd of TLBW in PM10

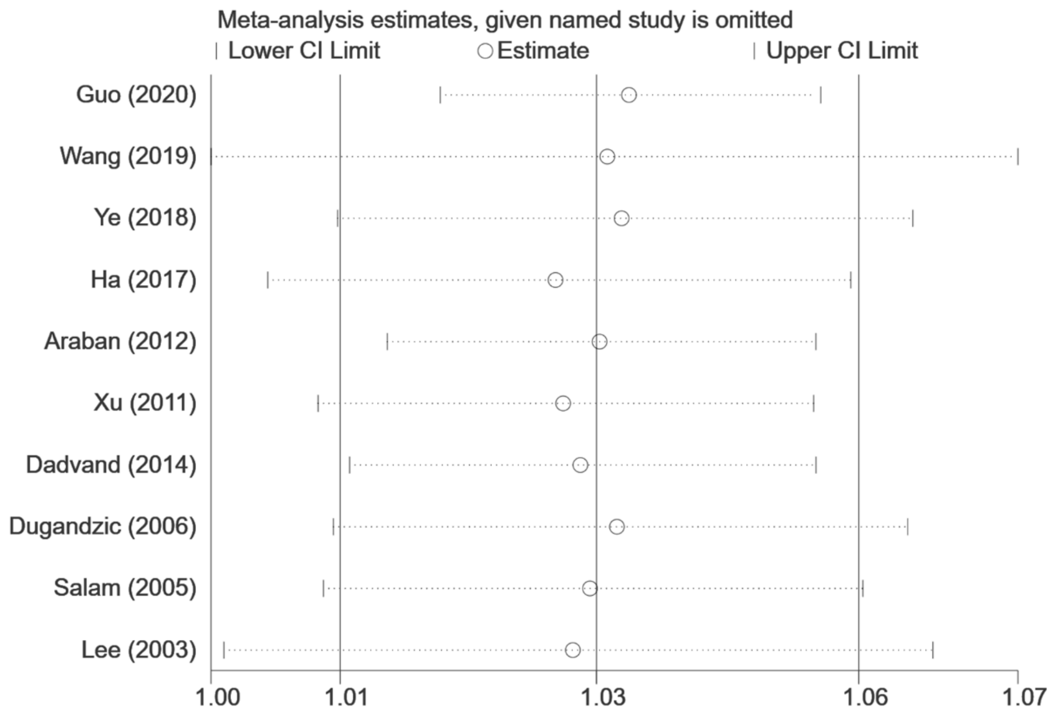

IQR increase for 3rd of TLBW in PM10

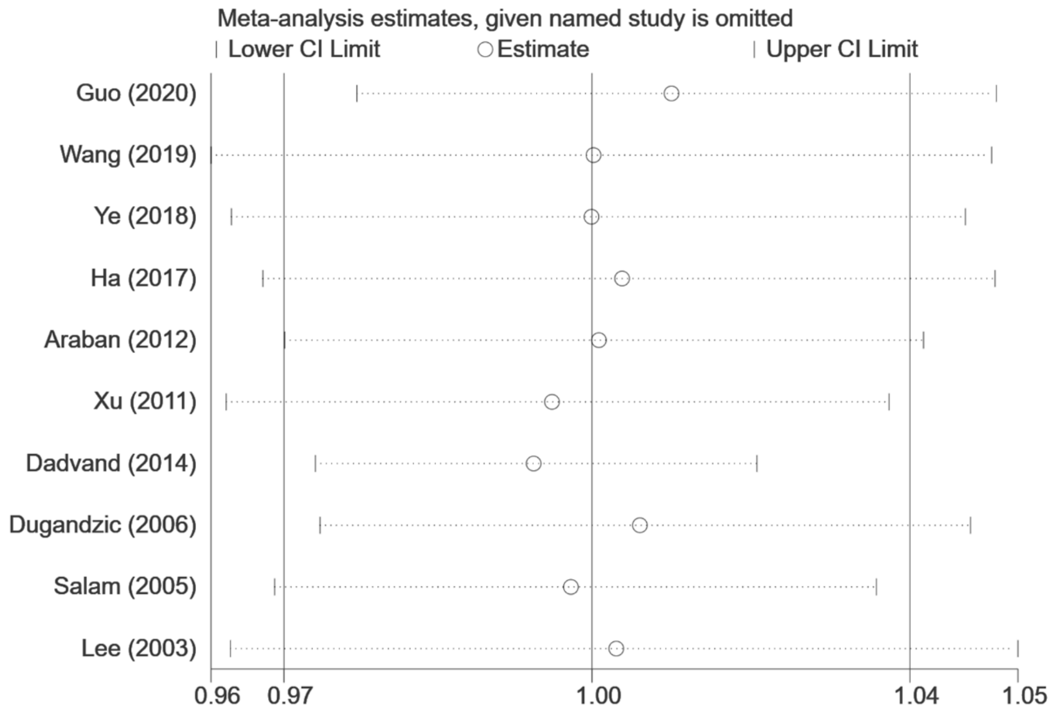

10μg/m3 increase for 1st of TLBW in PM10

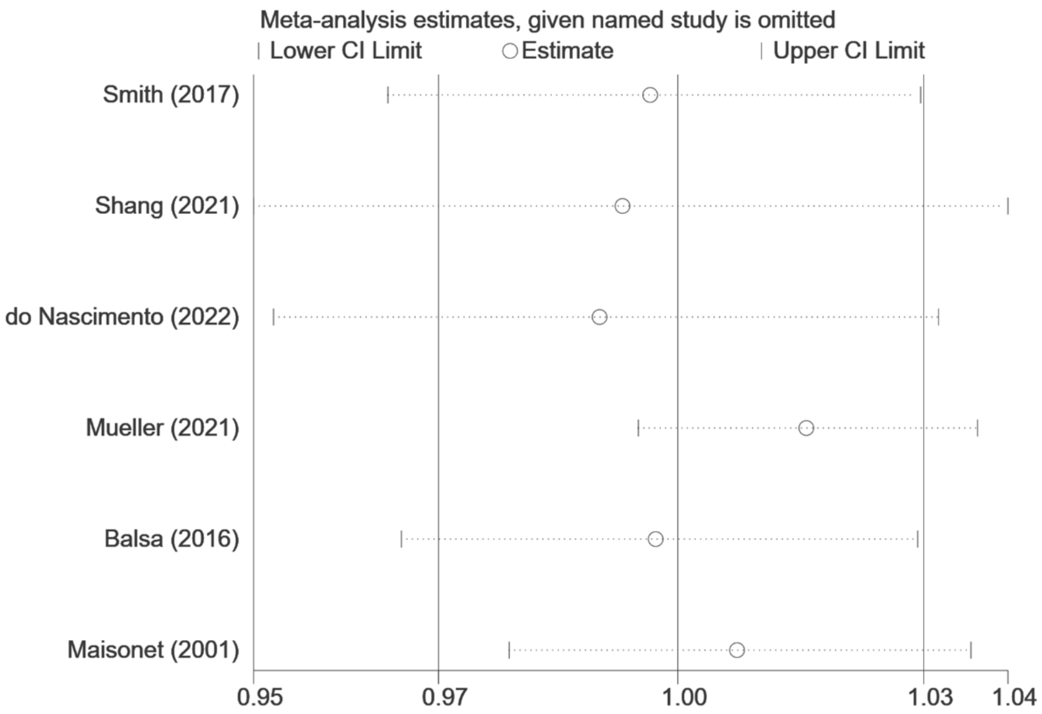

10μg/m3 increase for 2nd of TLBW in PM10

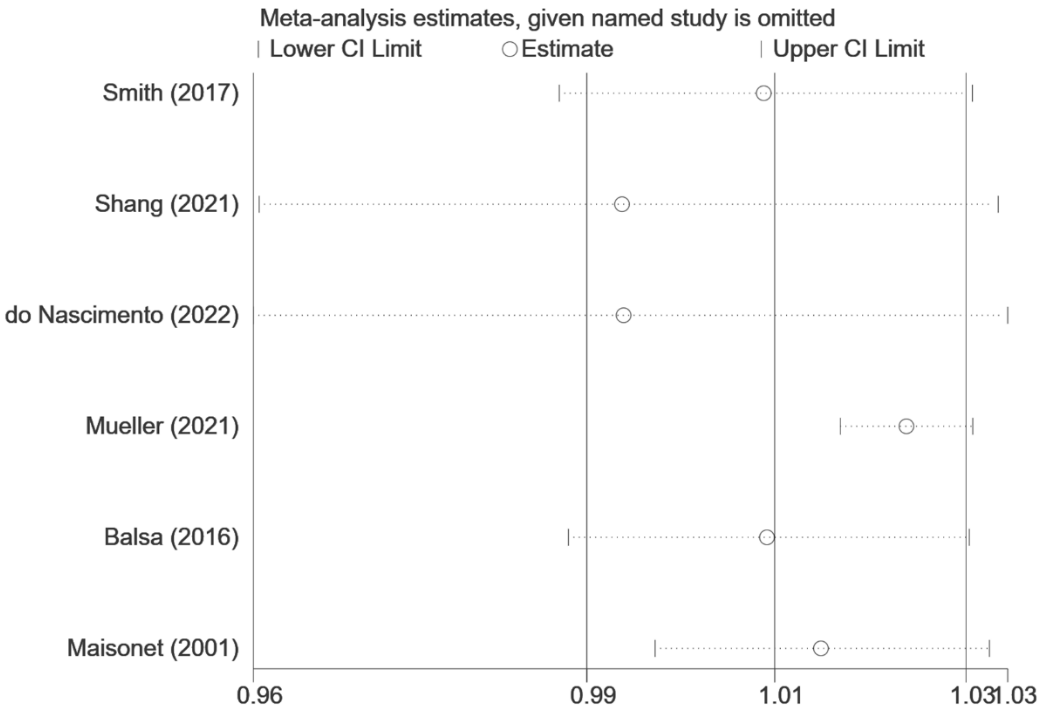

10μg/m3 increase for 3rd of TLBW in PM10

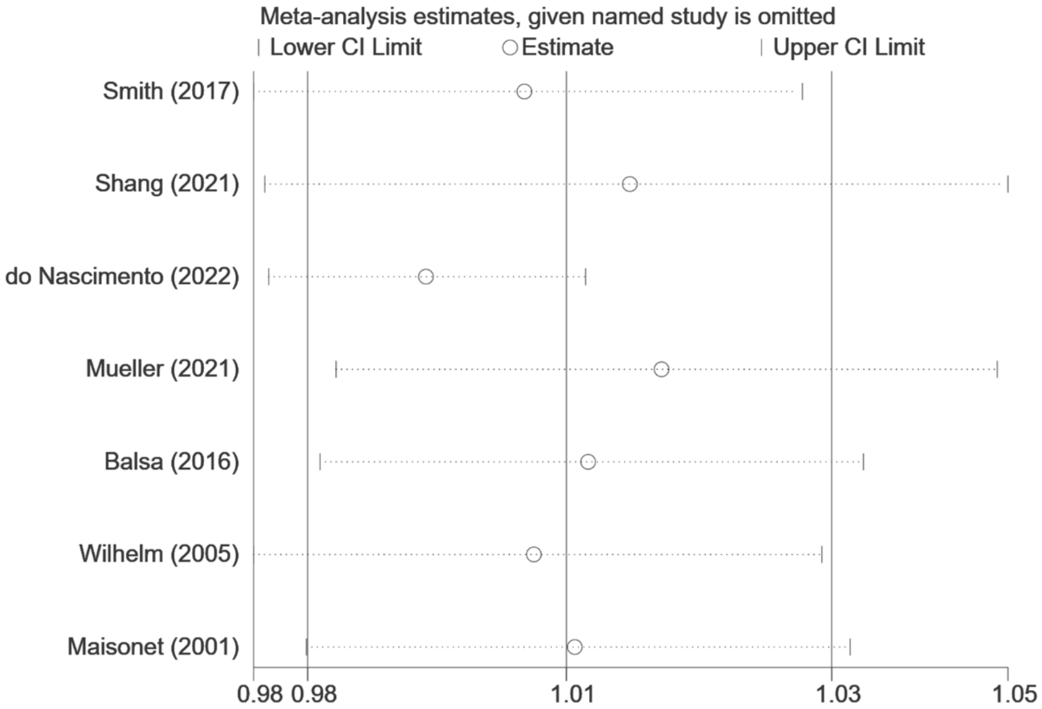

Supplement: Supplementary file 1 — Supplementary file1 (PDF 3573 KB) [file 11356_2023_26831_MOESM1_ESM.pdf]
